# Ethnobotany of Mexican and northern Central American cycads (Zamiaceae)

**DOI:** 10.1186/s13002-018-0282-z

**Published:** 2019-01-18

**Authors:** Mark Bonta, María Teresa Pulido-Silva, Teresa Diego-Vargas, Aurelia Vite-Reyes, Andrew P. Vovides, Angélica Cibrián-Jaramillo

**Affiliations:** 1 Instituto de Ecología, A.C, Apdo Postal 63, Carretera Antigua a Coatepec 351, El Haya, Xalapa, 91070 Veracruz, Mexico; 2 Laboratorio de Etnobiología, Universidad Autónoma del Estado de Hidalgo, Carretera Pachuca-Tulancingo Km 4.5 s/n, 42184 Pachuca, Hidalgo Mexico; 3 Universidad Autónoma del Estado de Hidalgo, Carretera Pachuca-Tulancingo Km 4.5 s/n, 42184 Pachuca, Hidalgo Mexico; 4 Biología Evolutiva, Instituto de Ecología, A.C, Apdo Postal 63, Carretera Antigua a Coatepec 351, El Haya, Xalapa, 91070 Veracruz, Mexico; 5 Laboratorio Nacional de Genómica para la Biodiversidad (Langebio), Unidad de Genómica Avanzada, Centro de Investigación y de Estudios Avanzados del Instituto Politécnico Nacional, Km 9.6 Libramiento Norte Carretera León Irapuato-León, 36824 Guanajuato, Mexico

**Keywords:** Cycads, *Zea mays*, Community-based conservation, Maize gods, Zamiaceae, Teenek Maya, Nahua, Ethnobotany of cycads, Neurotoxicity of cycads

## Abstract

**Background:**

This study documents cycad-human relationships in Mexico, Belize, Guatemala, El Salvador, and Honduras over the last 6000 years. The impetus was acute need for a better understanding of previously undocumented uses of cycads in this region, and the need to improve cycad conservation strategies using ethnobotanical data. We hypothesized that cycads are significant dietary items with no long-term neurological effects, are important to religious practice, and contribute to cultural identity and sense of place, but that traditional knowledge and uses are rapidly eroding. Guiding questions focused on nomenclature, food and toxicity, relationships to palms and maize, land management issues, roles in religious ceremony, and medicinal uses, among others, and contributions of these to preservation of cycads.

**Methods:**

From 2000 to 2017, the authors conducted 411 semi-structured ethnographic interviews, engaged in participant-observation in Mexican and Honduran communities, and carried out archival research and literature surveys.

**Results:**

We documented 235 terms and associated uses that 28 ethnic groups have for 57 species in 19 languages across 21 Mexican states and 4 Central American nations. Carbohydrate-rich cycads have been both famine foods and staples for at least six millennia across the region and are still consumed in Mexico and Honduras. Certain parts are eaten without removing toxins, while seed and stem starches are detoxified via several complex processes. Leaves are incorporated into syncretic Roman Catholic-Mesoamerican religious ceremonies such as pilgrimages, Easter Week, and Day of the Dead. Cycads are often perceived as ancestors and protectors of maize, revealing a close relationship between both groups. Certain beliefs and practices give cycads prominent roles in conceptions of sense of place and cultural heritage.

**Conclusions:**

Cycads are still used as foods in many places. Though they do not appear to cause long-term neurological damage, their health effects are not fully understood. They are often important to religion and contribute to cultural identity and sense of place. However, because most traditional knowledge and uses are rapidly eroding, new community-based biocultural conservation efforts are needed. These should incorporate tradition where possible and seek inspiration from existing successful cases in Honduras and Mexico.

**Electronic supplementary material:**

The online version of this article (10.1186/s13002-018-0282-z) contains supplementary material, which is available to authorized users.

## Background

This study of historical and contemporary traditional roles of cycads in human societies documents and analyzes fading uses and knowledge, and places emphasis on aspects of cycad ethnobotany that can aid conservation strategies. Cycads (Order Cycadales) are dioecious, cone-bearing gymnosperms with 351 known species of Miocene origin [1] currently distributed in tropical and subtropical regions. They are the world’s most threatened group of plants [2]. In Mexico and northern Central America (MNCA), of the 71 recognized native species, 21 are critically endangered, 23 endangered, and the rest in lesser risk categories, with only 1 least concern species [2]. They are threatened by cattle ranching, habitat destruction, the illicit nursery trade, and climate change [3, 4]. Though MNCA cycads are protected on paper in response to CITES, additional on-the-ground measures are necessary. MNCA governments have engaged conservationists, scientists, existing parks, and local communities to protect cycads, but results have been spotty. Ex situ efforts such as community nurseries incentivizing habitat conservation through seed harvest, propagation, legal plant sales, and reintroduction have been marginally successful due to bureaucratic insufficiency and lack of marketing and follow-up studies [5]. Recognition, rescue, and interpretation of cycad ethnobotanical knowledge, a collaborative process between knowledge-holders and outside experts, is vitally important to help community-based biocultural conservation initiatives, and revalorize sense of place and cultural identity, effectively providing a more broad-based and deeply rooted way to protect and sustain cycads, in particular in Latin America [6], as examples later in this article from Chiapas, northeastern Mexico (NEMX), and Honduras show [7, 8].

### Misconceptions and avoidance of cycad ethnobotanical research

A fundamental impetus for this study was the lack of attention to cycads in the social sciences and the proliferation of false assumptions about them. While biologists have studied cycads extensively [9, 10], social scientists have perceived them at best as minor and marginal food resources [11, 12]. At worst, their use as food is often assumed to cause long-term neurological damage to humans, even though attempts to establish a causal link between cycad consumption and neurodegenerative diseases such as ALS-PDC are inconclusive [13, 14].

The “cycad hypothesis,” an assertion that the putative long-term neurotoxicity of *Cycas micronesica* (formerly *C*. *circinalis*) in the US territory of Guam is the cause for litigo-bodig (a local form of ALS-PDC syndrome), provided post-1960 researchers the justification to study a neurological disease cluster among Chamorros who had consumed cycads heavily as recently as the 1940s and, some studies suggested as a direct result, suffered from extremely high rates of litigo-bodig [15]. However, studies of Chamorros did not take numerous environmental and genetic factors into consideration, chief among them other environmental toxin sources as well as the fact that cycad seeds, to be detoxified, were in the 1940s steeped in water-filled, lead paint-coated oil drums. Despite the lack of proof [14], the assertion that Chamorros’ consumption of cycads, or of fruit bats that consumed cycads (through putative biomagnification of the toxins in bat muscle tissue [16]), causes long-term neurodegeneration came to be the scientific consensus. Purported but never vindicated reports of cycad neurodegenerative links in Honshu and New Guinea broadened the “cycad hypothesis” to the Western Pacific region [13].

Extensive historical reviews of cycad ethnobotany appeared in the mid-twentieth century, highlighting their uses as staple foods and famine resources as well as for starch production, alcohol, medicine, narcotics, and ceremony [15, 17]. While some cycad parts were eaten raw or with little processing, seed and stem starch necessitated complicated, multi-day procedures to render them safe. And though humans have consumed cycads, often in large quantities, since the Pleistocene [18], other than the unproven cases in three Western Pacific locations, long-term neurodegenerative effects have never been documented anywhere else. Cycad-eating cultures universally know that some parts of the plants are acutely toxic and can cause serious sickness and death, and thus that their toxins must be rendered safe before consumption. Nevertheless, the engrained support for the idea that cycads cause long-term neurodegeneration appears to be the primary reason for the dearth of ethnobotanical studies after the 1960s. Simply put, the dominant assumption is that humans consume cycads only as a last resort because they are so toxic, and therefore they can never have been important in human diets, e.g., [19]. One of this study’s primary aims was to test this assertion, as it can affect any attempt to promote community-based conservation based on preservation of traditional practices related to their importance as food.

### Brief summary of post-1960 cycad ethnobotany

Apart from significant work in Australia, and the voluminous Western Pacific toxicity studies, modern cycad ethnobotanical publications have been minimal and scattered. In summary, (1) cycads are still used widely for food and other reasons in the Americas [20, 21], sub-Saharan Africa [22], Asia [23, 24], Australia [25–27], and some Pacific islands, though rarely as extensively as reported historically [15, 17]. Though we are far from identifying all cycad-eating societies, it is clear that in many cases, users’ poverty and remoteness from markets is directly related to whether or not they still consume the plants, given increased availability of less risky and labor-intensive foods with modernization. Cycad consumption is not necessarily a direct function of dire need, however, because they are also eaten for their value as sacraments, casual snacks, and representatives of valued cultural heritage [28]. (2) Cycads are high-status plants associated with power and conferring prestige, e.g., in Vanuatu [29], among the Yanyuwa of Arnhem Land, Australia [26], in Panama [22], and in South Africa [22]. As a result, uses and knowledge of cycads have often been kept secret from outsiders. (3) Leaves are still used for religious ceremonies in numerous contexts across the world, and have manifold ritual meanings [22, 28]. (4) Cycads and their parts are traded licitly and illicitly both from nursery stock and by extraction from the wild.

### Cycad biology in Mexico and northern Central America

**Fig. 1:**
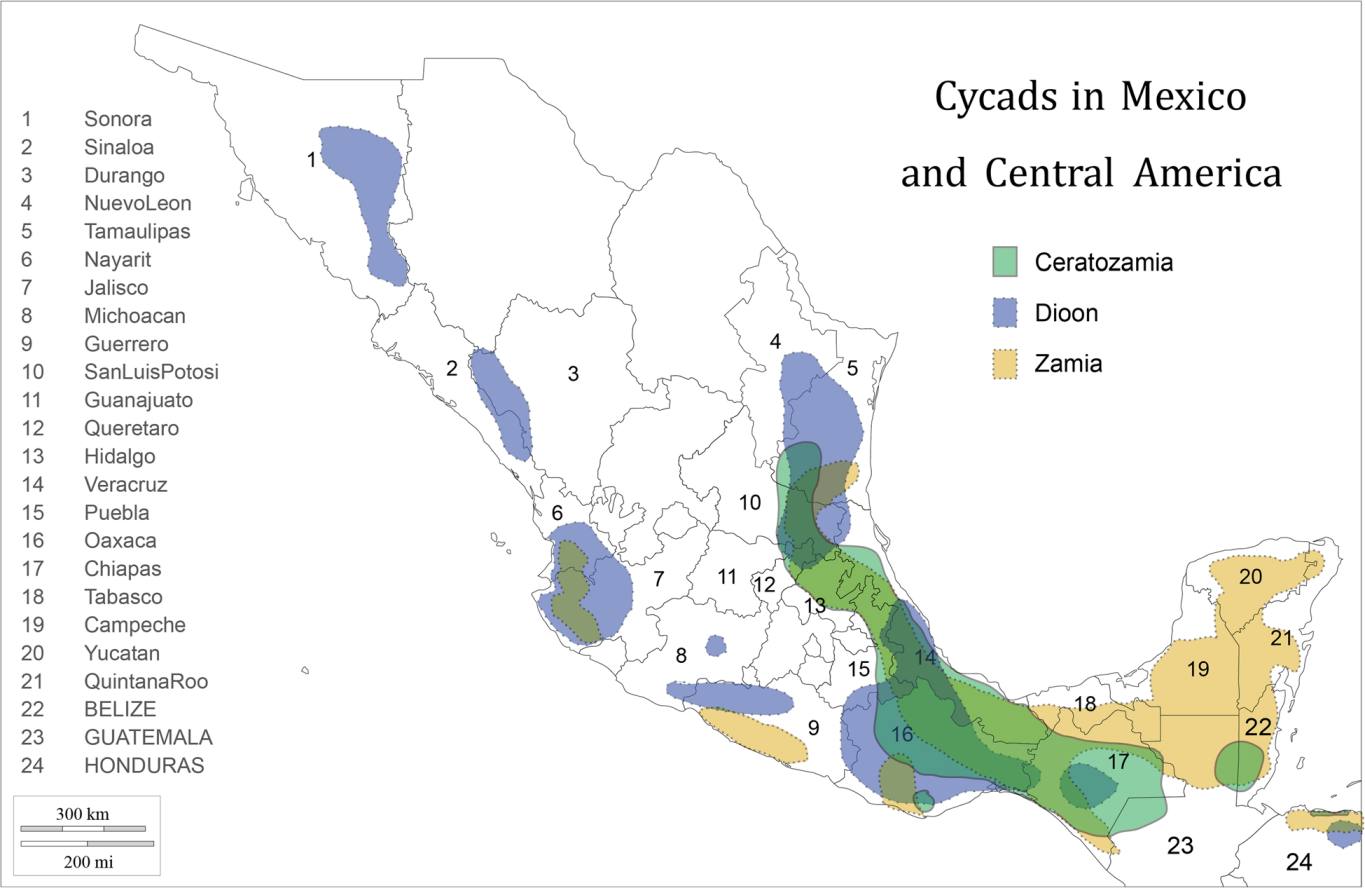
Cycads in Mexico and Central America: distribution by genus

MNCA cycads belong to three genera (*Dioon*—15 species; *Ceratozamia*—31 species; *Zamia*—25 species) in the family Zamiaceae [2] (Fig. 1). All but one *Dioon* and two *Ceratozamia* species are Mexican endemics, while *Zamia* is found throughout New World tropical and subtropical regions. Of the three genera, *Dioon* spp. are typically the largest, with stems to 10 m in length and 50 cm in girth or branching at the base. They are thought to live for over two millennia [30]. *Dioon* seed cones on female plants can exceed 25 kg and 50 cm in length [31]. While some *Zamia* and *Ceratozamia* spp. have erect stems of a meter or more, others have subterranean stems referred to locally as “roots” (*raices*); their seed cones range upward from the dimensions of a small maize cob and may weigh several kilograms.

Male cycads produce cones with pollen that Curculionid beetles transport to receptive nearby female cones [32–35]. Female cones take many months to reach maturity and dehisce [36]. Seeds are dispersed by gravity and by vertebrates transporting and caching them for their sarcotestas (fleshy, sugary outer seed coverings) [37]. In areas where people eat them, cycad cones are usually harvested while still on the stems, and few seeds are left behind. The timing of coning is irregular and apparently depends on a range of factors, including fire, which may occur more than once a year in a single location. Fire stimulates new leaf and cone production through nutrient release [30, 38]. Cycads generally produce a new leaf flush annually or biennially; the leaf scars left on the trunks of larger species can be used to establish age [9, 30].

MNCA cycads tolerate diverse environments, and are most often found on steep, rocky terrain with low nutrient availability. Historical accounts mention widespread lowland cycad populations that have since been wiped out for plantation agriculture and cattle ranching [39, 40]. Cycads grow in grasslands, pine, oak and pine-oak woodlands, tropical dry forest, arid deciduous scrub, and thick, moist evergreen forest [41]. Their unique adaptations include nitrogen-fixing coralloid roots [10], extreme fire- and drought-tolerance, and the capacity to produce numerous offshoots and clones, in some cases as a result of damage. Overall, cycads are remarkably persistent in the face of damage, where not subject to massive anthropogenic destruction [10].

### Aims of the study

The aims of the research that comprises this study were (1) use ethnographic and ethnohistorical methods to collect, consolidate, and streamline a fragmentary, confusing, and anecdotal ethnobotanical knowledge base; (2) elucidate important questions with major ramifications for understanding human relationships to cycads in MNCA and to a certain extent elsewhere in the world; and (3) provide a knowledge base for community-conservation strategies. We were guided by the following questions: (1) What roles have cycads played in human diets? (2) What are the adverse health effects of cycad consumption on people? (3) What are the relationships of cycads to other plants such as palms and maize (given the indication of strong ties to cycads as evinced in nomenclature and previous studies)? (4) What are the meanings of ritual cycad use in Catholic and syncretic ceremonies? (5) How are cycads incorporated into traditional land-use management? (6) How do cycads contribute to sense of place and cultural identity? (7) How are minor uses such as for ethnomedicine related to the other questions? (8) Can human knowledge of cycads be incorporated into conservation strategies? We formulated the following hypotheses: (1) cycads have been and remain important food plants despite their toxicity. (2) Traditional cycad food-processing techniques prevent long-term effects on human neurological health. (3) Cycads are integral to traditional religious practice, particularly because of their close relationships to palms and maize. (4) The importance of food, ceremony, and minor uses have led to heightened sense of place and cultural identity and stimulated traditional protection measures designed to help preserve cycads. (5) Cycad ethnobotanical knowledge, uses, and traditional protection measures are being rapidly eroded across the entire region.

## Methods

### Overview

To answer our questions and address our hypotheses, we broadly followed standard qualitative ethnobotanical methods [42, 43]. AR and TD completed ethnobotanical theses [44–46] in NEMX among Nahua, Teenek, and mestizo peoples, while the other authors undertook additional fieldwork there and elsewhere. We conducted a thorough literature review and communicated personally with cycad experts who had observed local uses. Given the vast and diverse nature of our largely exploratory and descriptive undertaking, we did not intend for our qualitative data to bear rigorous statistical analysis. We focused our research where cycads are known and significant to local people (Table 1). Exploratory interviews established whether people still had detailed knowledge of cycads; if so, we carried out in-depth interviews.

**Table 1:** Ethnographic interviews summary

I.^a^	Yr|s^b^	#^c^	State (Mx)	Regions	Ethnic groups	Languages^d^
MB	03	4|2	Chiapas	Suchiapa	Chiapanec, Mestizo	Spanish
AR, MB, TD, TP	08,09, 10,11, 15,16	11|157	Hidalgo	Sierra Madre Oriental; Huasteca	Mestizo, Nahua, Otomí-Ñuhu, Tepehua	Nahuatl, Otomí-Ñuhu, Spanish, Tepehua
MB	16	17|6	Jalisco	Sierra Madre Occidental; Costa Pacífica; Tuito	Mestizo	Spanish
MB	16	15|3	Nayarit	Jalcocotan; Sierra del Nayar	Cora, Mestizo	Cora, Spanish
MB, TD	08,17	30|11	Oaxaca	Sierra Norte de Puebla; Sierra Sur; Papaloapan Valley; Valles Centrales	Chatino, Chinantec, Chontal de Oaxaca, Mazatec, Mestizo, Zapotec	Chinantec, Chontal de Oaxaca, Mazatec, Spanish, Zapotec
TD, TP	13,16, 17	3|11	Puebla	Cuetzalan, Sierra Norte de Puebla	Mestizo, Nahuatl, Totonac	Nahuatl, Spanish
AC, MB	09,16	4|3	Querétaro	Sierra Gorda	Mestizo	Spanish
MB, TD, TP	09,16	35|42	San Luis Potosí	Pamería; Huasteca	Mestizo, Nahuatl, Teenek, Xi’uiy	Nahua, Teenek, Spanish
AV	00	2|0	Sonora	Sierra Madre Occidental	Mestizo	Spanish
MB	09	16|4	Tamaulipas	Sierra de Tamaulipas; Ocampo Caves area	Mestizo	Spanish
MB	09	2|0	Veracruz	Huayacocotla	Nahua	Spanish
MB, TP	03–16	23|10	Honduras	Gualaco, Olancho	Mestizo, Nahoa de Honduras	Spanish
162|249 Total interviews 2000–2017						

^a^Interviewers

^b^Year or range of years during which interviews were carried out

^c^Number of interviews (exploratory|in-depth)

^d^All interviewers fluent in Spanish; TD fluent in Nahua; other interviews carried out through local interpreters

Drawing inspiration from Newsom’s summary of Caribbean ethnobotany [47], we supplemented ethnobotanical interview data with paleoethnobotanical and ethnohistorical data derived from literature reviews and communication with museums. For cycad paleoethnobotany, we sought to establish how long and in what regions and contexts cycads had been used. We subjected these scant data to minimal interpretation, and flag excavation methods, chronological uncertainty, and other factors that limit their utility. Our historical component sought to elucidate cycad ethnobotany from Spanish-Indigenous contact around 1500 through the late 1900s. We scanned full-text databases (e.g., Google Books) using common local names such as “teocintle” and “chamal,” and their variants, to locate non-indexed references. For these searches, we focused on works in geographic areas where the terms refer only to cycads and where the plant descriptions clearly indicated that cycads were being discussed.

The few published Colonial-era herbals were also examined, and certain unpublished archival materials were viewed. Except in Honduras, our archival research was quite partial, given the vast, mostly unindexed and un-scanned resources available in local and national repositories across MNCA and in Spain. The primary means we used to reconstruct post-1900 historical ethnobotany was via oral history questions that we incorporated in ethnographic interviews. We did not gather data on the commercial nursery trade, an important factor to consider for community-based conservation studied and quantified elsewhere, e.g., [48]. We released preliminary findings in public presentations, unpublished reports, e.g., [49], and a few popular [50] and scholarly [7, 28, 51] articles.

### Geographic scope

Though as a whole they are scattered across MNCA, cycads occur in limited areas and are completely absent from the Mexican central plateau and other arid highland areas.^1^ Given striking ethnobotanical similarities between Honduras and Mexico that originally sparked our curiosity and collaborations [22, 50–52], we included both countries as well as intervening Guatemala, El Salvador, and Belize where we were not able to undertake fieldwork. We came to focus on NEMX because of its accessibility to our research facilities combined with its rich existing cycad ethnobotany quite similar to that of Honduras. As our questions and hypotheses evolved, time and funds permitting, we visited other MNCA locations to broaden our scope and fill in gaps between NEMX and Honduras. Our efforts were far from exhaustive and an enormous amount of work remains to be done, including the integration of genetic evidence that helps test some of our hypotheses.

### Literature issues

Definitions and delineations of MNCA cycad species have varied based on differing interpretations of genetic and phenotypical characteristics. At present, full and recent taxonomic descriptions and revisions exist for many species, while monographs have been produced of the cycads for a few areas [31, 53, 54]. Sparse ethnobotanical data are found within botanical publications. Otherwise, secondary sources yield an uneven panorama of MNCA cycad ethnobotany (Additional file 1: Table S1 and S2). We took care to remedy incorrect species names and other confusions in the non-botanical literature, and uncertainties are noted (Additional file 1: Table S1).

### Ethnographic interviews in depth

We utilized maximum caution with interviewees to protect identities and knowledge. If free, prior, and informed consent (FPIC) was granted us, we asked local collaborators questions about cycads, and we also engaged in open-ended discussion. We focused our in-depth interviews on people that local authorities and other contacts deemed most knowledgeable about plants. In general, these were people over 50 years of age, though we also talked with younger people who were recognized as particularly knowledgeable. We did not thus focus on a representative cross-section of the population but rather on detecting the greatest amount of in-depth cycad knowledge in the time available to us, which repeated experience had taught us was closely associated with the age of the interviewee. In circumstances where we had limited time, we sought only the most elderly informants. We requested that local contacts and authorities provide us access to 50% men and 50% women. In the small, rural communities that we visited, our target was to be able to spend 2 or 3 days carrying out at least five in-depth interviews with women and five with men. This was not always possible, however, for a range of reasons, particularly including knowledge loss that resulted in less than ten total people having any in-depth knowledge whatsoever of cycads. Some communities only yielded exploratory interviews, or no data at all despite the presence of cycads.

See Additional file 2 for research guides and translations of Spanish-language question banks from which we drew. The extent and depth of questions grew over time, because when we began we had little idea of the dimensions of cycad knowledge and the answers that we would encounter, and virtually no prior studies to guide us. We selected questions for each community and circumstance based on our background knowledge of socioeconomic conditions, cultural and political sensitivities, and other contextual issues including any concerns that arose during the process of garnering FPIC. Therefore, in general, two sample strategies were applied: first, open interviews and then, semi-structured interviews.

Prior to asking exploratory questions, we always sought to determine if the potential interviewee was familiar with any of the local cycads we thought should be present according to published botanical studies, examination of herbarium vouchers, and personal communication with botanists. Ethnobotany research carried out in Hidalgo and San Luís Potosí was preceded by detailed population biological studies of all cycads species present [55, 56]. We confirmed the botanical identity and ethnobotany information with the aid of AV, who is the national expert in Zamiaceae in Mexico. The botanical vouchers are available at HGOM and XAL herbaria [55, 56]. We typically evoked a well-known local name and showed a cycad leaf or other part, a herbarium voucher, or a color photo. We also mixed in photos of similar local plants to establish whether or not the potential interviewee was actually talking about cycads. In many cases, cycads are confused with palms, ferns, pine trees, and other plants, particularly in regions where substantial knowledge erosion has taken place. If possible, we had the potential interviewee show us what they thought was a cycad plant of their own volition—i.e., in a nearby woods or their garden. In addition, given the extensive work that has been done by biologists, it was usually easy for us to identify local cycad species, which are typically limited to a handful in any given locale, with little potential for confusion. Any uncertainties are flagged (Additional file 1: Table S1).

If we established that the potential interviewee was familiar with cycads, then with their permission, an open, exploratory interview usually commenced, focusing on its names, and if these were in an Indigenous language, we asked the interviewee or a family member, guide, or interpreter to write them phonetically. Names were also recorded on audio, if possible. Whether or not names were known, we asked whether any uses were known. Depending on the nature of the responses, open exploratory interviews lasted from 5 to 30 min, and if conditions were amenable, these became in-depth, semi-structured interviews. In many cases, however, in-depth interviews were left for a later time, to allow the interviewee to collect their thoughts. Where very little was known, we asked the interviewee (or they responded unsolicited) who would possibly know more, thus a snowball sampling method of potential interviewees was used in some communities. In Teenek, Nahua, and certain other Indigenous communities, where we apprised community leaders of the nature of the research, they met to decide whether to grant FPIC, and if they did, thence accompanied us or let us go solo or with an interpreter to interview our target of ten pre-designated elderly cycad experts. Each community was quite distinct in how it approached our research, and in some regions and contexts, particularly in non-Indigenous areas, it was not necessary to do more than inform local leaders of our presence and purpose and show letters of introduction from our research institutions. In some cases, local leaders evinced marginal or no interest in what we were doing and told us there was no need to garner their permission; in such contexts, we sought FPIC person by person.

In-depth interviews lasted 30 min to 2 h, over one or more sessions. Depending on FPIC strictures, we recorded interviews via written notes, audio, and/or video. Interviewees’ family members and friends often stood by, sometimes engaging in discussions and debates about cycad knowledge. Interpreters were utilized in the case of interviewees who did not or preferred not to speak in Spanish. Interviews took place at the time and place of the interviewees’ choosing, typically in their homes and gardens. We gave sufficient opportunity for interviewees to provide as much detail as they wished.

Research protocols followed the authors’ Institutional Review Boards’ and professional ethical guidelines and requirements [57] and though our studies were exempted from necessity to provide human subject protections, we protected interviewee identities and sometimes community names and locations nonetheless (no names or other personal identifiers have been retained). In Honduras, this was due to risk of human rights abuses of interviewees and collaborators by government authorities and other actors, such as those that followed the 2009 *coup d*’*état* [7]. In Mexico, though we did not find this to be the case, we opted to hide identities and localities to impede plant traffickers who access published data to find informants who can point them to cycad populations. This is a serious problem in Mexico, a result of the extremely high value of cycads for domestic and foreign decorative use and is why exact geographical coordinates of population locations for most cycads should not be revealed in publications [4, 5].

As an additional precaution, we carefully explained to collaborators and authorities, whom we assumed were unaware, that cycads are sometimes utilized for secret and even illicit purposes connected to ethnomedicine and narcotics [58]. Thus, they could tell potential interviewees to be wary of what knowledge they chose to divulge, and explain that any knowledge divulged inadvertently could, upon an interviewee’s or community’s request, be destroyed and/or remain unpublished at any point ex post facto. Though we were never asked to destroy our data, in several cases potential interviewees—typically traditional healers and shamans—whom local authorities had identified and recommended to us did not wish to be interviewed once they learned the exact nature of our research.

### Participant observation

In NEMX and Honduras, we stayed in local homes over the course of days and in some cases weeks, affording us the opportunity to gain trust. Where permitted, we visited and documented local cycad populations. Where possible, we participated in or observed cycad food preparation and consumption as well as religious events. In addition, we searched local landscapes, particularly graveyards, for iconographic representations and decorative uses. Because religious monuments appeared to contain representations of cycads, even where cycads were no longer used or the images were popularly identified as other plants in local tourist literature, we often documented façades and other components of religious monuments.

## Results

Unless specifically noted otherwise, ethnographic and ethnohistorical data we summarize in this section are derived from “Ethnobotany of MNCA cycads by local term” (Additional file 1: Table S1), which contains data from our own field observations as well as references to the literature and is organized alphabetically by local cycad term; columns detail local terms and their translations, language, ethnic group, species, locations, and uses/knowledges in the categories of alimentary, economic, agroecological, religious, and “other.” These data are rearranged and tabulated by species in “Ethnobotany of MNCA cycads by species” (Additional file 1: Table S2) for major uses and beliefs (alimentary, maize association, economic, agroecological, religious, medicinal, decorative non-religious) and minor uses and beliefs (as toys, sense of place, weather, assassination, construction, narcotic, entheogen, dye/coloring agent, combs, weaving, laundry starch, and envelope glue).

### Nomenclature by ethnic group and language

We identified 235 unique terms (“Local Term” column, Additional file 1: Table S1), some with variants, used to refer to cycads and environments, processes, and events involving cycads. While some terms are generic and are applied widely to other plants, others are specific to cycads. We recorded locally used terms in 17 Indigenous languages as well as in Spanish and English. Several terms combine roots from two languages. More ethnic groups (27 Indigenous, in addition to non-Indigenous *mestizos*) have terms for cycads than are reflected in languages, because some groups no longer retain Indigenous languages, while other groups only retain names in Spanish or English.

Several ethnic groups visited—e.g., Tepehua (in Hidalgo state)—and numerous mestizo communities where cycads occurred locally did not have names for cycads. In some cases, this may have been due to insufficient fieldwork on our part, but in many instances, it was the fault of eroded cultural knowledge as well as non-transmission of knowledge between earlier inhabitants and in-migrants who had mixed with or supplanted them. In many instances, cycads were known only as palm trees (*palmas*, *palmitas*, etc.) or only by names collectors had left behind (e.g., *cica*). We excluded non-local names, such as those used in nurseries, public parks, and arboretums, where these are either inventions for commercial purposes, or general names such as *zamiáceas*, *cícadas*, and *cicadáceas*.

### Regional summary

Intra-regional commonalities in cycad usage and knowledge are detectable within five NMCA regions, based on geographic proximity and shared cultural histories.

In Western Mexico (WMX) (Guerrero, Colima, Michoacán, Jalisco, Nayarit, Durango, Sinaloa, and Sonora states), *Zamia paucijuga*, *Dioon stevensonii*, *Dioon tomasellii*, and *Dioon sonorense* are found in isolated populations scattered across the vast Sierra Madre Occidental from sea level to over 2000 m. Scant ethnobotanical data are available. No Spanish or Indigenous terms or knowledge relate WMX cycads directly to any current or prior food uses, though two common NEMX food-related terms for cycads—*chamal* and *teosintle*–used as recently as the 1980s in Jalisco suggest potential former food uses. Cycads are thought of as types of palms and utilized in Roman Catholic religious ceremonies syncretized with Indigenous religions, such as among the Cora in Jesus María and surrounding Nayarit communities.

NEMX (SE Nuevo León, Tamaulipas, E Guanajuato, NE Querétaro, San Luis Potosí, Hidalgo, N Veracruz, and N Puebla states) includes many subranges of the Sierra Madre Oriental as well as Gulf of Mexico coastal lowlands. It is a rich area for cycad diversity and endemism, with several varieties of the *Dioon edule*/*angustifolium* group, often in large populations, as well as numerous *Ceratozamia* and a few *Zamia* spp. Most Mexican archeological and historical ethnobotanical records of cycads derive from here, related to the fact that the highly visible *chamal* (*Dioon edule*/*angustifolium*) once covered vast areas of mountains and lowlands, and was a staple food for the Xi’iuy Indigenous group as well as for non-Indigenous mestizos. In addition, *Ceratozamia* spp. broadly called *tzompoyo*, *teocintle*, or *chamalillo*, and *Zamia* spp. were also widely consumed for food, and many species are still featured in religious ceremonies, medicines, and other uses. Particularly fine-grained knowledge about cycads we have recorded in MNCA is found among the Nahuas of N Hidalgo and SE San Luis Potosí, the Teenek of SE SLP, the Xi’iuy (“southern Pame”) of SLP and Querétaro, and the mestizos of the Sierra Gorda in Querétaro and their descendants elsewhere. Southward, food uses are mostly unknown to mestizo, Nahua, and Totonac peoples (e.g., in the Sierra Norte de Puebla). Across the entire region, cycads are often conceptualized as types of maize, friends of maize, and ancestors of maize.

The Southern Gulf of Mexico Coast and Yucatán peninsula region (southern Veracruz, Tabasco, Quintana Roo, Yucatán, and Campeche states) contains *Zamia* spp. throughout. Our preliminary researches and inquiries suggest cycads are of minor importance to local Indigenous groups (primarily Maya language group speakers). They are occasionally made into breads and medicines.

In Southern Mexico (SMX) (Oaxaca, SE Puebla, and Chiapas), an extraordinarily diverse set of cycad species exists, with concomitantly rich and poorly known ethnobotany; there are multitudinous alimentary and religious uses and meanings and the plants have been used by people since at least 6000 BP. Belief in cycads as maize ancestors is found here among the Chontal de Oaxaca, and alimentary uses are still common among Chinantec speakers. Numerous ceremonial and medicinal uses exist. Elaborate pilgrimages involving carrying leaf bundles from sacred *Dioon merolae* populations to local altars characterize Indigenous cultures in the Isthmus of Tehuantepec and nearby regions of both Chiapas and Oaxaca.

In northern Central America, sparse data for Guatemala and Belize indicate potential connections to maize, and an overall association with *camotillo* poison. For El Salvador, we found virtually no ethnobotanical data on *Zamia herrerae*, the single species recorded there. Honduras’s six species are associated with a wide range of beliefs and uses discussed in this article, including for food, poison, and religious ceremony. Most notable are the similarities of *tiusinte* (*Dioon mejiae*) culture to NEMX cycad culture.

### Paleoethnobotanical evidence for six millennia of continuous use

Most data on macrobotanical remains of cycads from rock shelters excavated by MacNeish [12] and analyzed by Smith [19] have been previously mentioned [59, 60], but not within the context of cycad studies. Beyond MacNeish’s findings, the sole reference we encountered was to *Zamia* pollen at the Classic period Escalera del Cielo site in the Puuc Maya region, Yucatan [61]. We present the first comprehensive compilation of these data in one place in Table 2.

**Table 2:** Cycads in the Mexican archeological record, 4700 BC–1780 AD

Region Site • Phase (dates)	Cycad remains recovered and relationships to other useful plants						
SIERRA DE TAMAULIPAS [12]	Total food recovered (L)	Dom. plants (L)	*Dioon* (L)	Maize (L)	Wild plant food (% of diet)	Animal food (% of diet)	Dom. plants (% of diet)
* Cueva de la Perra* ^a^							
• La Perra (3000–2200 BC)^b^	19.77	1.84	8.1 (41%)	0.85	76	15	9
• Laguna (600 BC–0 AD)^b^	23.72	9.28	3.09 (13%)	5.91	51	9	40
* Cueva del Armadillo*							
• Los Angeles (1200–1780 AD)^b^	15.57	6.17	3.44 (22%)	5.42	42	18	40
SIERRA MADRE DE TAMAULIPAS							
* Ojo de Agua cave*^c^							
• Palmillas (260–960 AD)	*Dioon angustifolium*: 89 “leaf bases,” 20 “bracts,” 9 seeds						
* Romero’s Cave*^d^							
• Occupation # 14 (1100–1500 AD)	*Dioon angustifolium*: 2 sclerotesta fragments, with *Lagenaria* and *Cucurbita* remains (unexamined remains may include other *Dioon* fragments)						
VALLE DE TEHUACÁN [19]	“The broken seed coats [sclerotestas] of this plant are not abundant, but showed that the plant [*Dioon* sp.] had been fairly persistently used [in the Tehuacán Valley] over some thousands of years”; “The oblong seed...provides a starch food when cooked, but it could never have been important in the diet of the Coxcatlán Cave people”^e^						
* Coxcatlán Cave*	Seeds recovered		Seeds present^f^		Food (L)^g^		
• Coxcatlán XII (4700–4300 BC)	1		12		0.1		
• Coxcatlán XI (4217–4025 BC)	7		56		0.3		
• Abejas X (3300–3100 BC)	1		30		0.1		
• Abejas IX (no dates specified)	1		5				
• Santa Maria VII (450–100 BC)	13		26		0.1		
• Palo Blanco VI (150 BC-280 AD)	4		4				
• Venta Salada III (790–1010 AD)	2		2				
• Venta Salada II (1000–1178 AD)	2		2				
* El Riego Cave* ^h^							
• Palo Blanco (700–900 AD)	1 sclerotesta fragment						
PUUC MAYA REGION, YUCATÁN							
* Escalera al Cielo*^i^							
• Terminal Classic (800–950 AD)	3 *Zamia* sp. starch grains on 3 hand-held stone grinding implements						

^a^The dominant plant remains of the [La Perra] horizon are *Dioon edule* [*D*. *angustifolium*]. There were 14 bases, 31 bracts, and 1621 seeds of this plant. Since 200 of these acornlike seeds fill a liter, 8.10 liters occur ([12], pç 144)

^b^“[T]entative absolute dates” ([12], p. 198, Table 31)

^c^Zone C, Occupation 10 ([101], cited in [59]). (*Dioon* remains also found in Valenzuela’s and Romero’s caves ([102, 103], according to [59]; see also below))

^d^Data provided by Illinois State Museum (emails from Dee Ann Watt to Mark Bonta, 11/17/2009 to 11/30/2009, including photographic plate of *Dioon* remains); catalog # 817/541ai

^e^([19], p .235)

^f^Adjusted for “preservation factor”

^g^Estimate of food available based on preservation factor

^h^Four kilometers W of Coxcatlán Cave. From “east niche...zone d”

^i^From Table 7.1, “Starch grains and phytoliths recovered from handheld grinding implements (sediments 2 and 3)” ([61], p. 262)

MacNeish documented *Dioon* in at least six rock shelters during his long search for maize origins, which began in the Sierra de Tamaulipas in the 1940s, moving southward to the Sierra Madre de Tamaulipas and eventually the Valle de Tehuacán [59, 62]. His richest and most significant cycad find was in the Cueva de la Perra (“La Perra”) rock shelter in the isolated Sierra de Tamaulipas. *Dioon* remains were found as a majority of all edible plant remains beginning from the earliest macrobotanical evidences at around 4000–4500 BP. Stresser-Péan inferred from the abundance of cycad remains that *Dioon* had been a staple food for mestizos and Indigenous people in the Sierra since that time [40]. MacNeish, who characterized the cycads as “poisonous nuts” [12], afforded their overwhelming quantity in La Perra only limited importance; in the same strata as the cycad remains, tiny maize stole the show as the earliest evidence of domesticated maize recorded at the time. But with maize only appearing in very small quantities, MacNeish concluded that La Perra had functioned as a seasonal wild plant foraging camp. Given that large *chamal* populations still exist throughout the Sierra, some quite close to La Perra, we suggest that the shelter was specifically a seasonal cycad harvesting encampment, explaining the overwhelming presence of this plant’s remains in the cave. La Perra’s function may have been similar to the cycad harvesting camps of recent times we have documented in NEMX and Honduras (see below). In the Sierra de Tamaulipas, cycad harvesting culture lasted to the 1700s AD when local indigenous groups were exterminated. Given cycad longevity, it is likely that the same populations and possibly some of the same plants were harvested continuously over this time frame.

A question unanswerable until lithic or ceramic implements at La Perra are analyzed is whether or not the toxic starches of cycad seeds were processed for food, or whether the apparently innocuous sarcotestas alone were used (they still are processed into tortillas in other parts of NEMX at present, e.g., [63]; Additional file 1: Table S1) which would have required no specialized knowledge of toxin extraction by water leaching, sun-drying, boiling, or other methods.

In the Valle de Tehuacán, *Dioon* remains were found sparsely throughout the archaeobotanical record beginning around 6000 BP. Purportedly small *Dioon* populations near the rock shelters would not have allowed them to occupy dietary roles as prominent as those in the Sierra de Tamaulipas [19] but the remains are important nevertheless as they show yet another area where cycads and early maize existed in the same cultural context.^2^

### Historical evidence for food use and religious significance

While European chroniclers often recorded *Zamia* spp. as staple foods, and as early as the 1500s, in the Caribbean, e.g*.*, [21], in Mexico, sixteenth-century chroniclers were based in the central volcanic highlands from which cycads are absent, and perhaps consequently, few unequivocal references to cycads from that period have come to light. The voluminous Florentine Codex [64], an ethnography relying largely on Nahua informants from central Mexico, contains no obvious references to cycads, and only a few vague potential references, e.g., to the plant known as *xioactli*; the Cruz-Badiano herbal contains no clear references to cycads, either [65]. Francisco Hernandez’s botanical *magnum opus* [66] contains references to *teocintli* and *tepecintli* (*Ceratozamia fuscoviridis*) from Huayacocotla, a Nahua town in the Sierra Madre Oriental, Veracruz, where it is still known by these names and was still used as food as recently as the mid-twentieth century (see Additional file 1: Table S1).^3^

All other written historical ethnobotanical data we have found for Mexico refer to the *chamal* cycads [67, 68]. From the 1600s onward, they were mentioned as important foods for indigenous people and starvation fare for non-indigenous people in NEMX. Reflecting their historic visibility in the landscape, numerous toponyms, some dating from colonial times, if not earlier, incorporate “*chamal*.” The species referred to are the very similar *Dioon edule* and *D*. *angustifolium*.

**Fig. 2:**
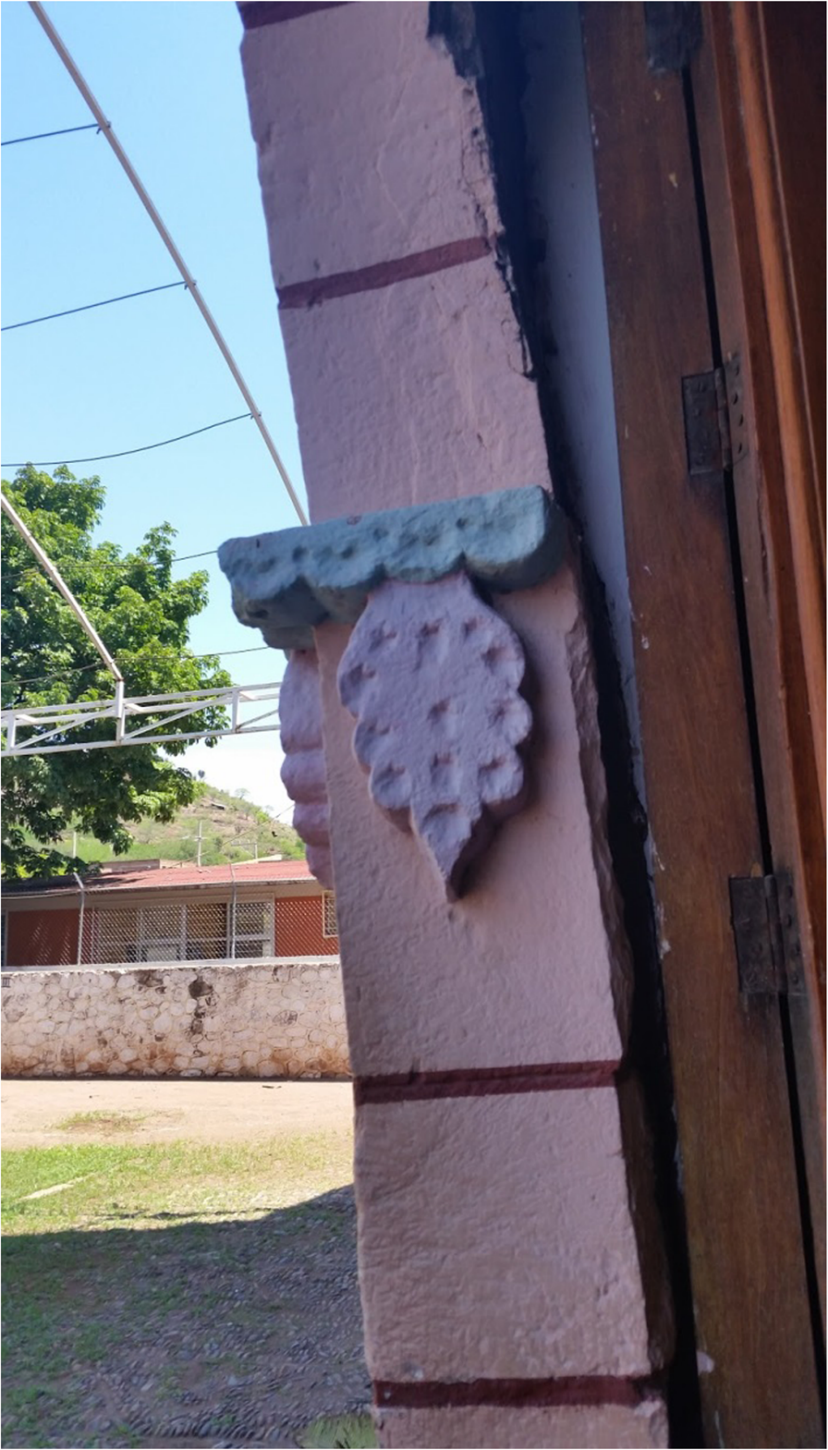
Plaster *turhaa* (*Dioon tomasellii*) cones carved on colonial church entranceway in Jesús María, Nayarit, 2016. Cora people utilize *turhaa* leaves from wild populations to decorate churches and churchyards in the Sierra del Nayar region during Holy Week (Semana Santa)

**Fig. 3:**
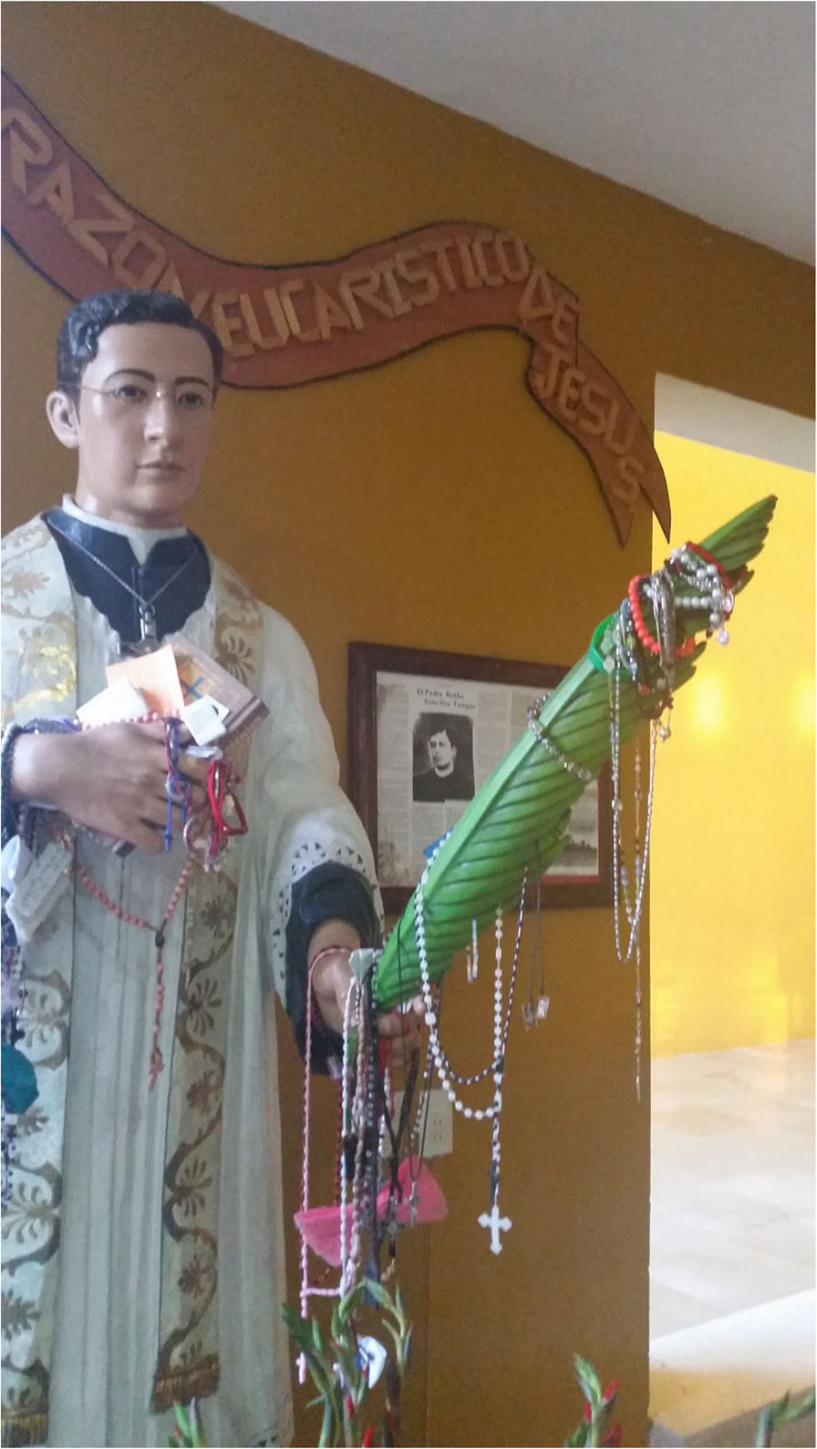
San José María Robles Hurtado, a twentieth-century Roman Catholic saint associated with the Guerra Cristera war, holds a carved *palma de la Virgen* (*Dioon tomasellii*) leaf. Quila shrine, Tecolotlán, Jalisco, 2016

**Fig. 4:**
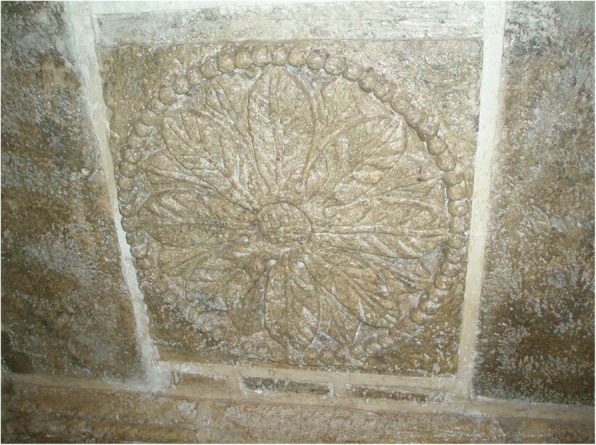
*Teocentli* (*tepecintle*, *Ceratozamia fuscoviridis*) leaf crown and cone on outside wall of colonial church, Huayacocotla, Veracruz, 2009

**Fig. 5:**
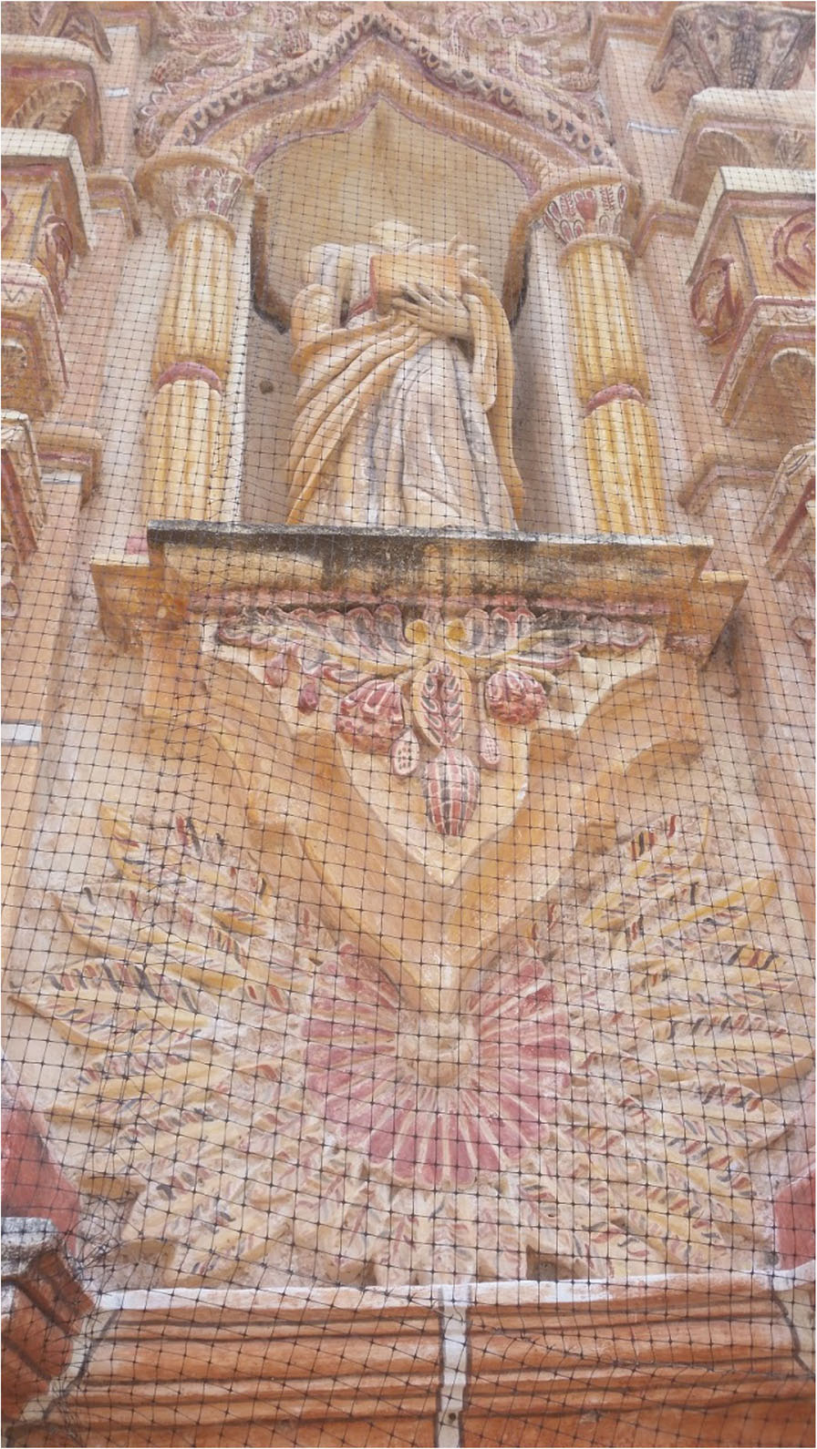
Restored plaster, Baroque facade of the late eighteenth century Tancoyol mission church, Jalpan, Querétaro, likely fashioned originally by Xi’iuy artisans. The numerous apparent cones and leaves of *dameu* (*Dioon edule*), a staple Xi’iuy food and sacred plant, have been misinterpreted as maize, or as *Acrocomia* palms due to a mistranslation of “Tancoyol” (“coyol” is the *Acrocomia* palm in Nahua); the toponym combines two Teenek words: *tan*, place of, and *coyol*, either “guan,” a Galliform bird, or a variation of *coxol*, “mosquito”

**Fig. 6:**
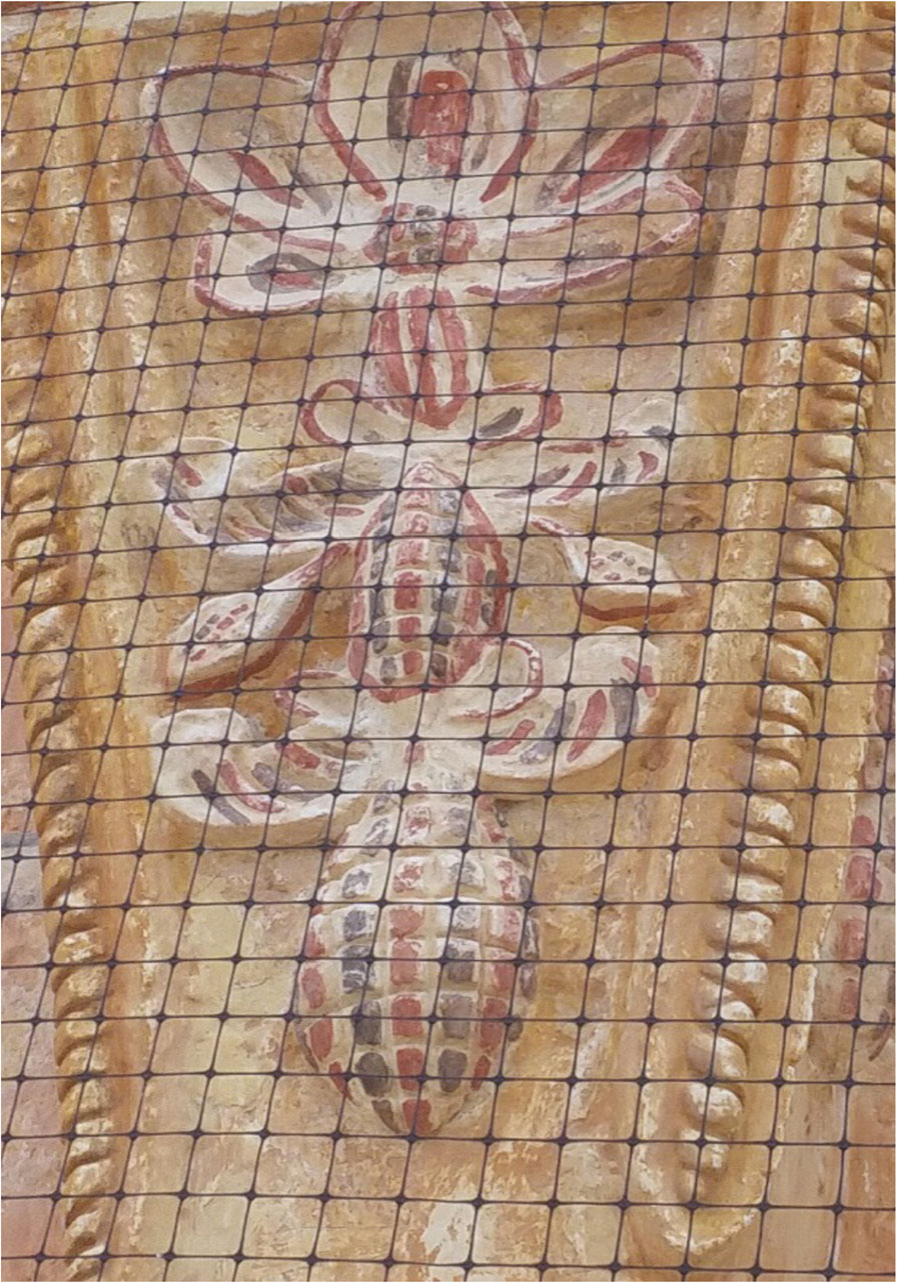
Close-up of *dameu* (*Dioon edule*) cones and leaves in Tancoyol, 2016

**Fig. 7:**
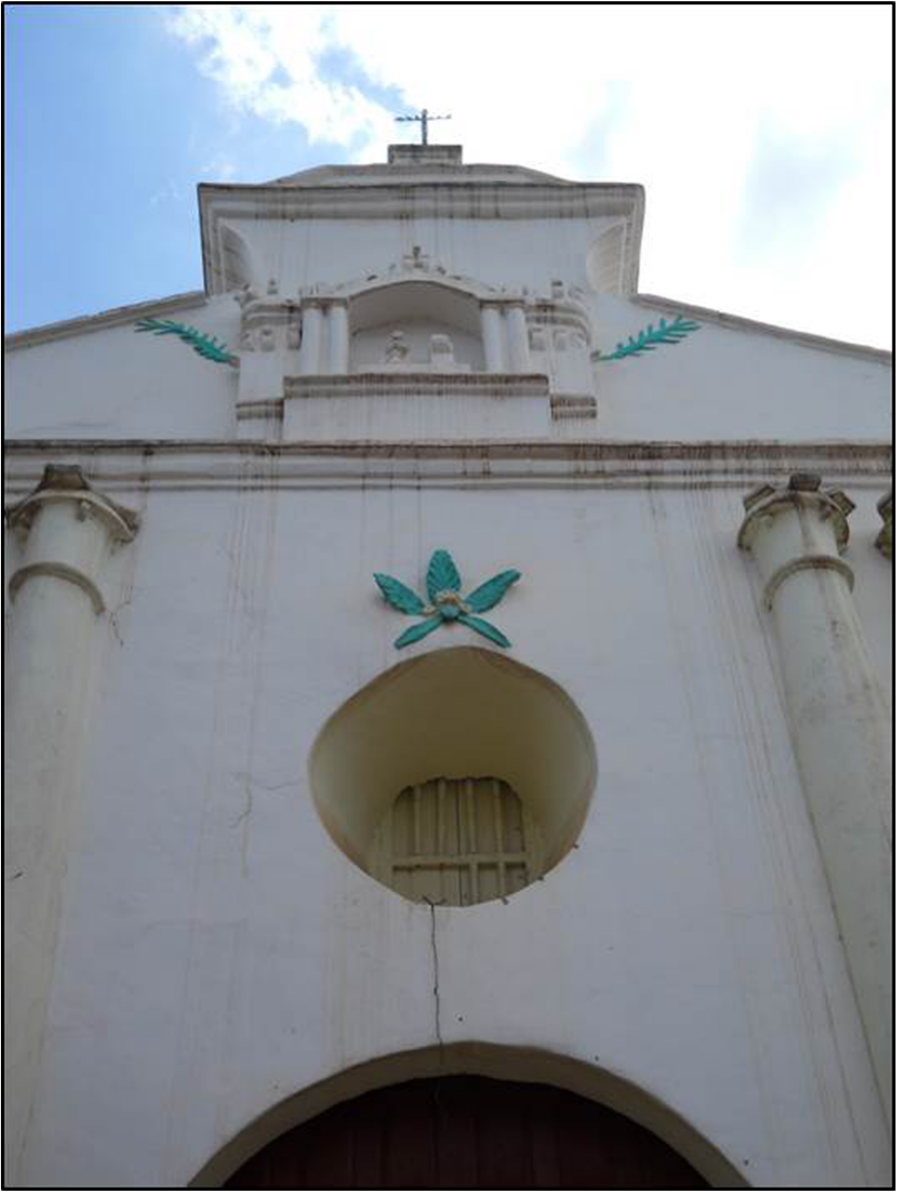
*Tiusinte* (*Dioon mejiae*) carving on restored façade of eighteenth-century church, Gualaco, Honduras, 2010

Cycads appear to be incorporated into the iconographic representations on façades and other decorative elements in MNCA Catholic churches and shrines dating from the 1500s to the 1900s. We have putatively identified such representations in Jesus María (Nayarit), Tecolotlán (Jalisco), Huayacocotla (Veracruz), San Antonio Tancoyol (Querétaro), and Gualaco (Honduras) (Figs. 2, 3, 4, 5, 6, and 7). There (see *turhaa*, *palma de la Virgen*, *teocentli*, *dameu*, and *tiusinte* in Additional file 1: Table S1), cycads have played important roles in religious ceremonies, and in the latter two places, *dameu* and *tiusinte*, have also been critical dietary staples. While differing interpretations may suggest the plants portrayed are maize or palms, we feel there is a strong case for cycads, given that local cultures in places like Gualaco and Querétaro even today elevate them above maize in status, importance, and ritual.

While we have examined but a very small portion of archival and published accounts potentially containing cycad references in Mexico, in Honduras we have studied all available land titles and other documents across the remote northeastern part of the country where culturally significant *tiusinte* cycads have been found [51, 52, 69]. Unequivocal mentions of *Dioon mejiae* (as *teocinte* and spelling variants) begin to appear in the first surviving land titles (1700s) and are also included in natural resource inventories and agricultural censuses of the 1800s and 1900s, as well as local newspaper accounts, travelers’ journals, and other sources. Extrapolating from our exhaustive Honduran research, we think it safe to assume fine-grained archival research elsewhere in MNCA where cycads have been prominent as staple foods and in religious contexts will yield similar results.

We gathered oral historical accounts from Honduras, Oaxaca, and NEMX, and scattered records from elsewhere (marked as “formerly” in Additional file 1: Table S1). Where cycads are still eaten and appreciated, oral histories typically recalled richer, more nuanced traditions and knowledge than those that persist today. Where cycads are no longer eaten but are still utilized for other purposes, particularly in Zapotec and Teenek contexts, cycad consumption often evoked distant shameful memories of dire poverty and lack of maize among elderly first-language speakers. Teenek interviewees who as children had been forced to gather and eat cycad seeds during famines in some instances terminated interviews with us or cried over the long-buried memories of the suffering associated with the practice. Though such reactions point to cycads as non-desirable “famine foods” rather than desirable staples for such cultures, we found simultaneously that adjacent ethnic groups did not feel this way at all. For example, mestizos and Xi’iuy people who continue to harvest from the same cycad populations that Teenek informants were forced to harvest from in the 1940s perceive cycad foods as objects of pride and embodiments of local identity, with highly desirable tastes and textures: i.e., in exactly the opposite manner as the Teenek. The same can be said in Oaxaca, where Chinantec and to a lesser extent Mazatec speakers consume and value cycad foods at present, Chontal de Oaxaca value them as a maize food of the ancestors, while Zapotec people value them for religious reasons but excoriated them (in communities we visited) as famine foods.

Across all MNCA cycad-eating regions, consumption has diminished considerably over the past century as modern transportation has brought communities closer to market centers and maize has become regularly and predictably available for purchase. In the post-World War II period, cycads and numerous other wild foods were all but abandoned. Even in areas where strong connections between cycads and maize continue to exist and cycad eating is not stigmatized (e.g., Nahua of N Hidalgo), consumption largely disappeared during the mid-twentieth century.

### Strategies of contemporary food processing

**Fig. 8:**
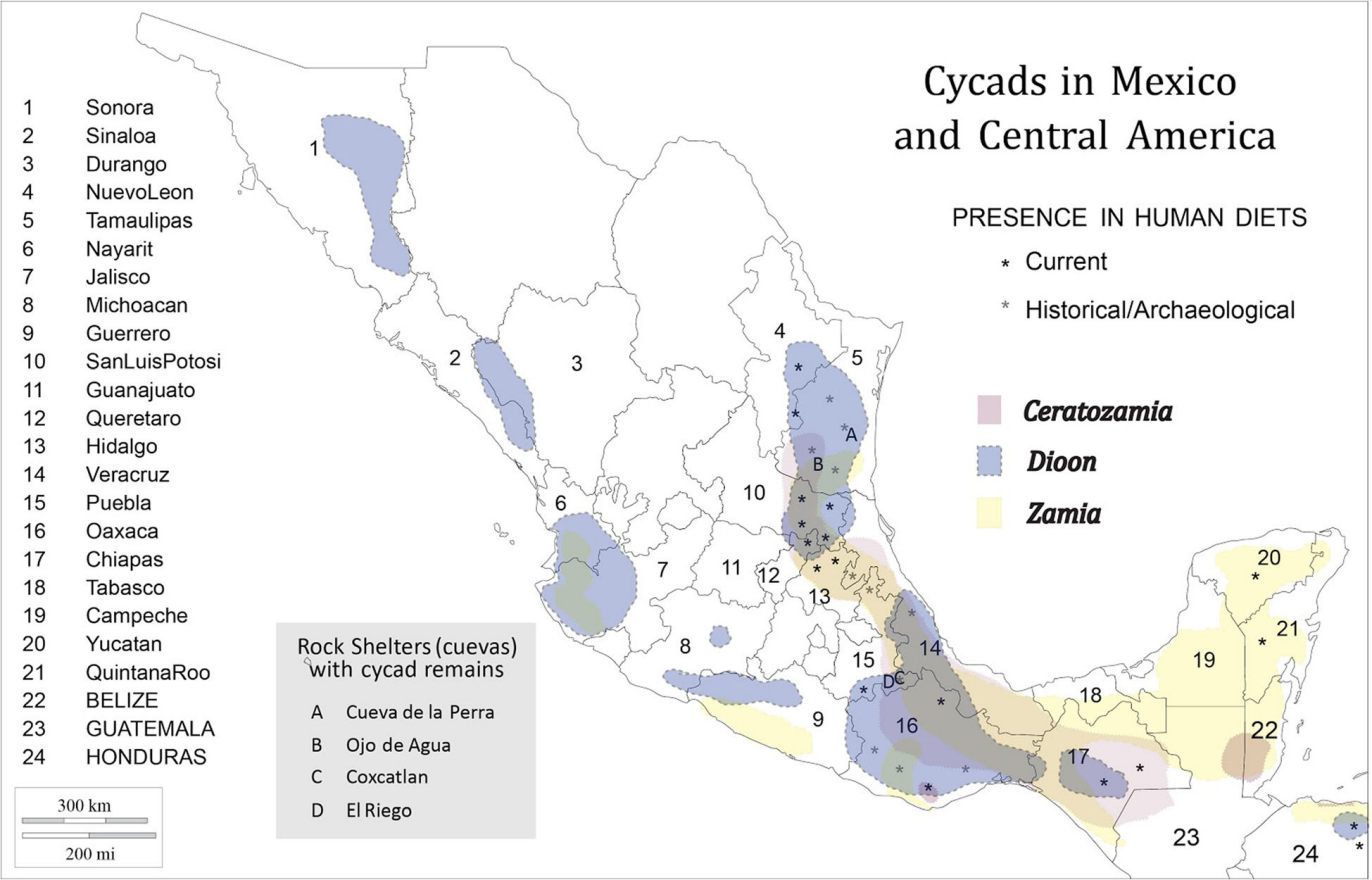
Presence of Mexican and Central American cycads in human diets

Cycad food and drink preparation processes have been documented in detail from several countries outside the region (Fig. 8) (e.g., Dominican Republic [70], Colombia [20], Australia [25, 27], the USA (Florida) [71]), but few non-Australian studies discuss components of harvesting, preparation, and consumption in any detail. Our data (see Additional file 1: Table S1) reflect diverse food strategies for species used by mestizos, Xi’iuy, Nahua (formerly), and Teenek (mostly formerly) in NEMX, as well as mestizos, Nahoa, and Tolupan of Honduras. Additional accounts from groups such as Chinantec, Mazatec, and Yucatec Maya lack detail but do underscore the past and in some cases continuing consumption of cycads in SMX and the Yucatán Peninsula. In total, we have confirmed that 15 species are currently consumed (9 *Ceratozamia*, 5 *Dioon*, 1 Zamia) and 5 more were consumed in the past (1 *Ceratozamia*, 3 *Dioon*, 1 *Zamia*), while we have strong evidence for the consumption of another 7 species (6 *Ceratozamia*, 1 *Dioon*) (see “Alimentary” columns, Additional file 1: Table S1 and Table S2).

Mestizo practices have been derived in large part from Indigenous practices via knowledge transmission within mixed Indigenous-mestizo families and communities. However, non-Indigenous ingredients of European derivation are also incorporated into mestizo recipes, particularly in Honduras.

As discussed below, starch detoxification is centrally important to cycad consumption. However, as noted above, the sweet sarcotesta is also eaten. It is consumed as a raw snack or in preserves (jellies) in Mexico, a practice unknown in Honduras. The consumption of tortillas made from a flour ground from dried sarcotestas has been recorded in recent times from SE Nuevo León state [86] and in a few places elsewhere in Mexico.

**Fig. 9:**
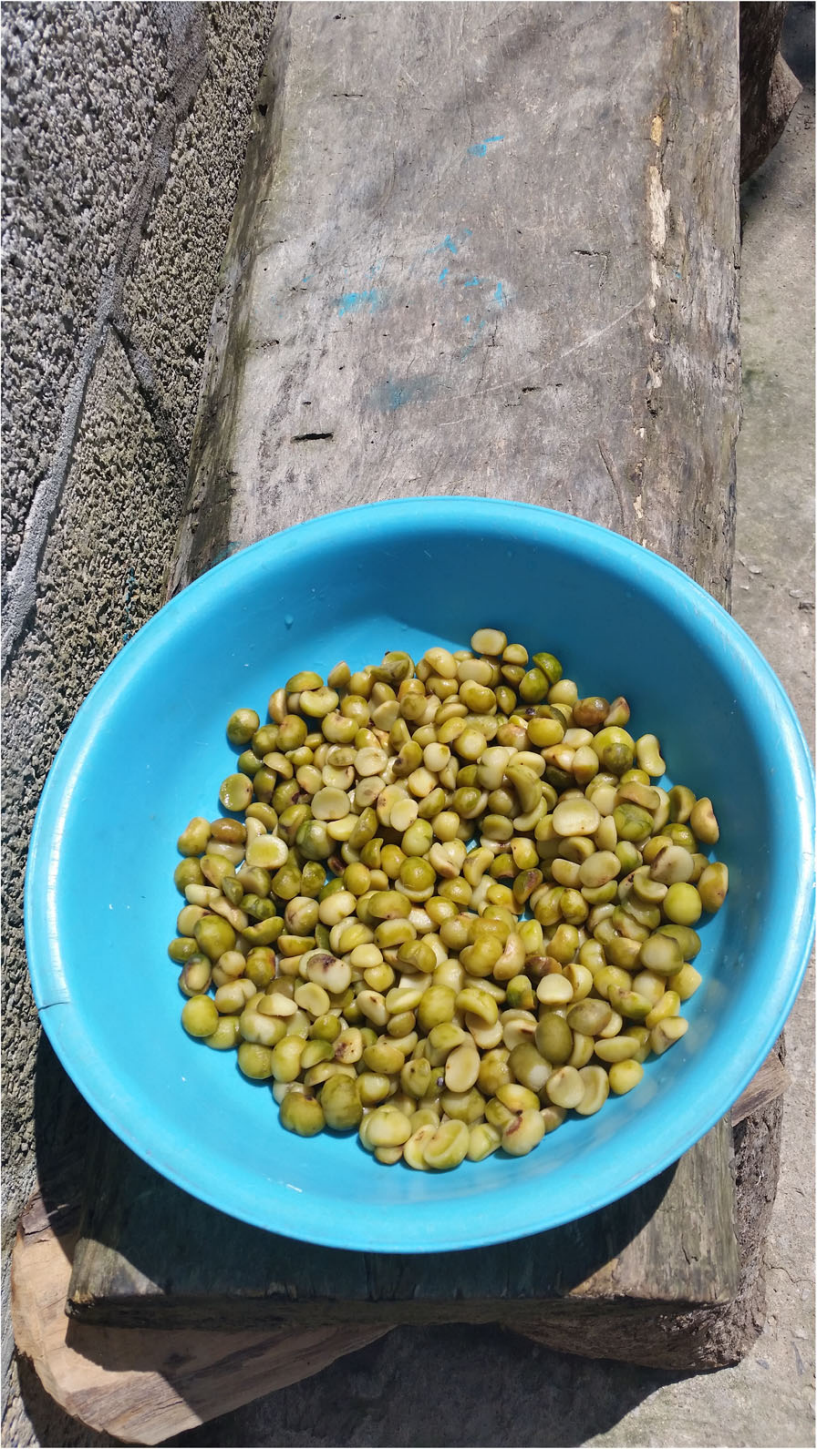
*Konlif* (*Ceratozamia latifolia*) seeds ready for detoxifying and *nixtamalización* to obtain wet meal for tortillas, Octujub, San Luis Potosí, 2016. This nearly vanished food is still prepared by a few of the most impoverished residents of the Sierra Teenek region

The seeds of all female cycad cones contain carbohydrate-rich starches. *Dioon* populations with hundreds or thousands of coning females are capable of sustaining many families in a given year, particularly helping tide them over during the months when the previous year’s maize stores run out (typically March–June or later), and before the new maize crop is available (in almost all cycad areas in the region, maize is the principal staple). *Ceratozamia* populations with enough cones also make labor-intensive harvesting, processing, and consumption worthwhile (Fig. 9), a practice persisting among highly impoverished families in NEMX, as well as in Chiapas. In general, *Zamia* starch, very rarely still used for food in MNCA, is procured from stems rather than seeds.^4^

Extraction of stem (*tronco* and *raíz*) starch is the other means of procuring cycad food in MNCA, but given the slow growth rate of many cycads, the practice is highly unsustainable and a reason for historical destruction of cycad populations during famines. Additionally, in Sonora, extraction of *Dioon* stem starch for use in *sotol* alcohol resulted in the complete destruction of populations. In Honduras, we did not uncover accounts of stem starch use.

In NEMX and Honduras, detoxification methods for seed starch favor the use of ash (*ceniza*) from a variety of trees [52, 72, 73], rather than lime (*cal*). In these two regions, except among the Tolupan and some Teenek, detoxification with ash is regarded as producing flour or meal superior to that produced using lime. Nowhere else did we find evidence of ash ever being used, lime predominating instead. Across the region, other methods, including boiling, baking on coals, and leaching in water and sunlight, are occasionally employed as well.

The critical moment in cycad starch detoxification is the point when the cook applies a test to determine that the starch is toxin-free. Various tests are applied, some involving the reactions of maize grains to the ash- or lime-infused cycad water that may have already been changed five or more times over the 2-day cycles of seed washing, steeping, and boiling [72]. Accidental poisonings, when they do occur, are said to result from the overly famished and or insufficiently knowledgeable cooks’ impatience. Food derived from incompletely detoxified cycad starch produces effects ranging from stomachaches, nausea and vomiting to death, the latter mentioned as the result of *Zamia* stem consumption in a NEMX Nahua community.

**Fig. 10:**
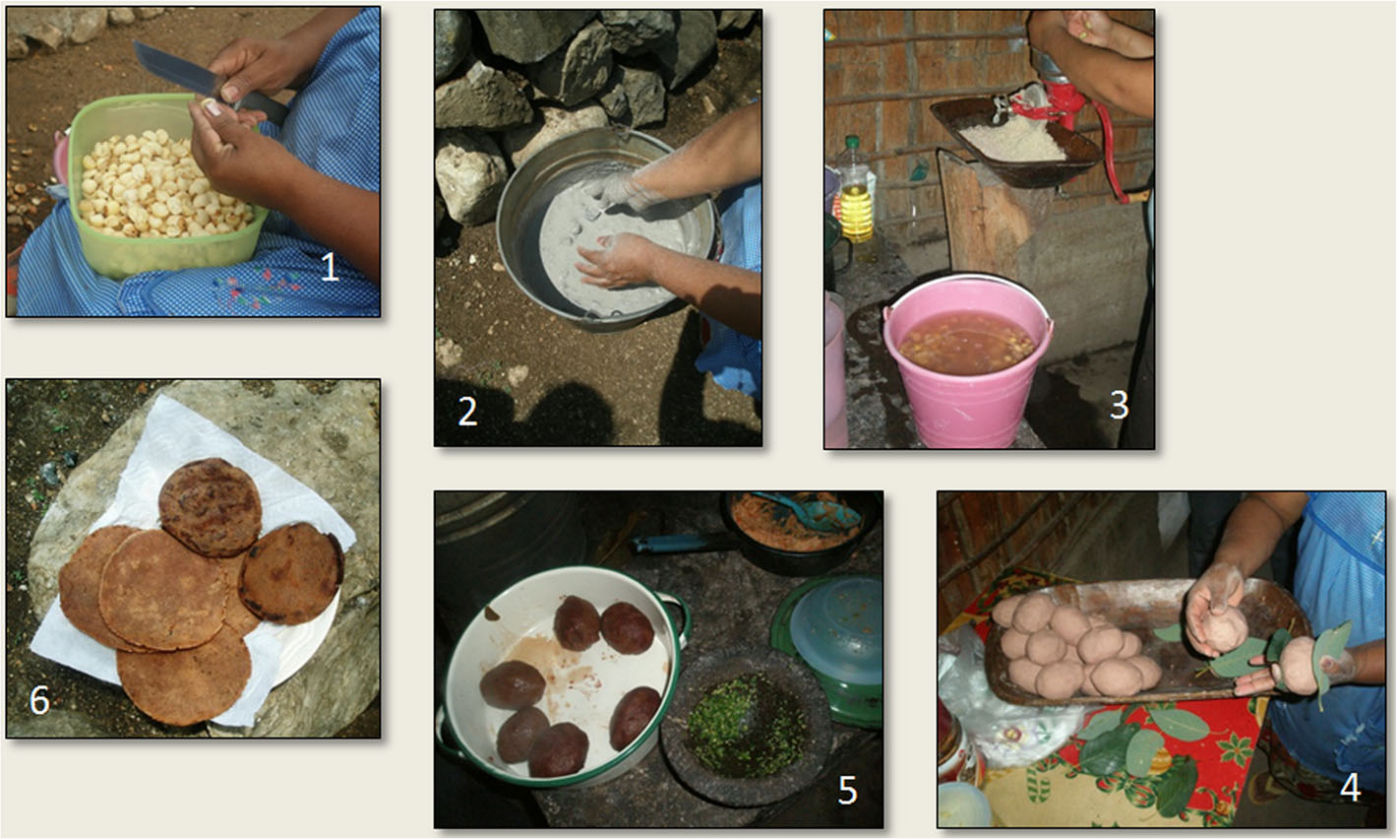
*Tamales de chamal* preparation process, Pamería region, San Luis Potosí, 2009. 1: Seeds, with sarcotestas removed, are cut into chunks. 2: Seeds are washed in ash and then boiled, and this is repeated several times. 3: Detoxified seeds are ground into *masa* (meal). 4: Tamales are shaped from the *masa*, sandwiched between leaves, and packed into a pot for cooking. 5: Finished tamales take on a ruddy hue and rubbery texture. 6: Tortillas are also fashioned from the *masa*

**Fig. 11:**
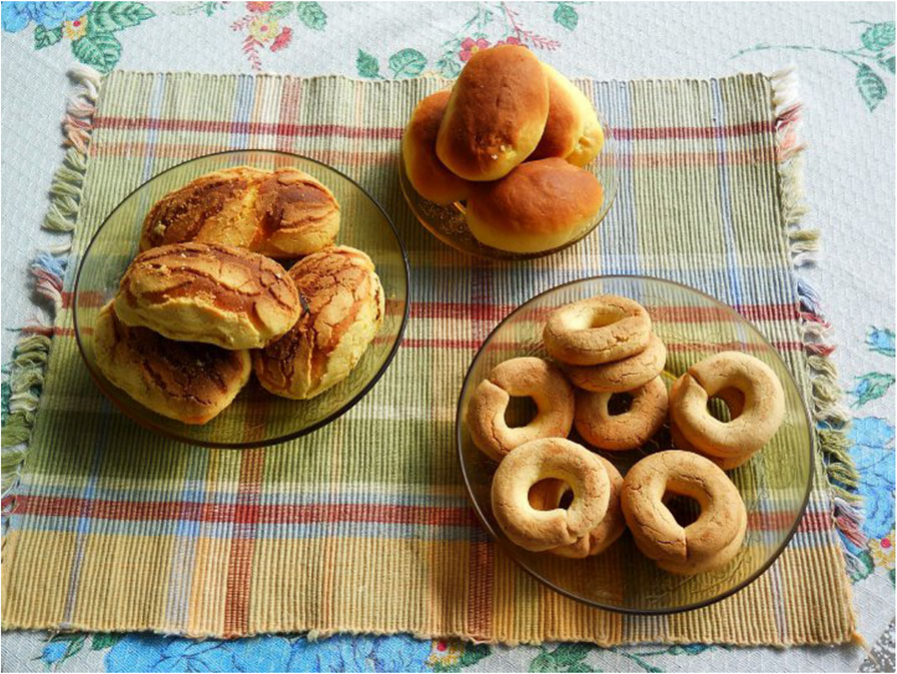
*Rosquetes* and breads made from sun-bleached *tiusinte* flour, Río Grande, Gualaco, Honduras. Part of a large batch prepared by local women for sale at the 2010 *tiusinte* fair (Feria del Teocinte). Such products include either maize or wheat flour, and are baked in outdoor clay-brick ovens. They are known only from Gualaco

Several foods are produced from cycad wet meal and dry flour (Figs. 10 and 11). The three basic foods are *tortillas* (griddlecakes), *tamales* (leaf-wrapped, sometimes filled breads), and *atoles* (slurries), made from wet meal *nixtamal* through the grinding, leaching and boiling process described above, known as *nixtamalización* in Spanish. This term is most commonly associated with maize, though in the latter case, it does not involve the complicated detoxification measures and tests needed for cycad preparation. Foods may also consist of cycad meal mixed with maize flour or, occasionally, other ingredients. In Honduras, Old World-derived ingredients such as wheat and chicken eggs are often added to create various oven-baked bread products, some leavened with baker’s yeast.

Carbohydrate-rich cycad foods are very filling, and in some cultures, particularly Querétaro mestizos, Xi’iuy, Honduran mestizos, and Chinantec, they are still highly valued and sought after by thousands of people. Cycad *tamales* may even attain the status of offerings in altars for religious ceremonies, such as among the Xi’iuy for Day of the Dead (Nov. 2), when food is put out for the ancestors while at the same time cycad leaves are used for decoration. During Holy Week (Semana Santa) in some NE Honduras towns, cycad foods are served and eaten together with maize foods while cycad leaves adorn Stations of the Cross installations. If cycad seeds are stored dry—the case with *tiusinte* and *chamal*—they can be further processed and consumed throughout the year.

**Fig. 12:**
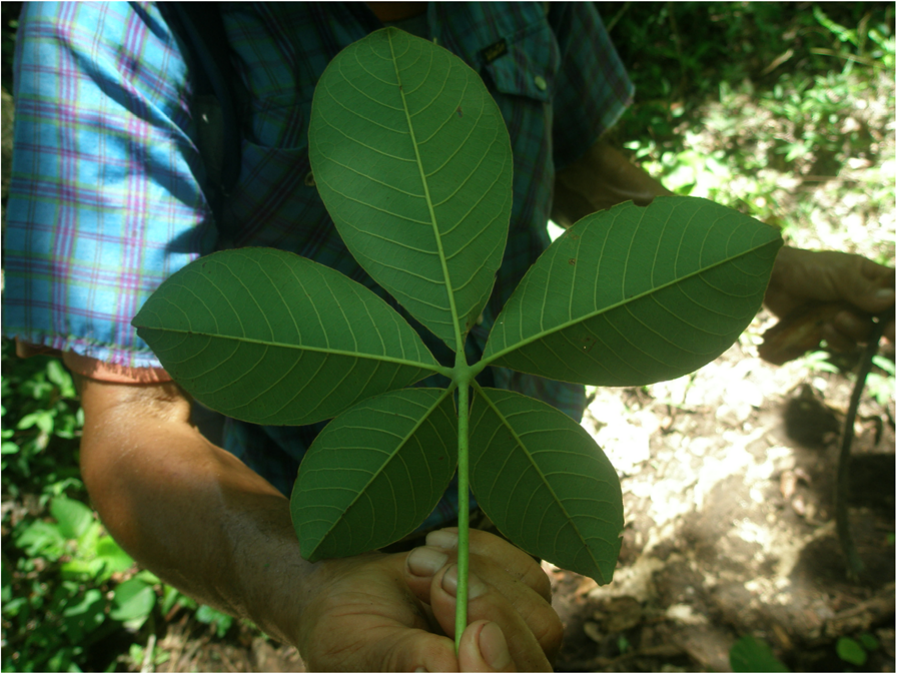
*Macoque* (*Pseubombax ellipticum* (Kunth.) Dugand) leaf. Leaflets of this species are the preferred wrap for *chamal* tamales in the Sierra Gorda, Querétaro. Photo was taken in a Sierra Gorda emigré community in the Huasteca lowland region, San Luis Potosí, 2009, where *chamal* (*Dioon edule*) also grows and is used for food by mestizos, while eschewed by neighboring Teenek people

Among some *tiusinte* and *chamal* consumers—Honduran mestizos and Xi’iuy in San Luis Potosí in particular—cycad foods are regarded as superior even to maize foods. In both cultures, *tamales* are the most important cycad foods, and though their final forms are quite distinct—Honduran *tamales* are long and cylindrical, while Xi’iuy *tamales* are the shape and size of goose eggs—detoxification processes, tree species used for ash and leaves used for leaf wraps are nearly identical, and the two most admired qualities of well-prepared and fully detoxified tamales are the same: rubberiness (*hulosidad*) and a ruddy color (Fig. 12).

### Importance of cycads in traditional land management

**Fig. 13:**
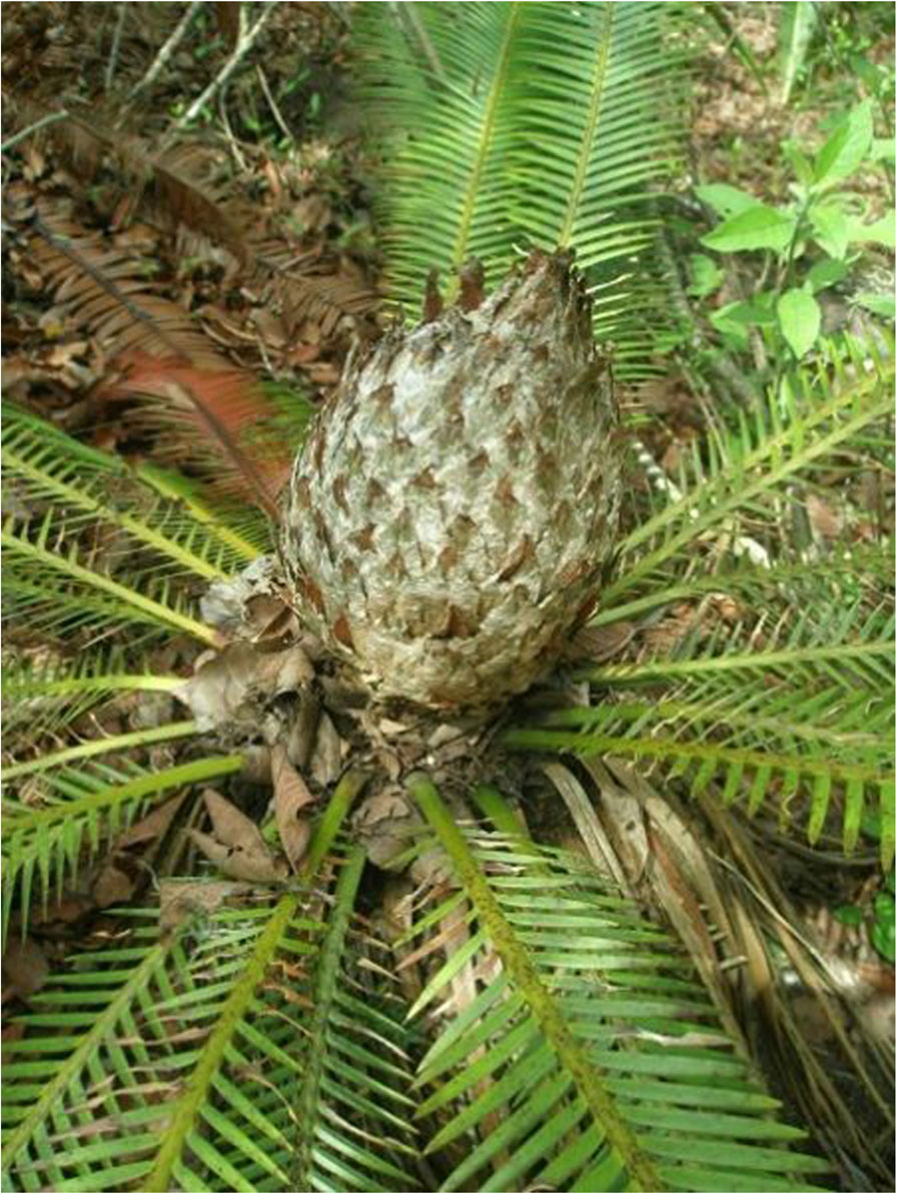
Trunkless female *chamal* (*Dioon edule*) with mature cone just prior to harvesting, Pamería region, San Luis Potosí, 2009

**Fig. 14:**
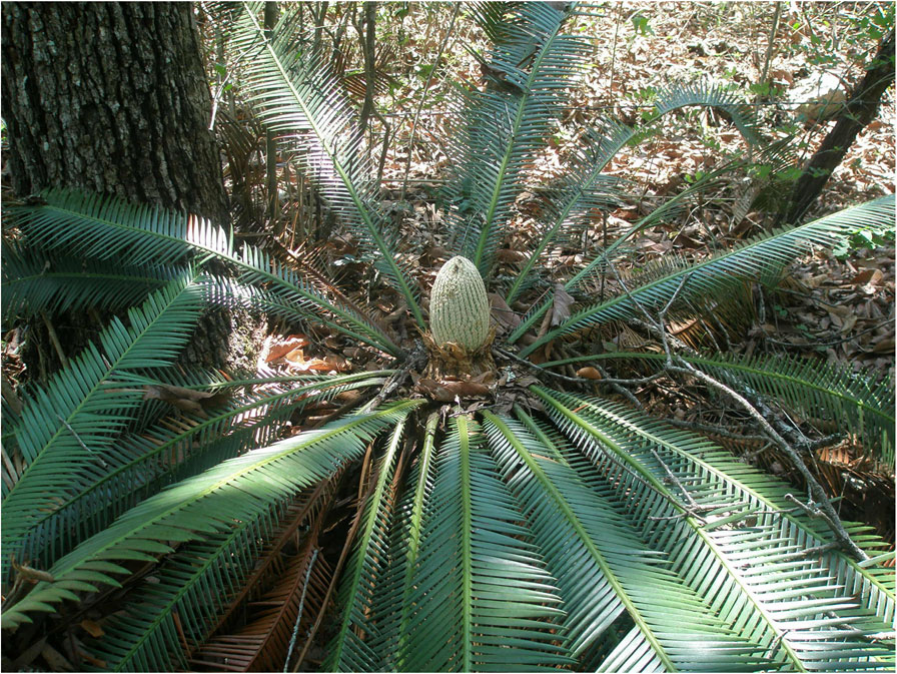
Male *chamal* (*Dioon edule*) with immature pollen cone, Pamería region, San Luis Potosí, 2009

**Fig. 15:**
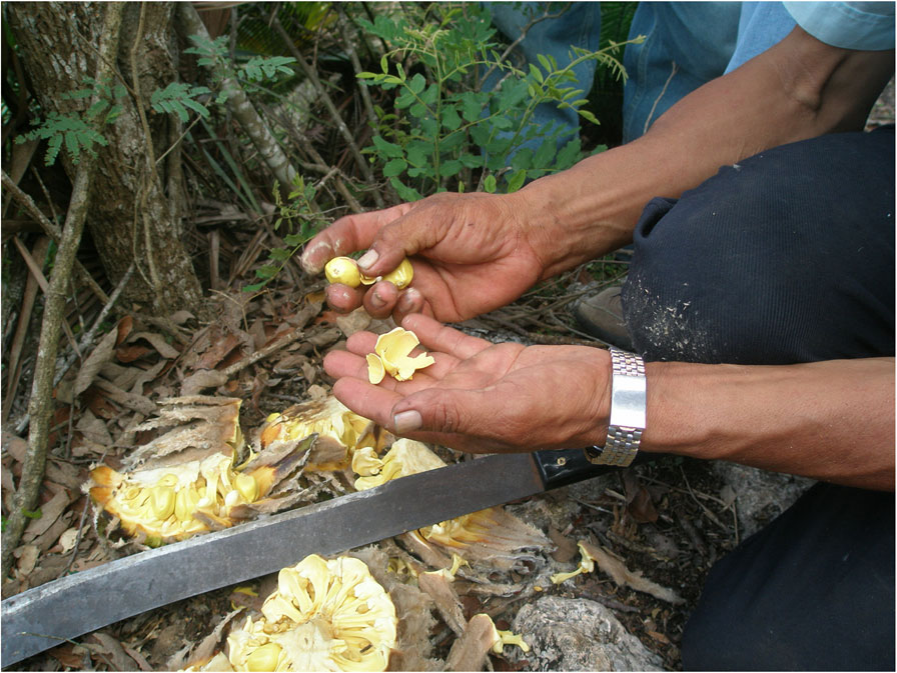
A *chamalero* demonstrates the process of splitting a *chamal* cone, extracting the seeds, and peeling away the sarcotesta, which is edible raw. Pamería region, San Luis Potosí, 2009

**Fig. 16:**
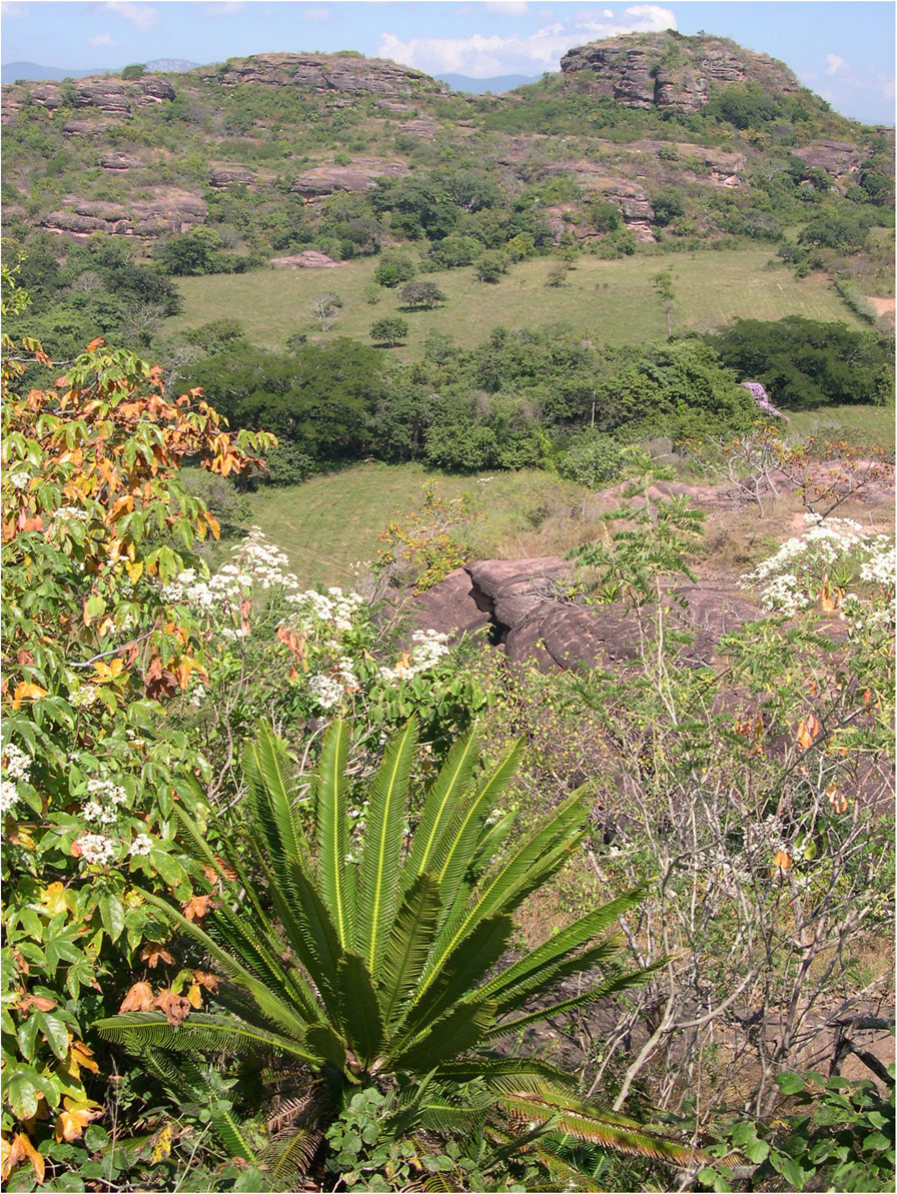
*Espadaña* (*Dioon merolae*) growing in an arid landscape near Tuxtla Gutiérrez, Chiapas, 2003

**Fig. 17:**
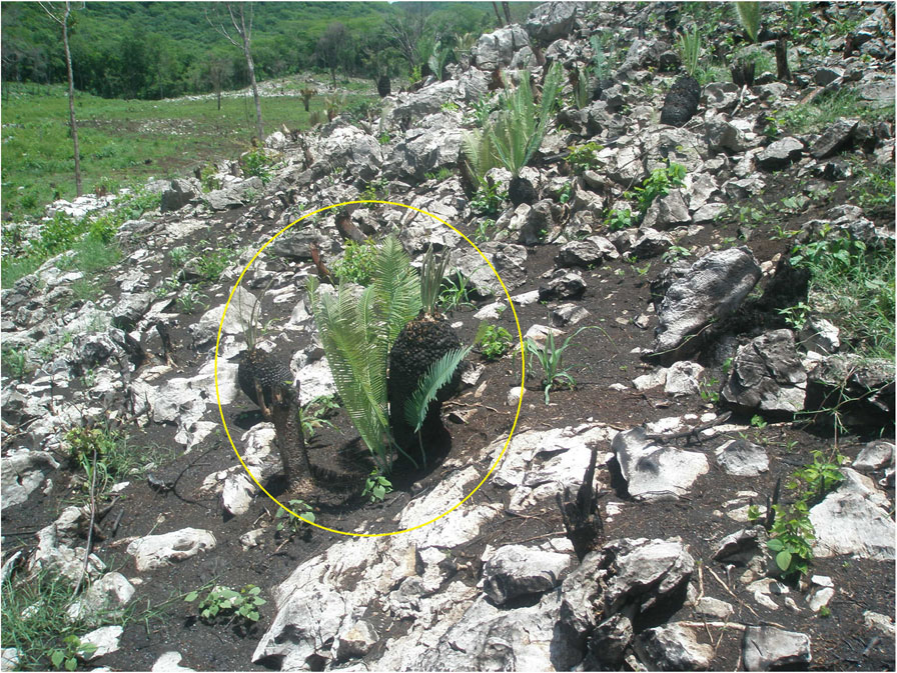
*Chamal* (*Dioon edule*) grows in a rocky fallow maize field in the Huasteca lowlands, San Luis Potosí, 2009. While in this case, *chamal* plants are not being purposely protected, they are able to survive agricultural burns and sprout new leaves

**Fig. 18:**
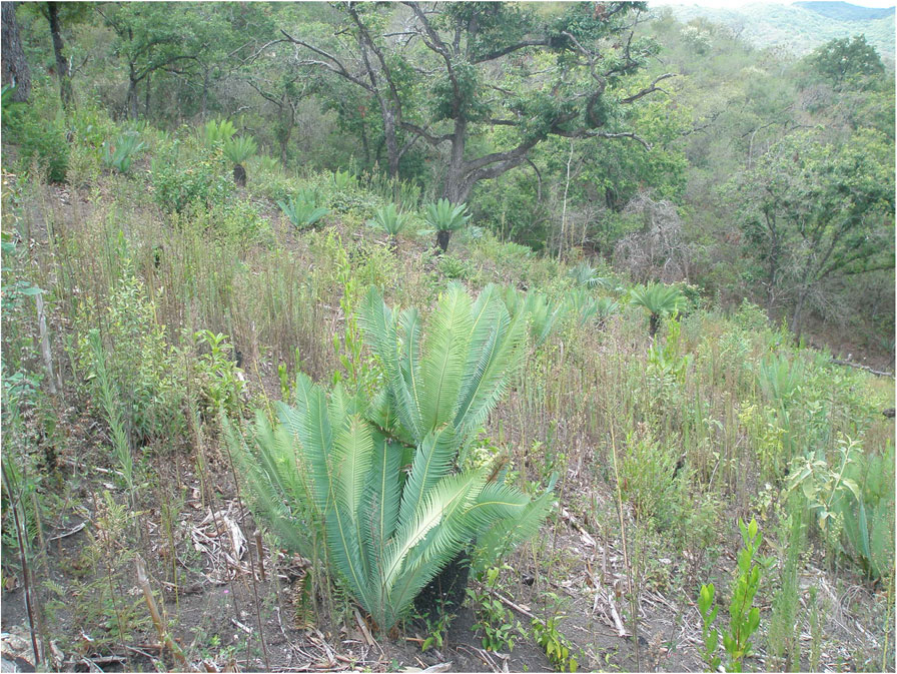
*Chamal* and maize “intercropping” in a fallow field, Pamería region, San Luis Potosí, 2009. *Chamal* leaves may be used for a range of religious and secular activities, and depending on the length of the fallow period, *chamal* may bear cones for harvest as food

This section draws from data listed in the “Agroecological uses” column (Additional file 1: Table S1), and is informative for community cycad conservation (Figs. 13, 14, 15, 16, 17, and 18). As a general rule, landowners eradicate MNCA cycads (even in areas where others harvest them traditionally, sometimes creating conflicts) if they interfere with land management strategies or out of disregard for them where they are perceived as having little or no value. However, most commonly in NEMX and Honduras, we confirmed that local people incorporate at least 26 species of cycads in traditional, intentional land management practices (exclusive of the sacred maize-cycad relationship, described in a later section). Agroecological management strategies may be for purely utilitarian reasons, typically evinced as the case in Honduras, or incorporate sacred dimensions, as among Teenek and Nahua in NEMX and among Chiapanec people in Chiapas. Reasons to manage cycads are focused on their food value and are specifically (1) to improve cone yields, (2) to mitigate damage to leaves from harvesters, (3) to balance cone production and maize production, where the two are intercropped, and (4) to balance with the needs of cattle.

Local beliefs that people planted present-day cycad populations in the distant past exist in Honduras (we did not encounter the belief in Mexico). Terms such as *finca* (farm) and *plantación* carry this connotation, distinguishing cycads from other harvested wild species not thought to have been planted by humans. Such cycad “farms” and “plantations” are usually treated as common-property resources even where found on private lands [52].

The principal intentional engagement of people with cycads in the landscape takes place in the context of the cone harvest. In most places today, cycad populations are accessible by road or by a day-trip on foot. Previously, when fewer roads existed, cycads were located up to several days on foot away from communities, and cycad gatherers, sometimes with their families, and occasionally entire villages, relocated to seasonal cycad-harvesting camps. Taller species, such as *tiusinte* in Honduras, have to be scaled to reach cones, a risky process that entails finding a foothold on the helpful notches carved into the trunks at some point in the past. While typically an activity performed by men, women also recounted harvesting cones in the past. Leaf crowns are typically partially removed to get at the cones, which are twisted off and then broken apart in situ, and the sarcotesta-covered seeds extracted, placed in sacks, often loaded onto beasts of burden, and taken back to permanent residences for processing as described above.

In both NEMX and Honduras mestizo communities, a common belief is that in years that maize is abundant, cycad cones are not, and vice versa, showing the complementarity of the two resources even where the ritual compatibilities of cycads as maize ancestors have been forgotten. Desperately hungry families may destroy immature cones in their search for viable cycad food, resulting in a diminished harvest for others. As an attempt to control this behavior and make harvesting more equitable, municipal authorities in certain Honduran communities once enforced a seasonal ban on harvesting, and there have been recent attempts to restore the practice [7].

Local people with substantial agroecological knowledge recognize that cycads are very hardy, and that low-level fires stimulate leaf flushes. In certain locations, people also recognize that careless removal of leaves, particularly by machete, can damage the plants. Because cycads are so hardy and fire-tolerant, they are often the only plants to survive in burned-over maize fields after all other vegetation has been eliminated. In Honduras, maize and *tiusinte* cycads are intentionally intercropped and in a few cases leaves that shade out maize plants are selectively and carefully removed to avoid damage.

Struggle often occurs between cattle ranchers and cycad harvesters. In Honduras, few interviews with cattle ranchers revealed any concern that *tiusinte* was poisonous to cattle to the extent that *Dioon* is in NEMX. Honduran ranchers simply destroy *tiusinte* as they do any woody plant that is not necessary in a pasture—by repeated burning, cutting, and poisoning. Nevertheless, recent advances have been made in the Gualaco municipality to reverse this approach, for example through the collaboration of cycad biologists, agronomists, and biologists, and through public education and awareness at events such as cycad fairs (see *tiusinte*, Additional file 1: Table S1). In NEMX, *enchamalamiento* (cycad intoxication) caused widespread loss of stock, particularly from the effects of hind-leg ataxia. Governmental authorities in the past thus considered cycads to be noxious weeds, the “hierbas locas” (crazy weeds) reported by Reko [74], and stimulated campaigns to eliminate *chamal* from vast lowland areas. But in San Luis Potosí villages such as Gamotes and Agua Puerca, the two resources are rendered compatible in traditional land management regimes. In these communities, where *chamal* is still highly valued as a food resource among traditional Xi’iuy and mestizo groups, cattle are penned up and thus not allowed to come into contact with *chamal* when new leaves flush, because it is said that the cattle tend to seek out the tender leaves and are poisoned as a result [72].

Other traditional practices across the region include occasional use of sarcotestas as well as detoxified seeds for domestic animal feed, preparation of a rodenticide from the raw seeds to control crop pests (primarily rats and mice), and use of leaves for thatch in the temporary field shelters erected by farmers.

A final important practice in this category is cycad cultivation in home gardens. We documented the practice where it appeared to be traditional but did not include plants bought in commercial nurseries. Traditional reasons to grow cycads in home gardens, beyond their purely ornamental value, are for access to leaves for religious and secular ceremonies, and occasionally, for access to starch for food (though this presumes enough domestic male and female plants are present in a community to sustain the beetle pollinator population necessary for ensuring fertilization and maturation of female cones). In communities distant from cycad populations, where cycads are nonetheless highly valued, as is the case in Juticalpa, Honduras [14], dozens of cycads exist in home gardens and often pre-date the houses themselves.

### Limited and local traditional economic importance

We found seven MNCA cycad species that played some role in the cash economy as a minor food commodity (“Economic uses” column, Additional file 1: Table S1). In local, traditional economic contexts, cycad seeds have been incorporated into local and occasionally regional market contexts. In areas with heaviest food usage, such as Gualaco, Honduras, cycad gatherers constitute a seasonal, itinerant, informal labor pool. Some men supplement their income by gathering and selling cycad seeds to households, and interviewees commonly mentioned that such *tiusinteros* utilized the income to purchase alcohol for personal consumption. Seeds also can be found in certain town markets, such as Olanchito in Honduras and in San Luis Potosí and Querétaro. Food products are occasionally offered for sale as well—for example, local Xi’iuy people regularly sell *tamales de chamal* to mestizos in the central plaza of San Antonio Tancoyol, Querétaro. We have not yet found recent evidence of cycad seeds or foods being sold in large regional markets distant from areas of traditional consumption. Leaves, though heavily utilized in religious ceremonies throughout the region, are not commonly offered for sale, and the leaf trade for local use, with the possible exception of the Tehuantepec region of southern Mexico, does not appear to have a significant effect on wild plants.

### Non-religious decorative uses

**Fig. 19:**
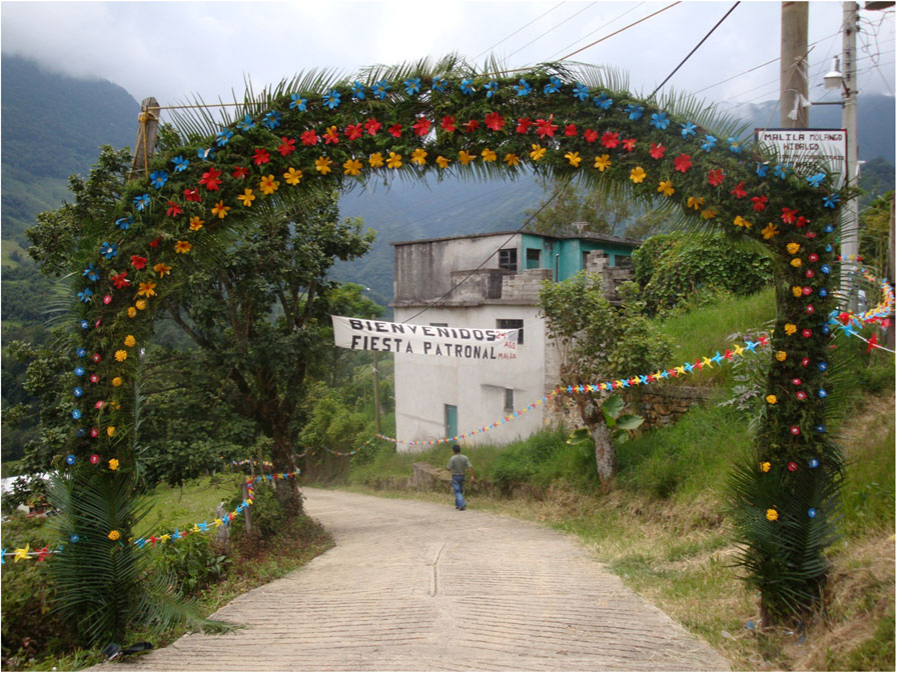
Archway welcoming visitors to the town fair of Malila, Hidalgo incorporates *teocintle* (*Ceratozamia fuscoviridis*) leaves. Photo by AR

**Fig. 20:**
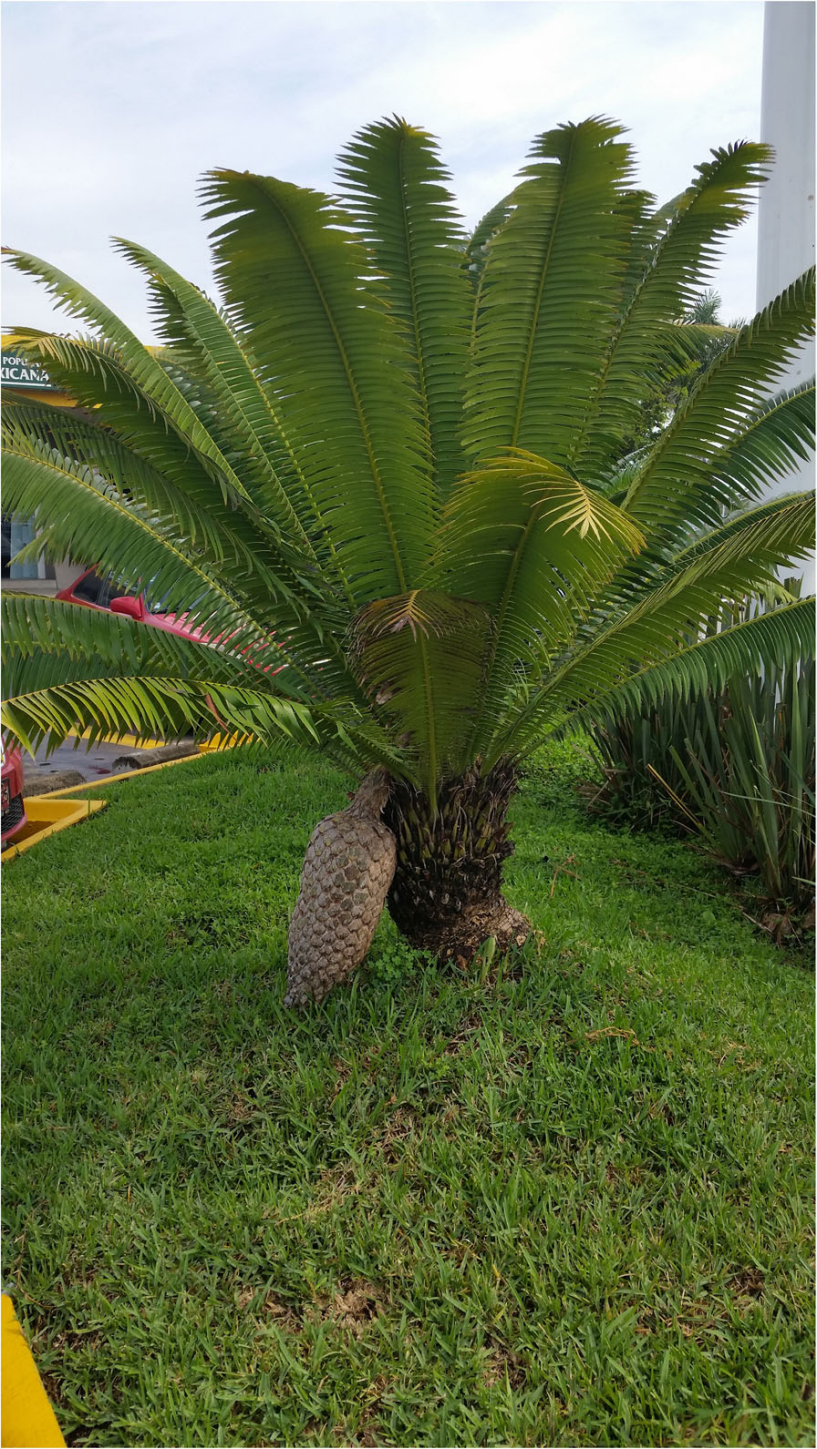
Female *Dioon spinulosum* with large seed cone in a commercial park, Tepic, Nayarit, 2016. The species is not native to the region and was likely transplanted

**Fig. 21:**
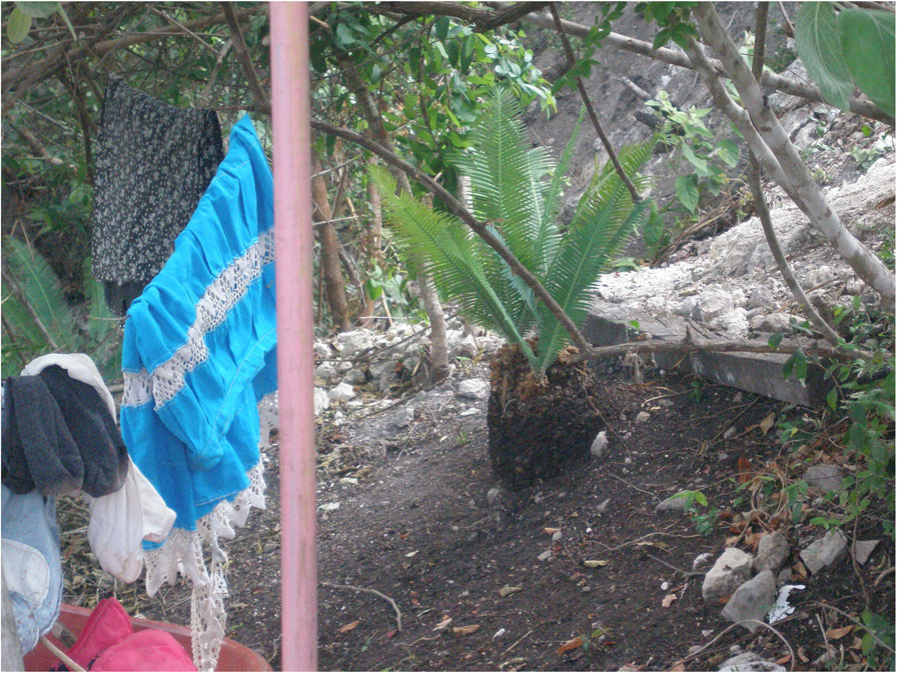
*Chamal* (*Dioon edule*) in a house patio, Pamería region, San Luis Potosí, 2009

**Fig. 22:**
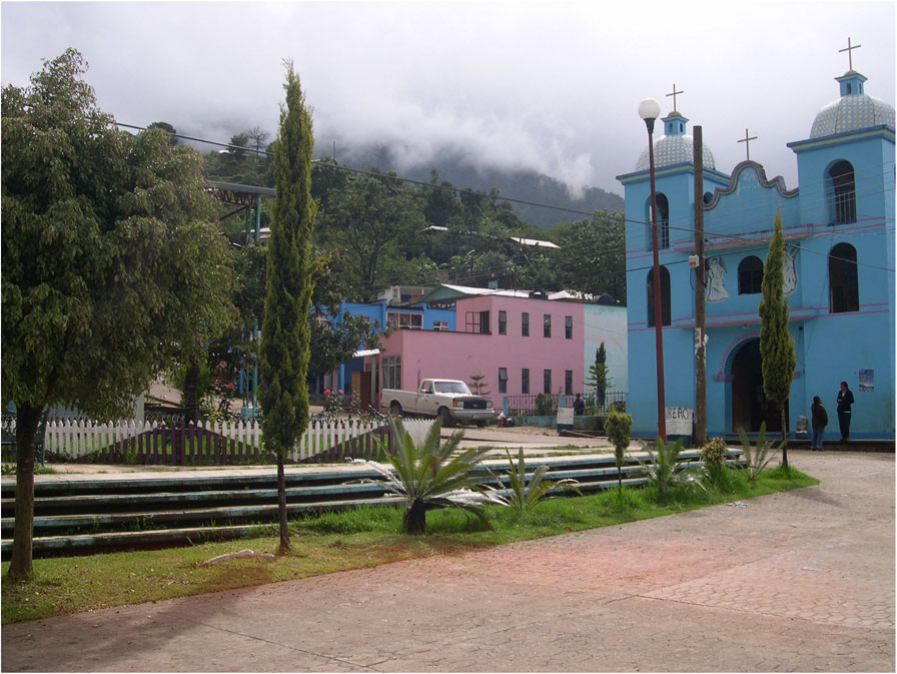
*Yazn-gua* (*Dioon holmgrenii*) planted in town plaza of Santiago Textitlán, Sierra Sur, Oaxaca, 2008. These had been transplanted from a community nursery as a show of pride in community-based cycad conservation efforts underway in the area. See also Fig. 29

In traditional as well as commercial contexts, cycads are highly valued as ornamental plants in public and private landscapes, and native species are often mixed with Old World cycad species (Figs. 19, 20, 21, and 22) (see “Other uses” column, Additional file 1: Table S1). Many MNCA communities where cycads are highly valued contain permanently transplanted cycads in public plazas and other highly visible locations. Otherwise, decorative uses not directly associated with religious ceremonies tend to involve transitional events. In NEMX, cycad leaves are commonly utilized to construct processional arches for wedding receptions and graduations, suggesting a connection to symbolic value as plants that help effect or ease transitions between life stages. Though we documented eight species in this category, we suspect many others are used this way in areas we did not visit.

As mentioned above, the trade in live cycad seeds and plants that occurs from small to massive scale across the region was not covered in the present study. Nevertheless, what we encountered during field research bears brief mention. We often found that cycads—whole plants or parts—are extracted to be sold in local nurseries, and typically purchased by people from urban areas for purely decorative purposes. Whole plants and seeds are also trafficked illegally across international borders. In one of the most egregious cases we encountered, *Dioon tomasellii* populations, bereft of local ethnobotanical value, ownership, or even knowledge of traditional names or uses, are extracted wholesale by middlemen from the hills near the resort city of Puerto Vallarta, Jalisco. Local people in a rural village near a previously undocumented cycad population asked us point blank if we were interested in buying cycads, because they earn income by helping extract cycads for this illicit business. The cycads are transplanted to resort hotels in Puerto Vallarta. We found no evidence of government knowledge of or interference in the activity. To combat this type of lucrative trade, various efforts have been made across Mexico to establish profitable community nurseries [5, 75].

### Minor uses for ethnomedicine, narcotics, magic, and other purposes

We confirmed present or past ethnomedical uses for 12 species (“Other uses” column, Additional file 1: Table S1), but we have not yet studied cycad ethnomedicine directly, relying instead on published reports and interviewees’ accounts. The physical properties of the sticky mucilage and downy tomentum on the female cone favor uses such as stanching and covering wounds and other skin lesions. Use of mucilage to treat joint disorders has been mentioned for *D*. *sonorense*. Skin conditions are often the focus of cycad ethnomedicine; in this, a comparison can be made to the recent finding that cycasin in *Zamia ulei* used traditionally in the Peruvian Amazon in medicines derived from stems is moderately effective against leishmaniasis [76].

Narcotic and putative entheogenic uses for six species came to light in this study (“Other uses” column, Additional file 1: Table S1), ranging from intoxication resulting from ingesting *sotol* cycad starch-derived alcohol to hallucinatory effects produced by *peyote* cycads in WMX (*Z*. *paucijuga* and *D*. *sonorense*) and in certain *Zamia* and *Dioon* species in NEMX. The use of the term *peyote* is revealing, as it has previously been mentioned almost exclusively in the literature as referring to the cactus *Lophophora williamsii*, a powerful entheogen utilized by the Wixárika (Huichol) and other Indigenous groups [77]. Though we have not observed usage first-hand, interviewees insisted that consumption of *peyote* cycad parts also produces powerful narcotic effects. According to informants in Bacanora, Sonora, Yaqui warriors once ingested cycad *peyote* to produce a type of battle rage before going to war. In NEMX, cycad parts are used as recreational drugs, and also by shamans (primarily Nahua and Teenek) who are said to ingest them for entheogenic purposes. Such practices are extremely secretive, explaining why these data have not previously come to light. In addition to ingestion for these reasons, Teenek and Nahua shamans were reported to us to utilize *Dioon* leaves in certain ritual ways to magically produce ill effects in targeted victims; the exact process for producing as well as reversing the harm was described to us.

**Fig. 23:**
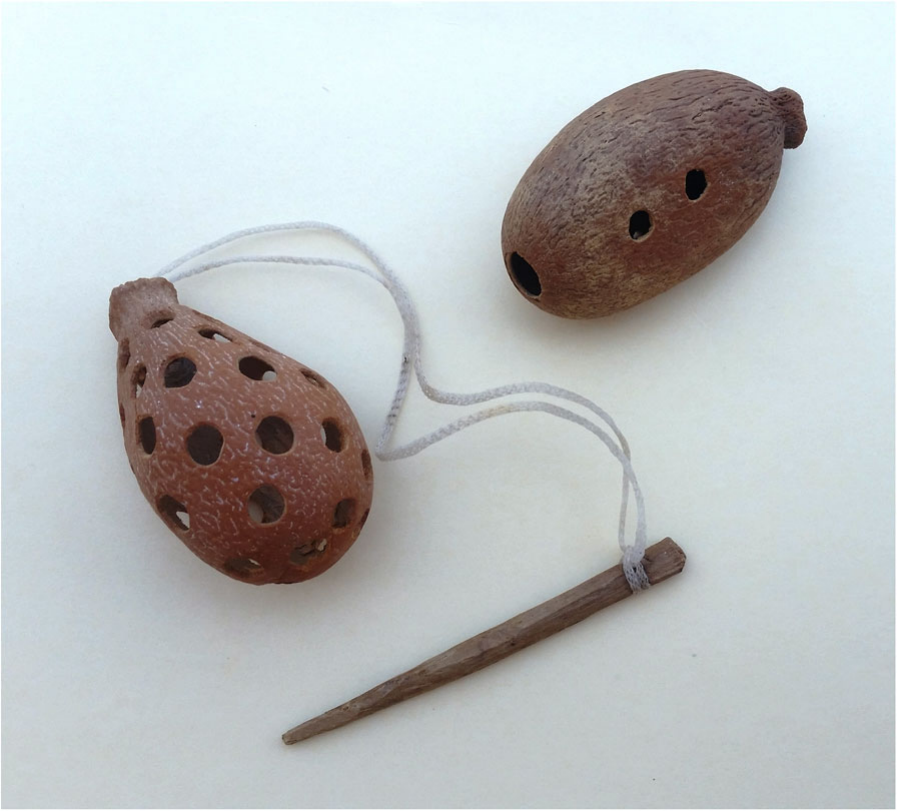
*Tiusinte* cycad toys made from hollowed-out, fresh *Dioon mejiae* sclerotestas. L.: *Enchute* (pin-and-target); R.: whistle

Hollowed sclerotestas of eight species are used widely as toys that include whistles and pin-and-targets, reflecting their sonorous properties, overall durability, and spherical to ovoid shapes (Fig. 23). Such a sclerotesta (“nut on a stick”) was discovered in La Perra, utilized several centuries ago for an unknown reason [12]. Dye and coloring agent uses have been reported for two species, as well as envelope glue (from cone mucilage: one sp.), weaving (leaves: one or two spp.), laundry starch (one sp.), as combs (dry male cones: one or two spp.), and for construction (five spp.). Eleven *camotillo* species have purportedly been used for assassination.

### Cycads in religion

**Fig. 24:**
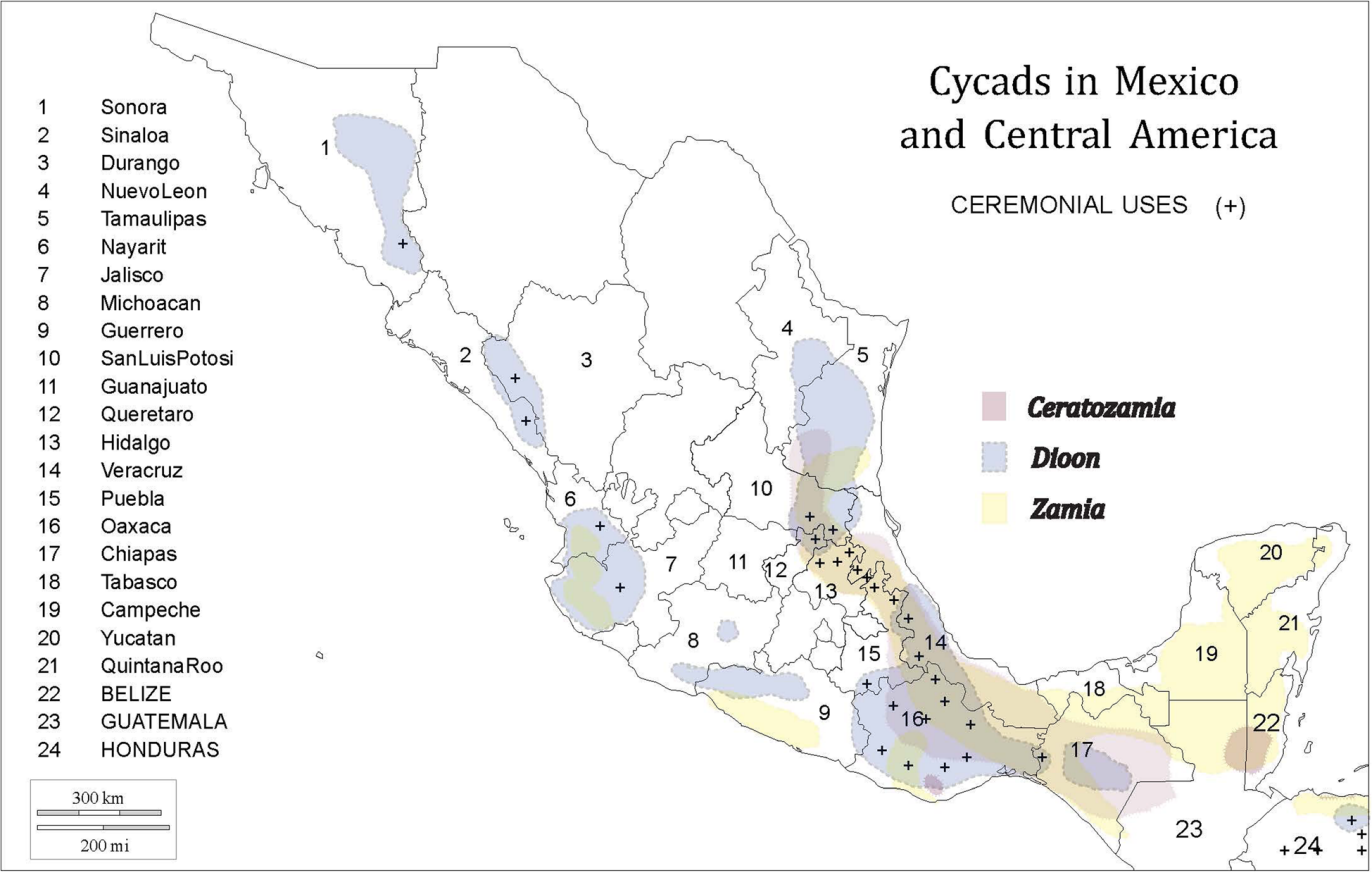
Ceremonial uses of cycads in Mexican and Central American religion

**Fig. 25:**
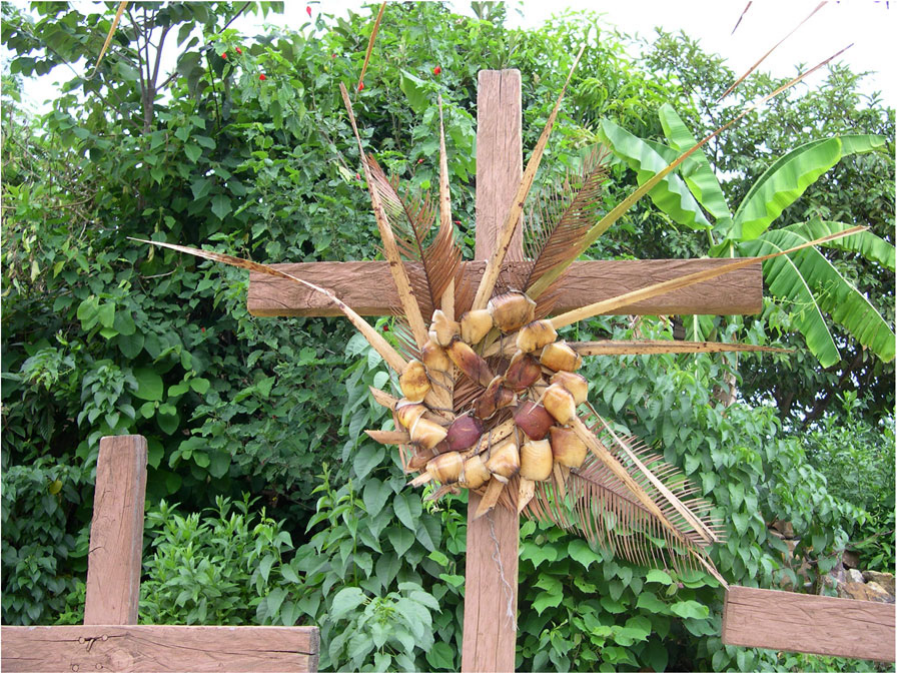
A station of the cross incorporating *palma de coyote* (*Dioon* sp.) leaves, San Jerónimo Taviche in the Valles Centrales of Oaxaca, 2008. Decoration had been left up for over a month after Holy Week (Semana Santa). Leaves derived from a population protected on the Zapotec community’s *ejido* (communal land)

**Fig. 26:**
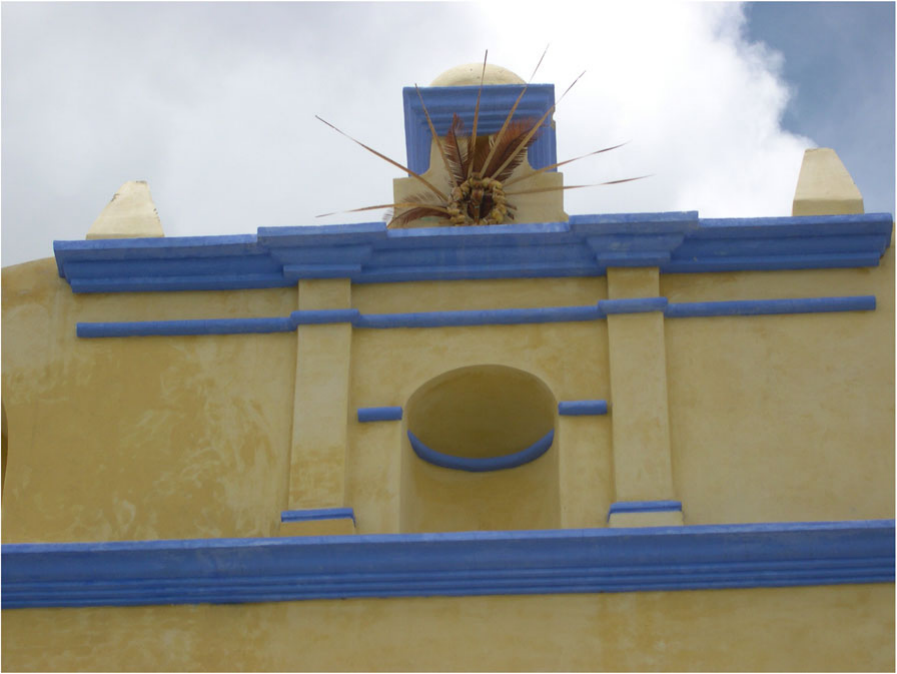
*Palma de coyote* (*Dioon* sp.) decoration on town church, San Jerónimo Taviche, Oaxaca, 2008

**Fig. 27:**
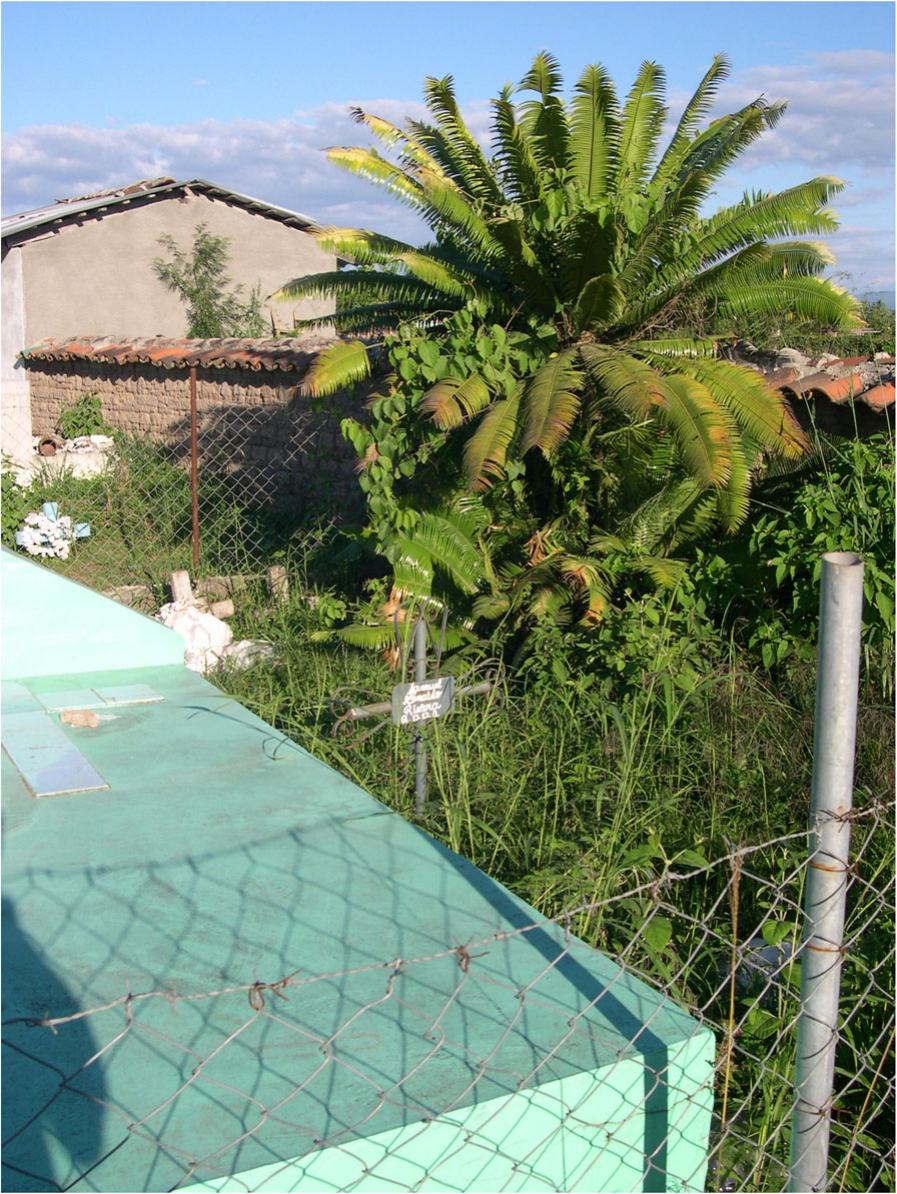
*Tiusinte* (*Dioon mejiae*) in a graveyard in Punuare, Olancho, Honduras as a convenient source of leaves for Day of the Dead wreaths

**Fig. 28:**
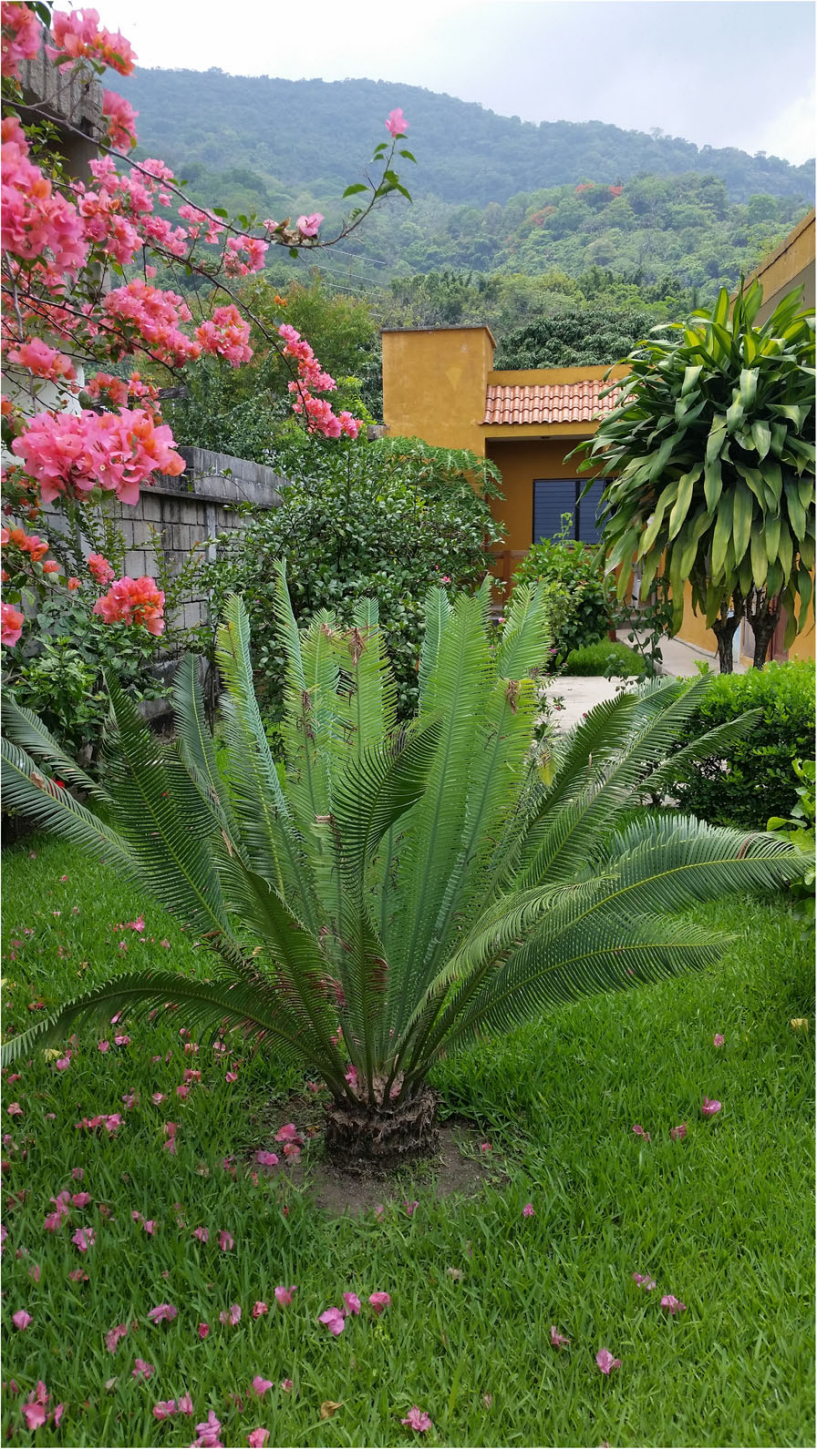
*Tzamaalib* (*chamal*, *Dioon edule*) in a yard as a source of leaves for religious ceremonies, Tancuime, Aquismón, San Luis Potosí, 2016

**Fig. 29:**
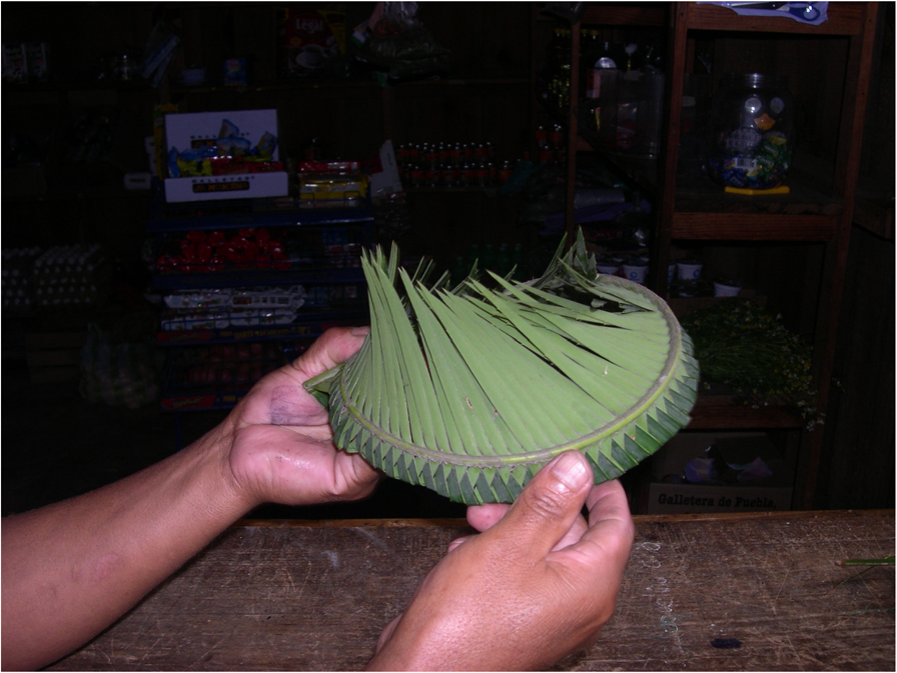
A crown of thorns fashioned from a *yazn-gua* (*Dioon holmgrenii*) leaf, Santiago Textitlán, Sierra Sur, Oaxaca, 2008. Such crowns are worn by boys in a Holy Week (Semana Santa) processional

**Fig. 30:**
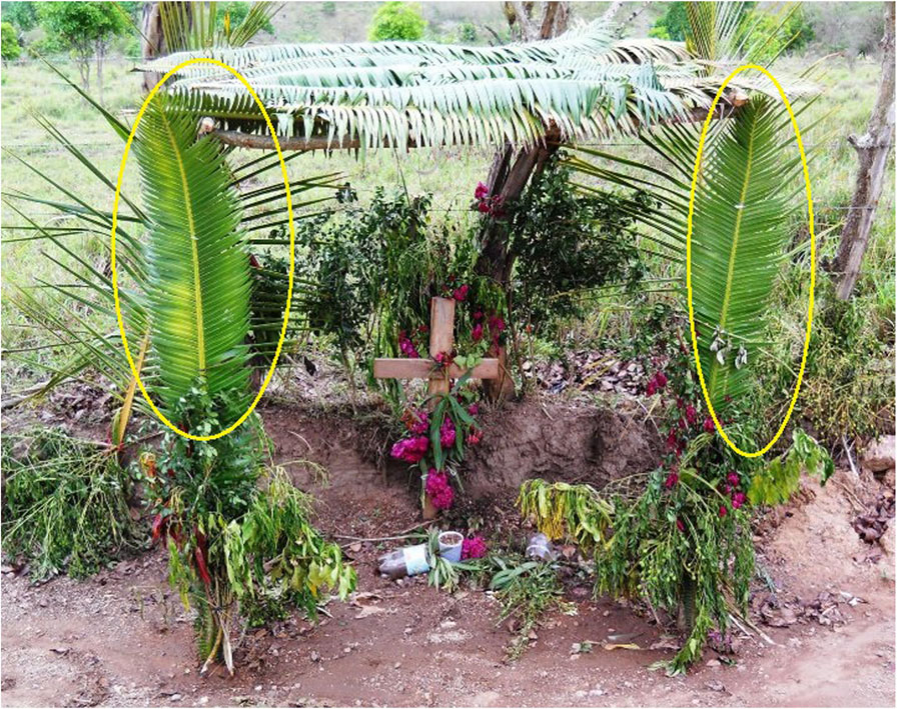
A station of the Cross utilizing *tiusinte* (*Dioon mejiae*) from 2010 Holy Week (Semana Santa) decorations in Río Grande, Gualaco, Honduras, taken during the *tiusinte* fair (Feria del Teocinte) a week later

An understanding of religious uses and beliefs (see “Religious uses” column, as well as most references to maize, Additional file 1: Table S1) is critical for conservation efforts, as they constitute the principal enduring reason that people know about and value local wild cycad species and are concerned for their welfare. We documented 16 cycad species used for religious purposes. The patina of Roman Catholicism is quite light in many Indigenous syncretic contexts, while Indigenous elements are very strong in many purely mestizo contexts, for example in November Day of the Dead celebrations (Figs. 24, 25, 26, 27, 28, 29, and 30). Old World and New World religious elements and a diverse suite of plant species intertwine with cycads in complex ways, particularly in the contexts of palms, as discussed below. The physical characteristics of cycad leaves—very durable and malleable, and not quick to fade once placed in an altar, wreath, or arch decoration—were often cited as key reasons for their usage. The spines of cycad leaves, in Santiago Textitlán, Oaxaca, for example, enhance their effectiveness as painful “crowns of thorns” worn by worshippers during Holy Week processions.

Symbolic significances of cycads in general have been discussed extensively by Bonta [22, 28] and point to imbued meanings deeper than simply as durable stand-ins for palms. Cycads are central to celebrations related to birth, death, transcendence of death, and conceptions of the afterlife—both of people as well as crops such as maize and the overall renewal of the fecundity of the land. This is due to human awe of their extreme longevity, hardiness, and importance as staple or famine foods in contrast to their extremely dangerous properties. Not least, humans have admired the phallic properties of the male cone, enhancing their status as powerful and important plants.

Names of cycads in a few places in WMX and NEMX—e.g*.*, “Devil’s palm”—seem to be directly at odds with many common terms relating them to sanctity (e.g., “Virgin Mary’s palm” or *palma bendita*, “blessed palm”). This juxtaposition of “good vs. evil” cycad conceptualizations reflects their originally elevated status in Indigenous contexts (as bounteous, sacred plants) that came to be branded as “evil” by Spanish colonizers. The latter believed that wild and particularly toxic foods such as cycads (among many others) were “bad” and uncivilized and thus should not be used or eaten. This stigma was applied, for example, by Catholic missionaries who made many attempts to settle *chamal*-dependent Xi’iuy gatherers of the Pamería and Sierra Gorda [78, 79] and force dependence on “civilized” agriculture.

But despite religious biases, Indigenous respect for cycads was not exterminated. The special case of the Chiapanec cycad pilgrimages [8, 80] bears comment here.^5^ This late-April activity, in Chiapanec culture, involves visits by *espadañeros* or *hojeros*, members of special guilds, to sacred sites such as the Cerro Nambiyugua mountain that contain certain sacred and protected populations of *Dioon merolae*, known universally as *espadaña*. Pilgrims harvest leaves without harming the plant, affix bundles of up to 80 kg on their backs, and walk back many kilometers and several days to their home communities, where worshippers greet them in *topada de la flor* celebrations. Many videos of these events in Suchiapa and Terán can be found on *Youtube*, e.g., [81]. Leaves are distributed to local altars, and among the Chiapanec of Suchiapa, the event culminates on the 3rd of May with Day of the Holy Cross (Día de la Santa Cruz) activities that include a herald song about *nimalari* (the Chiapanec word for *espadaña*, meaning “feather”) sung in the extinct Chiapanec language. The pride in this important element of cultural heritage is widely apparent in the increasing amount of video evidence and commentary available online. Further study of such cycad pilgrimages is necessary to understand the deeper reasons for cycad use during a Catholic festival of relatively recent invention; we suggest that pilgrimages may have originated with Mesoamerican land fertility rituals given that early May is associated with maize field preparation prior to the onset of the first rains, particularly in southernmost Mexico and Central America.

### Associations with palms

Cycads are culturally associated—in agroecological and ritual contexts—with many other plant groups, ranging from *Pseudobombax* to *Beaucarnea*, *Dasylirion*, various ferns, and so forth. Most of these relationships, evident in local terms for cycads, have barely been explored (see Additional file 1: Table S1). The most significant association, with maize and its relatives, is discussed in the next section. We frequently encountered, and thus briefly highlight here, cycads’ confusing relationships with palms, with which they are often grouped—both by indigenous people possessing extensive knowledge, and by casual observers confusing them due to their physical similarities.

We found 32 distinct terms for MNCA cycads that related them to palms, by far the prevalent nomenclatural association with another plant, after maize. This profusion is most obviously related to the physical resemblances of palms and cycads and many such names may be of quite recent origin or idiosyncratic to individual interviewees. At a deeper level, though, some names reflect the interchangeability of cycads and palms in Roman Catholic religious events, whether for simple expediency or as continuations of pre-Christian uses of cycads at temples and other sacred sites. In essence, palms were sacred in both Old and New World cultures prior to contact between the two regions, while cycads were unknown to Spanish colonizers. After colonization, knowledges and traditions intertwined, thus making it difficult to say whether a specific cycad-palm connection such as a term or a use in a religious altar predates colonization or is of more recent derivation.

The question of whether cycads are conceived as types of palms in MNCA Indigenous and Indigenous-derived cultures is highly contextual. For example, among the Otomí-Ñuhu, the Teenek, and certain Zapotec groups, cycads are in essence thought to be a type of palm (in some cases, cycads are even seen both as types of palm and as types of maize). Indigenous language nomenclature suggests that cycads-as-palms predates recent Roman Catholic influence. In Honduras, by contrast, we found that even while popular wisdom held that cycads were types of palms, knowledgeable users in areas with native cycad populations were typically insistent that cycads were not palms.

The distinctions, or lack thereof, are highly significant for conservation, given that confusion between the two groups, if not based in deep cultural traditions, can be catastrophic when their differential growth rates are considered. This is best illustrated by frequent cases we encountered in Honduras, where uninformed recent in-migrants assuming cycads to be a type of fast-growing palm simply chopped down thousand-year-old cycads to get at the cones, just as they would local palm species harvested for their nuts. We have observed entire cycad populations razed to the ground in this way, under the assumption that they will grow tall again in a few years. By contrast, knowledgeable and aghast local people who showed us these cases were aware that female cycads can take 50 or more years just to produce a cone and begin to grow a stem.

In the arid Xi’iuy cultural zone of San Luis Potosí known as the Pamería, *chamal* is closely associated with the palm *Brahea dulcis*, which also has a variety of uses. Both plants are of considerable importance in local culture. The striking complementarity of the two species extends to a form of barter—palm resources (leaves for thatch, for example) have long been exchanged for cycad seeds. Indicating deeper relationships, one traditional treatment (*remedio*) for human *enchamalamiento* is consumption of palm ash, or of soil in which palms grow [72].

### Deep connection between cycads and maize

**Fig. 31:**
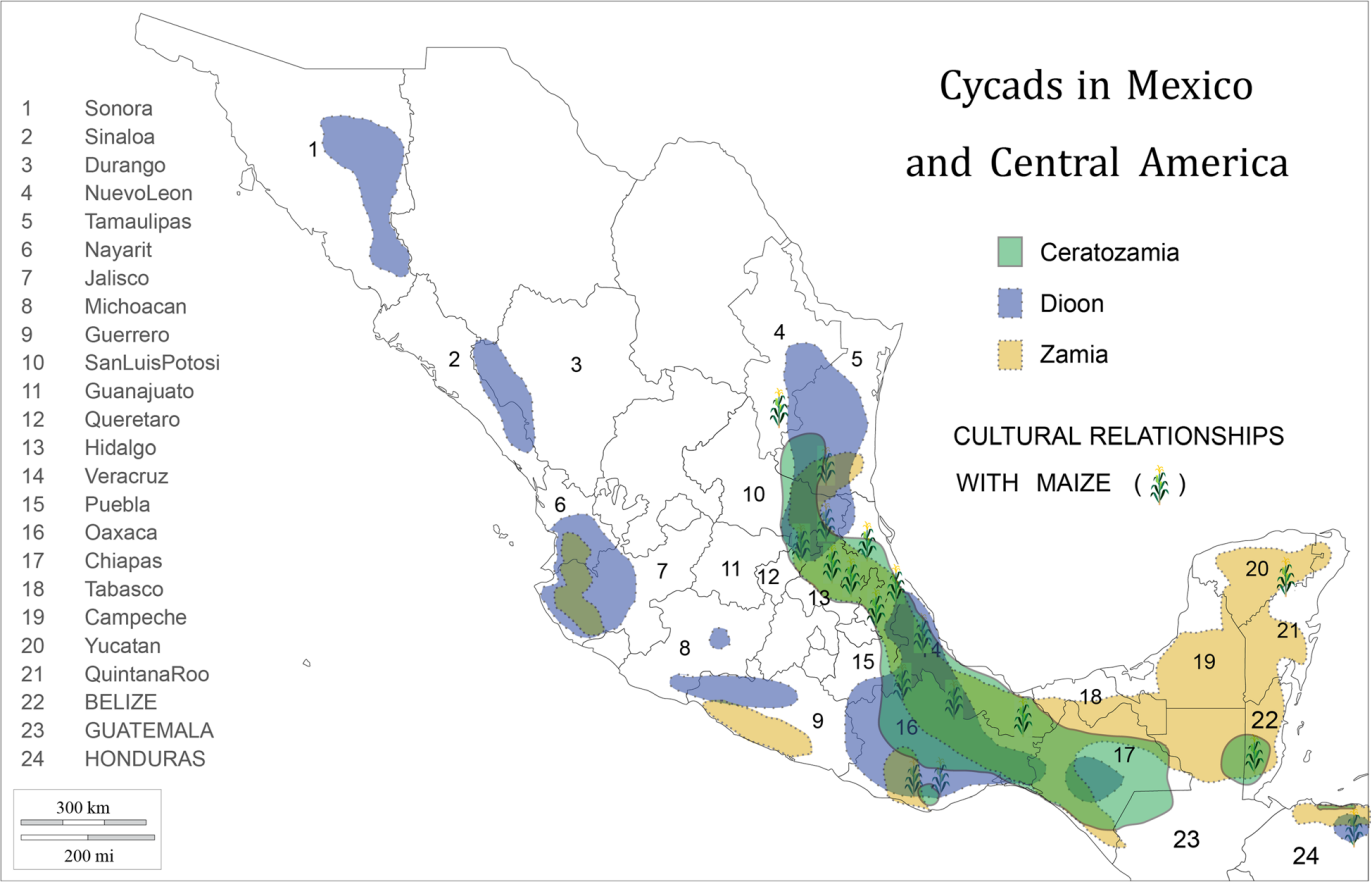
Cultural relationships between cycads and maize in Mexico and Central America

**Fig. 32:**
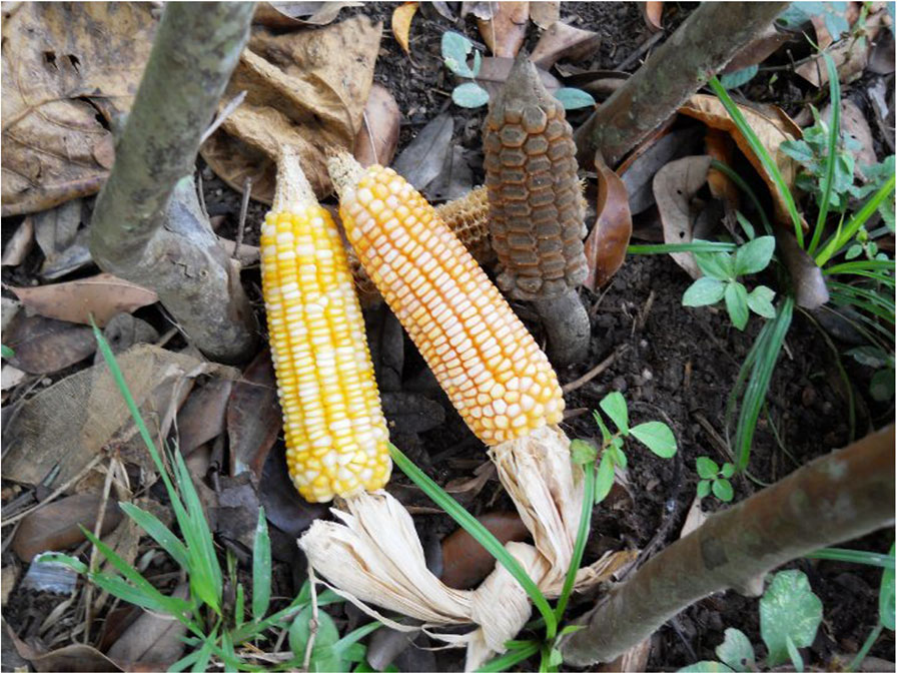
Comparison between the *mazorcas* (female cones) of maize and *Zamia* in Honduras

Our most intriguing research findings involve the associations made between 20 species of cycads and maize (Figs. 31 and 32) (*Zea mays mays*) (Additional file 1: Tables S1 and S2). Though physically similar in certain ways (male cycad cones, especially those of *Zamia*, and maize ears are superficially quite similar in appearance), maize and cycads are phylogenetically distant and cannot be interbred. Nevertheless, numerous terms and concepts situate cycads as progenitors and protectors of maize among the Xi’iuy, Teenek, and Nahua of NEMX and among the Chontal de Oaxaca. In terminology and ritual, but not at all to the extent or the depth seen among the above groups, the concepts are also present among *tiusinte* users in Honduras and among NEMX mestizos. We have detected further indications of cycad-maize connections from nomenclature in Totonac, Mazatec, Chinantec, Zoque, and Zapotec cultures in Mexico as well as in various cultures of northern Guatemala and Belize. Conversely, we also determined that no maize-cycad connections were present in the Zapotec communities we visited, nor anywhere in WMX, Chiapas, or the Yucatán, though our searching has not been exhaustive and it is fully possible that they were once present but have disappeared. Future publications will delve into greater depth on this tradition as found in its most complex forms in NEMX; a brief summary of our research among the Teenek and Nahua appears below.

In Teenek communities of San Luis Potosí, widely varying knowledge exists of the roles and importance of the *konlif* cycad (*C. latifolia*), though such knowledge is disappearing rapidly in communities where Roman Catholicism is being supplanted by evangelical Protestantism. We were originally drawn to this area by nomenclatural data in Alcorn’s Teenek ethnobotanical treatise [82] that associate cycads with the maize deity Dhipak (“Thipaak”) [83].

**Fig. 33:**
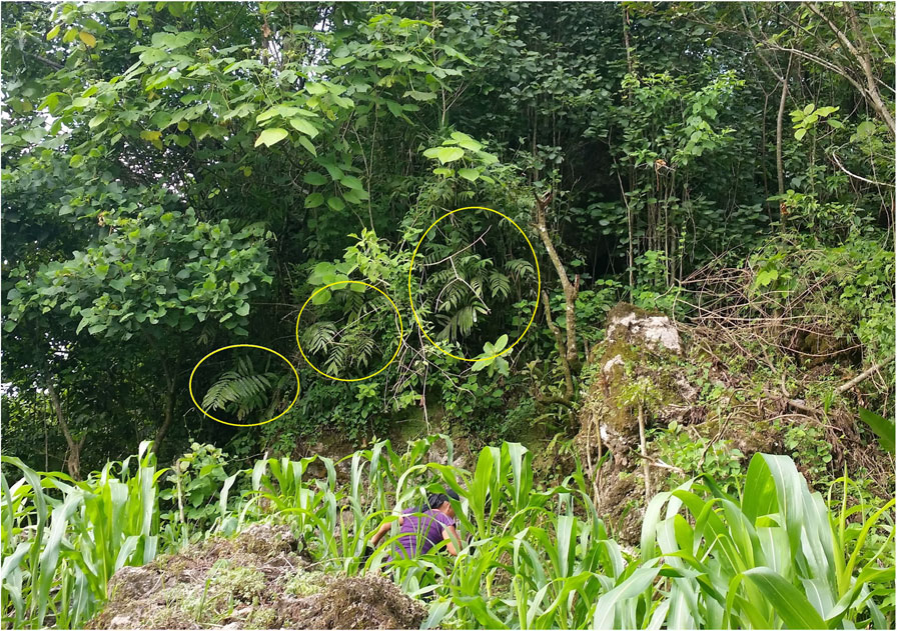
A Sierra Teenek *milpa* (maize field in swidden rotation) with protected *konlif* (*Ceratozamia latifolia*, highlighted) along the edge to protect and nurture the maize, 2016. *Konlif* seedlings found among the maize plants were also protected from weeding

While the *tzamaal* (*Dioon edule*) also has a certain importance to the Teenek, including the negative food associations detailed above, it is *konlif* (with several variations in pronunciation) that embodies certain core cultural beliefs and values, not least as an embodiment of Dhipak himself (Fig. 33). *Konlif* is perceived as the spirit of the *milpa*, which is, as Alcorn [82] explains and we corroborated in our research, a fluid process rather than a fixed spatial entity—i.e., the Teenek milpa is not directly equivalent to “cornfield.” As interviewees explained to us, the *milpa* is simultaneously the forest itself, the transformation of the forest to crops, with maize at the center, the return of the field to fallow and tall forest, and the repetitions of the cycle as practiced through fire-fallow (“swidden” or “slash-and-burn”) agriculture.^6^

We found that the role of *konlif* in the *milpa* is perceived as highly significant, deeply influential, and unique. *Konlif* is left where it occurs inside and around the periphery of *milpas*, protecting and strengthening maize and drawing moisture from the clouds. An account from Tancuime, Aquismon (translated from Spanish and Teenek) combines Roman Catholic and Mesoamerican conceptions of *konlif* and highlights its water-attracting properties:

Konlif. Friend of Thipak because in the old days it was like a maize cob, this is the maize cob from the old days, and it has stayed around so we could see that it was eaten. The [first] people in the old days did not eat anything, but when God’s people disappeared, the [next] people knew to eat the food of today. The konlif is not planted, it just grows from the earth, it is a maize cob from the old days. This maize’s spirit was inside a white crystal, and the people would take the crystal to the milpa, and when they were thirsty they put it in the milpa and when they were thirsty they put it in the soil, and before they knew it there was water, so then the men planted these old-time maize cobs. In the old days, God saw that the people did not eat this food and because of this God took it away. In our time God gave us maize and beans, but God gave the [konlif] crystal to men so that they could survive. [orig. at 44: 127].^7^

In one of the only cases we found of ethno-entomological knowledge of MNCA cycads, *konlif* in the *milpa* is known to attract butterfly larvae that eat its leaves, their frass fertilizing the soil. Meanwhile, the sarcotestas of fruiting *konlif* attract animal pests that eat this rather than maize.

Both in some of the stories about Dhipak—a deity in the form of a boy who first brought maize to people—and the origins of maize, and as a demonstration of the ongoing connections between maize and cycads and the ability of cycads to protect and benefit the maize crop, one can find *konlif* spoken of as Dhipak himself, as well as an embodiment of Dhipak’s evil grandmother or another adversary (this dual nature may also be related to the same phenomenon described under the section on cycads and palms, above, and potentially signifies post-colonization Roman Catholic influences on Indigenous conceptions).

In general terms, *konlif* as a plant with a soul is seen as the ancestor of maize or what people ate before maize, as well as a type of maize and a guardian of maize. One knowledgeable elderly female informant explained in detail the ways that the maize plant, which is both female and male, is a result of the fertilization of the female cycad cone by the male cycad cone. In her account, the male cone was conceptualized as a phallus and cycad pollen was equated to semen, while the female cone that opens to receive the pollen was conceptualized as a human-like reproductive apparatus. Maize was thus at some point in the past a creation of this union of dioecious cycad progenitors fused into a single monoecious plant.

**Fig. 34:**
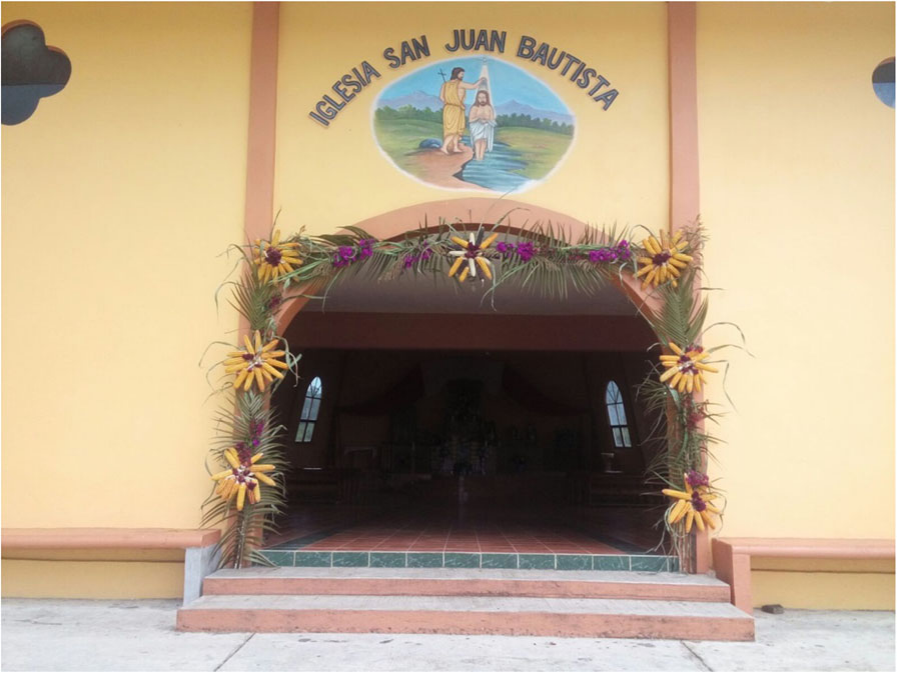
Maize cobs and *teocintle* (*Ceratozamia fuscoviridis*) leaf decorations on church, San Juan, Huazalingo, Hidalgo, 2016. Michaelmas (Día de San Miguel) coincides with the El Chicomexochitl maize-cycad deity festival. Photo by TD

**Fig. 35:**
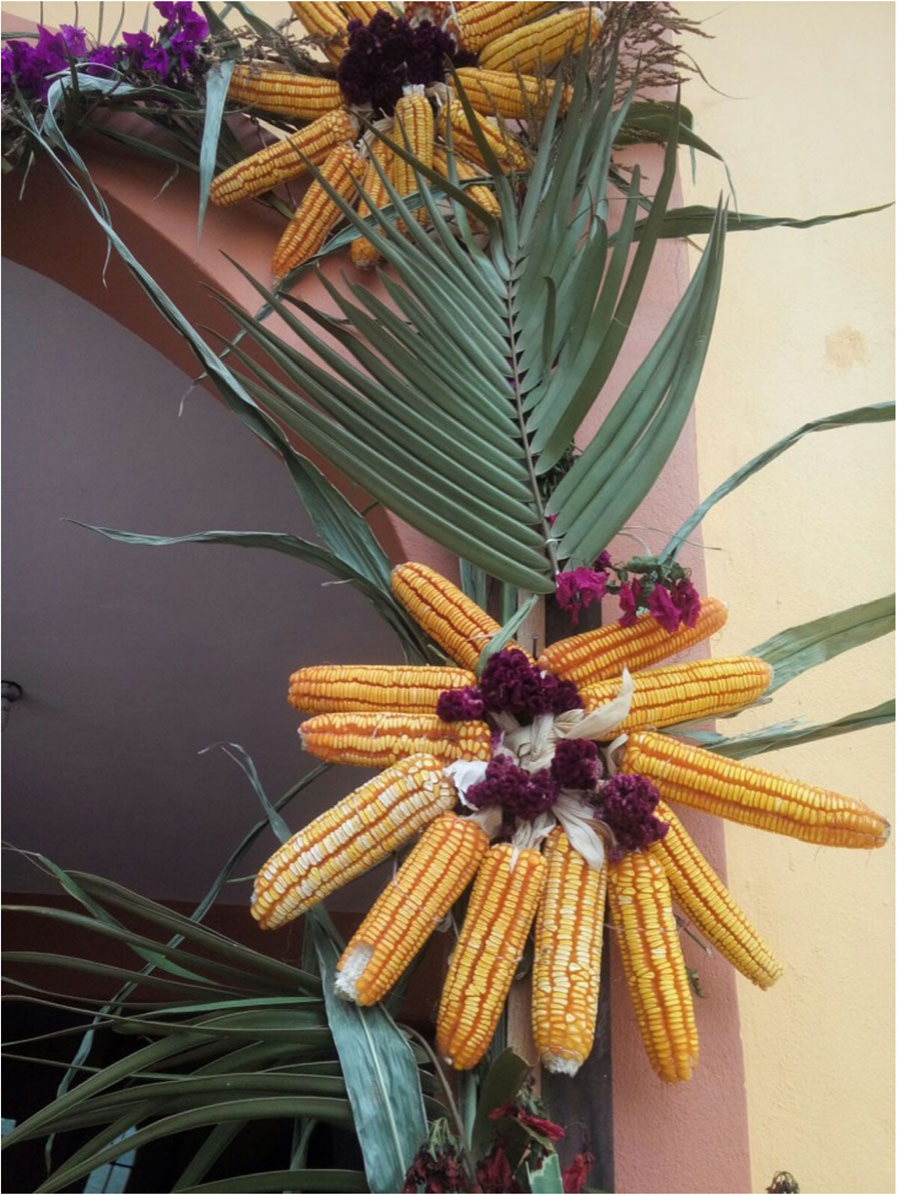
Close-up of Huazalingo church doorway. Maize cobs and leaves and cycad leaves are complemented by *bugambilia* (*Bouganvillia* sp., Nyctaginaceae), an introduced species. Photo by TD

**Fig. 36:**
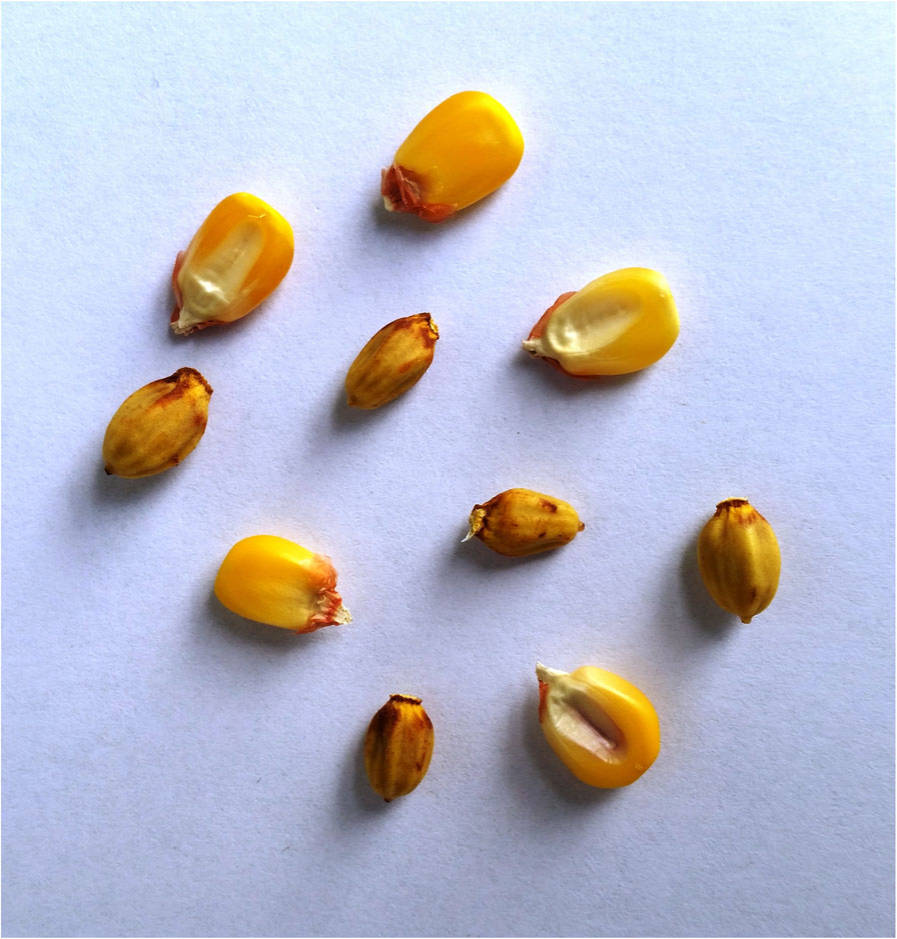
Striking similarity between *Zea mays mays* kernels and aborted *Dioon edule ovules*

**Fig. 37:**
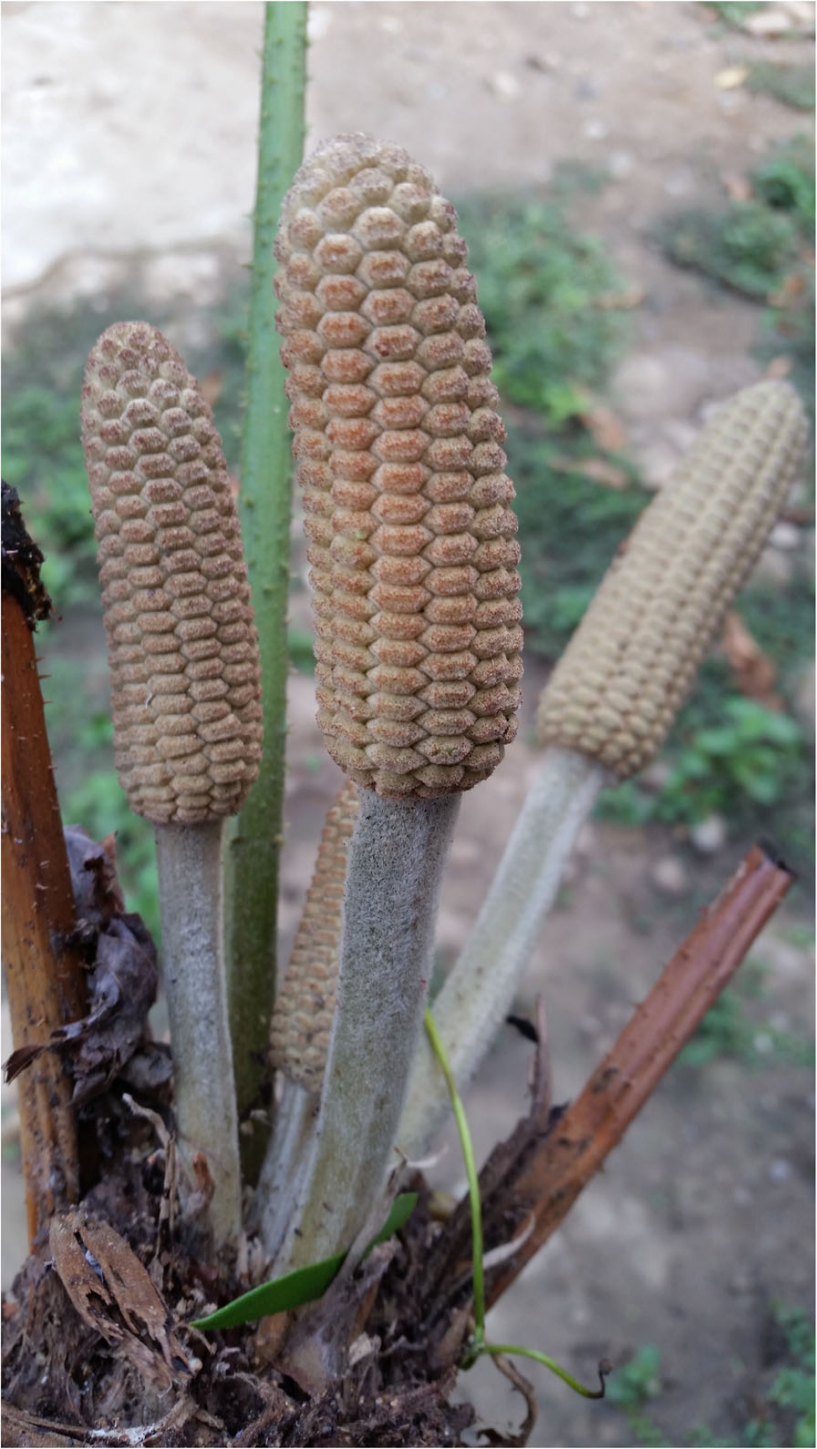
Characteristics of the local maize-cycad deity *Chicomesintli* (seven-maize-ear) or *chicomexochitl* (seven-flower) exemplified by multiple *Zamia loddigesii* male cones on a single plant. Tohuaco II, Huautla, Hidalgo, 2016

To the Nahua of the northern Hidalgo Huasteca region, the name *teocintle* signifies a sacred or ancestral ear of maize (*cintli*) (Figs. 34, 35, 36, and 37). That and several related names reflect a still-current belief that *Z*. *loddigesii* and *C*. *fuscoviridis* (and rarer cycads to a lesser extent) are ancestral to maize, in a fashion similar to Teenek beliefs. The following account is from a community that associates itself with the origin of maize from teocintle (*C*. *fuscoviridis*); see Figs. 34, 35, and 36.

Teocintle and the origin of maize: an account from San Juan Huazalingo, Hidalgo (translated from Spanish and Nahua)

In the days before the arrival of the Spanish, people ate teocintle, but they say that maize was found inside it, but they say that they found maize seeds inside it, instead of [cycad] seeds, and it was said that these were very different from what they had seen before, so they planted them and very beautiful maize seedlings sprouted, and from these many [more were born], but it is the teocintle that in Nahua mean maize’s uncle, and so from this teocintle from here maize was born. They say that the Aztecs ate this and thus they took it from here [Huazalingo]. [orig. at 44:127].

Another name for *teocintle* is Chicomexochitl (Seven-Flower), a local maize deity somewhat interchangeable with Dhipak, and celebrated as such in local maize ceremonies. The name “seven flower,” applied to maize ancestor/deity cycads, is a direct reference to the numerous cycad cones or “flowers” (*flores*) that emerge on stalks from subterranean stems [44]. *Teocintles* are purposely protected in communal *milpas* (conceived in the cyclical fashion described for the Teenek, above). While they are slashed and burned away with other wild vegetation when woods are cleared to create cropland, this is because it is known that they will quickly re-sprout and cone. When re-sprouted *teocintles* grow among the new maize plants, they are thought to confer size and resistance (e.g., against drought and pests) to maize [44]. They are also able to mystically hybridize with maize, producing the multi-colored grains in maize ears [38]. As with *konlif*, *teocintles* are identified as either maize ancestors, companions to maize, or types of maize, and seen as unique and distinct from all other plants in these roles. Overall, they are thought of as the “power” or energy guiding the *milpa* in a way roughly similar to that of *konlif* in Teenek conceptions.

### Cycads’ effects on climate, sense of place, and cultural identity

As well as the above roles of cycads in *milpa* agriculture, we encountered examples in a few other significant categories of strong relationships between cycads and people occurring at the scale of entire landscapes, municipalities, and cultures (“Other uses” column, Additional file 1: Table S1). As with cycads in Mesoamerican indigenous *milpas*, these point to deep connections highlighting cultural values for cycads that can be applicable to biocultural conservation schemes concerned with incorporating elements of pride in place and historical traditions in protection of biodiversity.

**Fig. 38:**
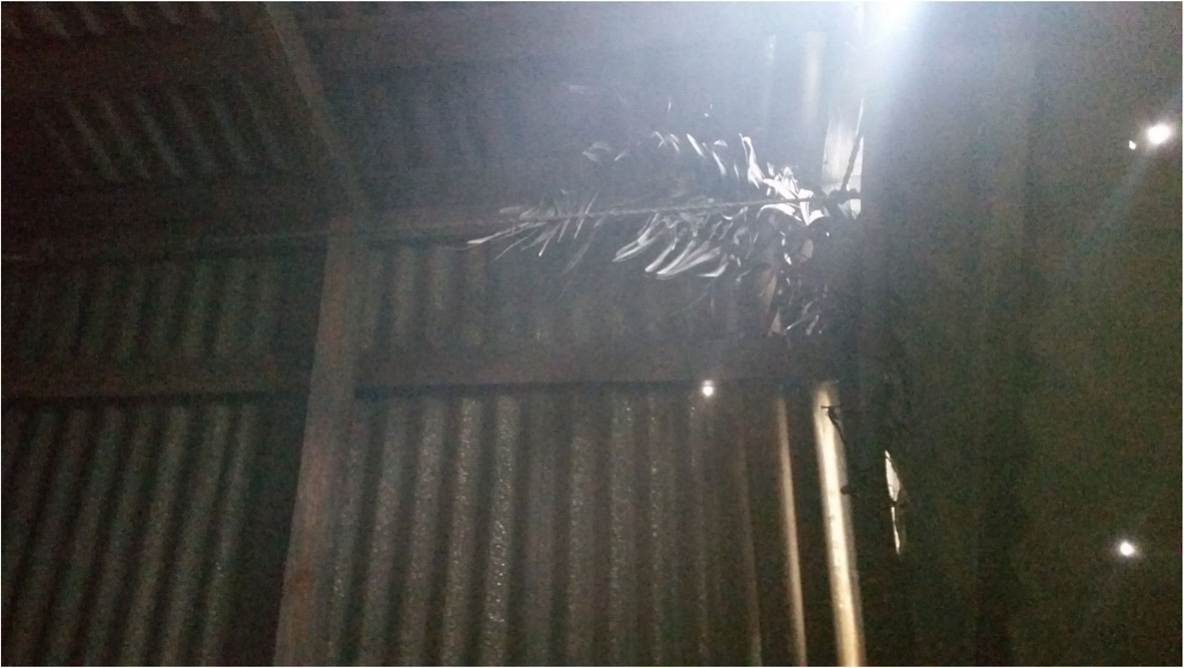
Previous year’s *xachitza* (*palma bendita*, *Ceratozamia fuscoviridis*) leaf, blessed during Palm Sunday, tucked under the roof to protect a house from storms in the Sierra Otomí-Tepehua, Hidalgo, 2016

In the case of influence on climate, while the maturation of cycad cones and the onset of the harvest are typically associated with the dry season, cycads are not seen in these contexts as having agency per se in weather events. Among the Teenek, however, as mentioned above, cycads are often said to “pull down” moisture from the clouds to the *milpa*. They are thus associated with the god of thunder and with bad weather. Similarly, among the Otomí-Ñuhu of Hidalgo state, cycad leaves, after having been blessed on Palm Sunday, are inserted into the eaves of houses to protect against lightning, while simultaneously seen as having the power to attract rain (Fig. 38).

**Fig. 39:**
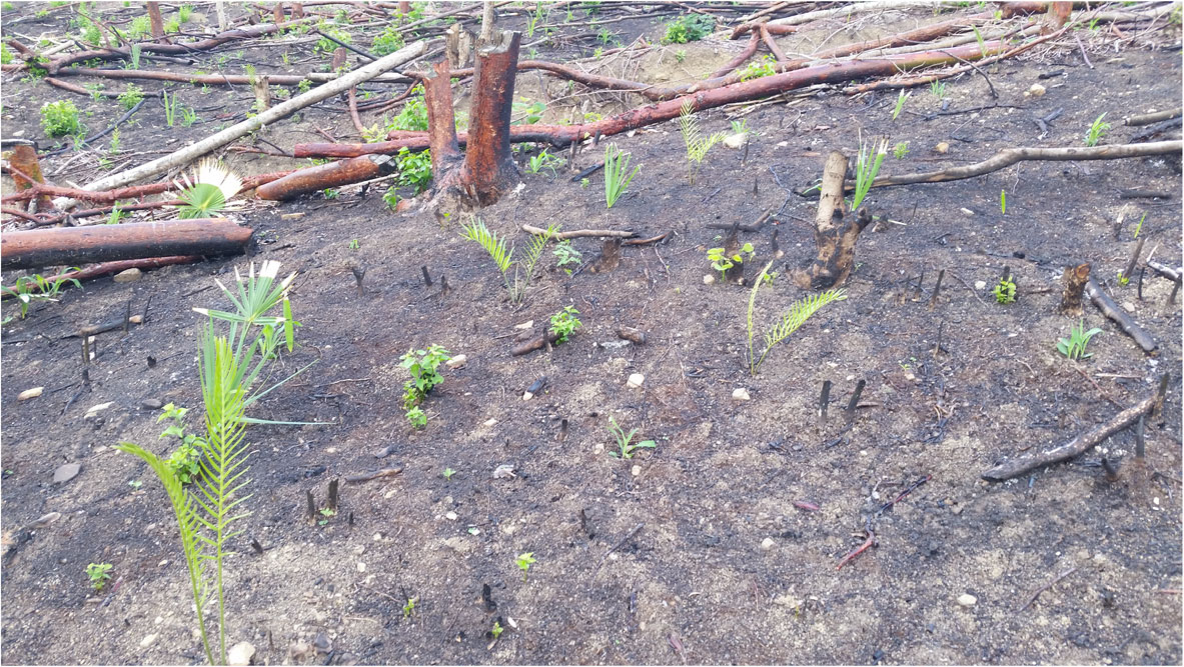
*Teocintle* (*Zamia loddigesii*) leaves sprout from underground stems in a fallow, burned-over *milpa* (maize field in swidden rotation), Tohuaco II, Huautla, Hidalgo, 2016. They are left unharmed when maize is planted, as it is believed that they confer various powers to maize, particularly overall hardiness (e.g., pest resistance) and superior size of the ears

How do cycads figure into the feelings people have for places? We follow the classic definitions of “sense of place” found in Bachelard [84] and Tuan [85] that have spread from geography and philosophy widely throughout numerous disciplines. In these definitions, people imbue places with positive meanings that accrue over time and that favor feelings of attachment, belonging, and harmony (or in some cases, neutral or negative associations such as detachment, lack of belonging, fear, etc.). Physical and biological features of landscapes such as mountains, rivers, caves, plants, and animals, are tangible contributors to sense of place. MB’s training as a geographer attuned him to the contribution of senses of place, both positive and negative, to the success or failure of cultural preservation and community-based conservation efforts. In the places where MNCA cycads are highly valued for food or in non-food maize-cycad *milpa* associations, the values of cycads, though rapidly becoming limited and forgotten, reach the status of extraordinary plants that define the value of places. A prime example is in cycad-focused Esquipulas del Norte, Honduras, where it is said that visitors who consume *tiusintes* there will either never leave or will always return. All social classes consume cycads in this municipality, and the plant is perceived as one of the fundamental elements of *patrimonio* (cultural heritage) that delineate the importance of place and place-based Esquipulas identity. In general, mestizo, and to a lesser extent, Indigenous people more widely in the Honduran *tiusinte* region associate intangible but very important non-monetary values of the landscape with the *tiusinte* populations that exist in them and the cycle of harvesting, consumption, and ritual based on them (Fig. 39).

The same can be said for the value of *chamal*, as *dameu*, among first-language Xi’iuy speakers [86], and less so, but still very tangible and of central importance, among X’iuy who no longer speak the language [72]. For non-food users, we recognize the Teenek, Nahua, and Chiapanec to value cycads as integral and important elements of places, as discussed above.

## Discussion

### Cycad contribution to human diets in view of their toxicity: revisiting the cycad hypothesis

It is clear that cycads have been, and in some cases continue to be, significant elements in local diets across parts of MNCA. What, exactly, their contributions are or have been in terms of nutritional value, beyond being at times a crucial source of carbohydrates, is less clear. Assuming cycads were far more widespread in the past, the large numbers of plants with female cones in any given year would have surpassed the needs of human populations in certain contexts. Among the small, mobile human populations of the earlier Holocene who first came into contact with them, and in particular with *Dioon* populations, cycads would have been extremely valuable food resources, even if only non-toxic sarcotestas were consumed. Data from the Sierra de Tamaulipas of 4000 years ago suggest that cycads may have attained dietary importance as great as they did in the Greater Antilles and Australia.

This is congruent with the lack of an association of cycads with long-term neurological effects. In the 411 interviews we conducted, informants were always forthcoming if they had heard about or witnessed acute cycad poisoning of people and animals, but no knowledge of any long-term neurological disorders was ever evinced. Previous data for *tiusinte* in Honduras [52] as well as data from Australia, Colombia, and the Caribbean also do not include any cases or knowledge of long-term neurological effects. This supports Steele and McGeer’s findings from Guam [14], where the cycad hypothesis originated, who after years of research concluded that there was simply no link between cycads and ALS-PDC or any other neurological disease.

### Origins of the cycad-maize relationship

We have failed to find an equivalent in the literature, wherein similar-looking wild plants are conceived of as ancestors to major domesticated plants, while having no close genetic relationship. The situation appears to be unique to maize and cycads, and it raises questions about the origin and logic of this association. Is it rooted in simple appearances, or derived from deep associations that could stretch back as far as the complementarity of the two foods in ancient hunter gatherer cultures, as evidenced in rock shelter middens? What are the benefits of associating the two plants? Addressing these questions is complicated because maize’s genetic ancestor *Zea mays parviglumis*, and its relatives in MNCA—other Poaceae grasses in the *Zea*, *Tripsacum*, and *Setaria* genera—are also understood in certain local cultures to be in some way related to maize, and are sometimes also known as *teocintle*. In Mexico, the term *teocintle* is traditionally only used for cycads (exclusively in NEMX). As applied to maize’s genetic relatives, it is of recent (nineteenth century) introduction to the country and derives from a Pipil (northern Central American Nahua-speaking) term [87].^8^ Thus, in reality, if terminology and nomenclature are any indication, there is a deep relationship between maize and maize relatives on one hand, and cycads on the other, as members of a single conceptual group. But where did such a concept, found from one extreme of the Atlantic slope of Mesoamerica to the other, originate?

It is possible that the conception of cycads as maize ancestors derived from NEMX, where it is still very much in evidence, and was spread southward by highly mobile Nahua-speaking people as far as northeastern Honduras, where Nahua trading colonies existed in the *tiusinte* region and were documented by Hernando Cortés in the early 1500s [88]. The arrival of only a handful of NEMX families sometime during the last millennium with memories of this specialized knowledge might explain the relatively impoverished religious connections between cycads and maize found among Nahoa and mestizos in Honduras in comparison to the rich traditions found among the Nahua of Hidalgo. However, considerations of religious significance and nomenclature aside, the greatest similarities in Honduran and Mexican cultural practices are between the *tiusinte* region and Xi’iuy treatment of cycads rather than Teenek or Nahua treatment. It is difficult to believe such similarities could have arisen separately given that they involve identical detoxification practices (particularly ash-washing), cultural beliefs (what constitutes a good tamale), and other aspects of food preparation specific to the two cultures while distinct from anything in the intervening region. However, there is no record of Xi’iuy people migrating to Honduras. The key to explaining this conundrum is the multicultural nature of the broader Huasteca region of NEMX through time. For many centuries, as they do today, Nahua, Teenek, and Xi’iuy lived in close proximity and sometimes intermarried, thus explaining how a small number of mixed Nahua-Xi’iuy families migrating to Honduras—any time before the 1700s, when Mexican terms for cycads are first picked up in the historical record in Honduras, and after around 1000 AD—could have transported a limited but diverse suite of cultural beliefs and practices involving cycads and preserved it in an isolated part of Central America among an Indigenous culture that became known as *Nahoa de Honduras* [69], from which neighboring mestizos are also derived.

Indeed, the Xi’iuy and similar originally hunter gatherer groups whose ancestors used cycads heavily in the northeastern Mesoamerican frontier region of NEMX in association with maize are intriguing candidates for the ultimate origin of the maize-cycad relationship. However, given our lack of understanding of whether the spatial maize-cycad relationship over four millennia in strata excavated at La Perra reflected any meaningful sacred relationships between the two plants—as we find today among the Nahua and Teenek—or were simply coincidence, we cannot discard alternate possibilities. It is fully possible that the association originated outside of NEMX in places like Oaxaca, where it also exists, or even in WMX, for example in the maize-origin Balsas River drainage area of Guerrero where both *Dioon stevensonii* and *Zamia paucijuga* are still found.^9^

What are the agricultural benefits for the maintenance of cycads in *milpas* that would support a long-term association? Some avenues to explore include the nitrogen-fixing characteristics of cycads and other effects on the soil and rhizosphere that may make them ideal for strengthening and improving maize crops. It is certainly possible that proto-agriculturists sought out cycad populations to plant maize in for reasons such as this. The ultimate origins of and reasons for the maize-cycad belief system, and their connection to the original teosinte-as-grass region in Central America, remain fascinating questions to follow up on.

### Threats to the continuance of ceremonial use

As mentioned in the section on religion, above, sacred uses and knowledges of cycads are paramount for biocultural conservation strategies. When cycads are included in floral arrays, these frequently incorporate other plants of great religious importance such as *sotol* (*Dasilyrion* spp.) and *cempasuchil* (*Tagetes erecta*, marigold). In addition to the economic importance and effect on wild plants that such uses may have, it is important to note that the intricate practice of floral arranging is being eroded. Where reasons for incorporation of cycad leaves are forgotten or no longer deemed important, substitutes are found, such as plastic leaves for Honduran Day of the Dead wreathes [52]. Floral arrangement practice at its most complex includes knowledge of wild populations as well as the preparation of a range of decorations, the placement of such decorations, and the function of the decorations in each ceremony. This knowledge is passed down through oral tradition via guild-like associations of participants. The dimensions of these traditions for cycads have not yet been studied through proper documentation (preferably via video and photography) that should occur during the events themselves. In almost all cases, we have been limited to observing decorations left up after events, asking interviewees how such events take place and how decorations are created. The connections between such ritual decorative uses, sacramental food, and other components noted in this article should be studied further.

### Limitations of the study

We were greatly limited by the extremely fragmentary nature of published cycad ethnobotanical data prior to our work. Researchers with no prior knowledge of the unique importance of cycads in local culture have failed to ask important questions such as those about the roles of *konlif* in Teenek *milpas*. It is our experience that interviewees do not discuss plants of such importance casually and are more used to being asked about plants outsiders perceive, and the literature underscores, are the most important: first maize, second other crops, and third, wild plants, with least importance afforded those wild plants that available literature suggest are toxic and therefore of little importance.

Another limitation for us was the growing realization that local people withhold or talk little about cycad knowledge because of its sacred, protected nature. This case is similar to accounts elsewhere in the world of numerous important plants, suggesting that a primary reason for cycads’ remaining unknown is such qualities, tied, we suspect, to their shamanistic uses as entheogens [58, 89].

Our archeological data are sparse because MNCA archeologists have not looked specifically for evidence of cycads using methods such as pollen or starch grain analysis—indeed, no index of *Dioon* or *Ceratozamia* pollen or starch grains exists that could be employed, suggesting that cycad remains may have been overlooked in past excavations. Compare this to the greater Caribbean, where *Zamia* is considered to have been a staple since earliest human settlement, and archeobotanical remains of *Zamia* are typically prominent in the archeological record e.g.,[90]. The same should be the case for numerous MNCA cycads.

More than anything, we were limited by the vast geographical scope of the research, and biased toward areas most easily accessible to our research institutions. Given the fragmentary knowledge of this field of inquiry prior to the onset of our research, we were eventually able to develop adequate questionnaires (Additional file 2) as we gained field experience learning of many beliefs about and uses of cycads that had never been recorded previously. This was additionally challenging because cycads have been incompletely surveyed, particularly in WMX and Oaxaca, where prominent populations of *Dioon* and *Ceratozamia* have still not been taxonomically treated by botanists.^10^

Overall, it can safely be said that our study only delved into cycad ethnobotany in depth in Honduras and among the Teenek, Nahua, and certain mestizo communities of NEMX, while Xi’iuy cycad ethnobotany has been adequately investigated by others. Otherwise, extensive work remains to be done for numerous other ethnic groups we are aware of that possess ethnobotanical knowledge of cycads.

## Conclusions

While we have advanced considerably in providing at least partial answers to our research questions, let us examine whether our research to date has allowed us to confirm our hypotheses. In terms of food, we can state that while they were clearly once important, cycads have lost enormous value across MNCA except in isolated areas, and even in those areas, food use is diminishing as knowledge of detoxification procedures are lost and former consumers turn to easier-to-prepare and less toxic food sources. Nevertheless, long-term neurotoxic effects indeed have not been an issue, as we suspected. However, we recommend controlled studies of cycad-consuming populations to completely rule this out, and to test for potential long-term negative health effects of other types (e.g., on the liver) as well as for positive effects, given that we know very little about the nutritional value of cycads apart from the carbohydrate content.

The use of cycads in religion appears to be the primary reason people are aware of the plants and appreciative of their values in the vast amount of cases where cycads are not eaten. In terms of incorporation into religious ceremonies, our data do not indicate any increasing or decreasing trends, but they definitely show the centrality of cycads in a range of practices the extent and significance of which have barely been studied. We should also point out that whether or not they are perceived as palms, cycads are specifically sought out for their intrinsic qualities and imbued highly distinct meanings as cycads, with both positive and negative effects on wild populations.

We additionally hypothesized that awareness of how cycads contribute to sense of place and cultural identity could in turn stimulate traditional protection measures designed to help preserve cycads. This was obviously borne out in the cited cases of *tiusinte* in Honduras and Chiapanec pilgrimages, as well as in manifestations of the cycad-maize relationship in NEMX. Overall, though, despite a few cases to the contrary, we were vindicated in our claim that cycad ethnobotanical knowledge, uses, and traditional protection measures are being rapidly eroded across the entire region. We hope that this article will contribute to a reversal of the trend.

We have uncovered an obvious connection between importance of cycads to local culture and how local cultures treat wild populations, and this is key to converting the findings in this study to useful elements of community-based conservation. Cultures that value cycads to a great extent appear to be more likely to avoid eliminating them in the wild, but as such appreciation fades, cycads come to be ignored and are left to be eradicated. But even the worst cases, such as the wholesale destruction of cycads for cattle pasture, have counter examples such as the co-management of cattle and cycads in the Pamería region and the contemporary focus in the Honduran *tiusinte* region on creating compatibility between cycads and cattle ranchers [91]. Numerous other elements of cycad ethnobotanical knowledge and practice exist that should be publicized and interpreted in collaboration with communities, as has already been done with *Dioon merolae* in Chiapas and *Dioon mejiae* in Honduras. While cycads can become sustainable trade items for purely decorative reasons that put money in harvesters’ and growers’ pockets, we suggest that it is most likely they will be protected in situ by local people in the long run if the incentive for their protection is as key elements in the preservation of local identities, traditions, cultural heritage, and sense of place. These, after all, not pecuniary motives, are the reasons they have been protected and valued already for millennia.

Models of community-based cycad conservation also have validity beyond MNCA, given the commonalities found among human-cycad relationships around the world. This study is intended to inspire similar efforts elsewhere as a step toward new approaches to cycad conservation that, where possible, are able to draw on old and even discarded beliefs and traditions.

## Additional files

Additional file 1: **Table S1.** Ethnobotany of MNCA Cycads by Local Term. Contains tabulated qualitative ethnographic data arranged in columns by category. Entries are alphabetized by Local Term. **Table S2.** Ethnobotany of MNCA Cycads by Species. Distillation and reorganization of Supplementary Table 1 indicating presence (x) or absence of various ethnobotanical uses, organized by species (alphabetized by Latin name) (DOCX 161 kb)

Additional file 2: Ethnographic Methods and Questions for Cycad Research. Contains two documents. ‘Ethnobotanical research on cycads in Mexico: Methods and suggested topics’ (Notes for field assistants and collaborators prepared by Mark Bonta, 2008–2014) and ‘Cycad Ethnobotany Questionnaire’ (Aurelia Vite Reyes [45]). (DOCX 47 kb)

## Abbreviations

AC: Angélica Cibrián-Jaramillo
AR: Aurelia Vite-Reyes
AV: Andrew Vovides
FPIC: Free, Prior, and Informed Consent
MB: Mark Bonta
MNCA: Mexico and northern Central America (Honduras, Guatemala, Belize, El Salvador)
NEMX: Northeastern and North-central Mexico
SMX: Southern Mexico
TD: Teresa Diego Vargas
TP: María Teresa Pulido
WMX: Western Mexico

## Footnotes

1 With the exception of *Dioon caputoi*, found at ca. 2000 m above sea level in a semi-arid area receiving around 300 mm annual precipitation [92].

2 To date, we have not unequivocally identified cycads in pre-Columbian iconography (mural paintings, ceramic designs, lithic carvings, etc.), but given the interchangeability of cycads and maize in cultures such as Nahua and Teenek (one seen as a type of the other, both imbued with deep sacred meanings, and cycads as embodiments of the maize deities Chicomexochitl and Dhipaak), following Carrasco [93], we suggest that iconographic representations from the region that are presumed to be of maize might need to be reexamined.

3 Perplexingly, Hernandez’s extant works do not mention *chamal*, a widely known cycad appearing often in later accounts, probably related to the fact that many of his botanical data were destroyed in a fire.

4 In the Caribbean and Florida, *Zamia* stem starch was one of the most important early staple foods and the plant was cultivated and manipulated to a certain extent for this purpose [94, 95]. Seeds from large *Zamia* cones serve as a source of the tamales known as *envuelto de chigua* in Pacific Colombia [96].

5 We have also gathered references to mestizo, Zoque, and Zapotec cycad pilgrimages in the general region of the Chiapanec pilgrimages, the Isthmus of Tehuantepec.

6 The fluidity and cyclical nature of the *milpa* that contains many plants and plant-animal-landscape interactions embodying deities and cosmic forces is a feature of Indigenous cultures throughout Mesoamerica.

7 The temporality of this account is confusing in translation, but it is referring to previous eras—the first during which people did not eat food at all, the second during which they learned to eat ancestral food such as cycads, and the present time, when they learned to eat modern food that was derived from ancient food. Teenek accounts of modern food mostly indicate that the ancestral foods were abandoned, but sometimes refer to the ancestral food as also having been eaten in modern times.

8 This became standard nomenclature in botany and agronomy [97] via the introduction of “teosinte” forage grasses into the global economy. Even in the Balsas River drainage basin of Guerrero, where maize originated as it was gradually domesticated by hunter gatherers from a wild relative [98], this wild relative, *Zea mays parviglumis*, is nowhere referred to in local culture as *teosinte* or anything similar [99].

9 Due to security concerns, we have not done ethnobotanical fieldwork in Guerrero and are unaware if either species is related in any way to maize in local culture.

10 A case that illustrates this is our trip to Jesus María, Nayarít to follow up on an herbarium voucher note that leaves of *Dioon tomasellii* had been found discarded at the church there [100]. No other botanical literature on cycads in the region existed. After arriving at what is the traditional administrative and cultural center for the Cora ethnic group, we soon established that numerous undocumented cycad populations existed in the surrounding mountains and that the *turhaa* cycad was quite important in religious ceremonies. We found its cone carved into the colonial-era church altar and a colonial-era statue of a saint clutching a cycad leaf. Such discoveries of cycad populations unknown to biologists are not uncommon in cycad ethnobotany, and point to the vast amount of work yet to come.

## References

[1] Nagalingum NS, Marshall CR, Quental TB, Rai HS, Little DP, Mathews S. Recent synchronous radiation of a living fossil. Science. 2011;334:796–9. 10.1126/science.1209926 22021670

[2] Calonje M, Stevenson DW, Stanberg L (2018). The world list of cycads, online edition.

[3] Donaldson JS (2003). Cycads: status survey and conservation action plan. IUCN--the World Conservation Union.

[4] Vovides AP (1989). Problems of endangered species conservation in Mexico: cycads an example. Encephalartos.

[5] Vovides AP, Pérez-Farrera MA, Iglesias C (2010). Cycad propagation by rural nurseries in Mexico as an alternative conservation strategy: 20 years on. Kew Bull.

[6] Ruiz-Mallén I, Schunko C, Corbera E, Rös M, Reyes-García V (2015). Meanings, drivers, and motivations for community-based conservation in Latin America. Ecol Soc.

[7] Graham D, Bonta M, Ulloa R (2011). Cycad conservation, peasant subsistence, and the military coup in Honduras. Soc Nat Res.

[8] Pérez-Farrera MA, Vovides AP. The ceremonial use of the threatened “espadaña” cycad (Dioon merolae, Zamiaceae) by a community of the central depression of Chiapas. Bol Soc Bot Mex. 2006;78:107–13.

[9] Chamberlain CJ (1919). The living cycads.

[10] Norstog KJ, Nicholls TJ. The biology of the cycads. Ithaca: Cornell UP; 1997.

[11] Wauchope R, Kislak JI. Handbook of Middle American Indians, vol. 8. Austin: U Texas P; 1964.

[12] MacNeish RS. Preliminary archaeological investigations in the Sierra de Tamaulipas, Mexico. Trans Am Phil Soc. 1958;48:1–210.

[13] Whiting MG. Food practices in ALS foci in Japan, the Marianas, and New Guinea. In: Proc 3^rd^ Conf Toxicity of Cycads. Fed Proc. 1964;23:1343–5. 14236144

[14] Steele JC, McGeer PL (2008). The ALS/PDC syndrome of Guam and the cycad hypothesis. Neurology.

[15] Whiting MG (1963). Toxicity of cycads. Econ Bot.

[16] Cox PA, Sacks OW (2002). Cycad neurotoxins, consumption of flying foxes, and ALS-PDC disease in Guam. Neurology.

[17] Thieret JW (1958). Economic botany of the cycads. Econ Bot.

[18] Smith M (1982). Late Pleistocene Zamia exploitation in southern Western Australia. Arch Oceania.

[19] Smith CE (1967). Plant remains. In: MacNeish RS, editor. Prehistory of the Tehuacan Valley. Vol.1: environment and subsistence.

[20] Patiño VM (1989). Notas preliminares sobre el uso de las Zamiaceas por los pueblos primitivos y aculturados del intertrópico americano. Perez-Arbelaezia.

[21] Smith HG (1951). 1951. The ethnological and archeological significance of Zamia. Am Anthropol.

[22] Bonta M, Osborne R. Cycads in the vernacular—a compendium of local names. In: Vovides AP, Stevenson DW, Osborne R, editors. Proceedings of the seventh international conference on cycad biology (Xalapa, Mexico, 2005). Mem New York Botan G; 2007. p. 143–75.

[23] Radha P, Singh R (2008). Ethnobotany and conservation status of Indian Cycas species. Encephalartos..

[24] Singh KJ, Singh R. The ethnobotany of Cycas in the states of Assam and Meghalaya, India. In: Stevenson DW, Osborne R, Taylor A, editors. Proceedings of Cycad 2008: the 8th International Congress on Cycad Biology. Mem New York Botan G; 2012. p. 151–64.

[25] Beck W (1992). Aboriginal preparation of Cycas seeds in Australia. Econ Bot.

[26] Bradley JJ (2005). ‘Same time poison, same time good tucker’: the cycad palm in the south west gulf of Carpentaria. Journ Austral Stud.

[27] Beaton J. Dangerous harvest [dissertation]. Canberra: Austral Nat U; 1977.

[28] Bonta M. Cycads and human life cycles: outline of a symbology. In: Stevenson DW, Osborne R, Taylor A, editors. Proceedings of Cycad 2008: the 8th International Congress on Cycad Biology. Mem New York Botan G; 2012. p. 133–50.

[29] Keppel G (2009). Morphological variation, an expanded description and ethnobotanical evaluation of Cycas seemannii A. Braun (Cycadaceae). S Pac Journ Nat Appl Sci.

[30] Vovides AP (1990). Spatial distribution, survival, and fecundity of Dioon edule (Zamiaceae) in a tropical deciduous forest in Veracruz, Mexico, with notes on its habitat. Am J Bot.

[31] Vovides AP. Flora de Veracruz Fasciculo 26, Zamiaceae. Xalapa, Veracruz: INIREB; 1983.

[32] Tang W (1987). Insect pollination in the cycad Zamia pumila (Zamiaceae). Am J Bot.

[33] Norstog KJ, Fawcett PK (1989). Insect-cycad symbiosis and its relation to the pollination of Zamia furfuracea (Zamiaceae) by Rhopalotria mollis (Curculionidae). Am J Bot.

[34] Norstog KJ (1990). Studies of cycad reproduction at Fairchild tropical garden. Mem New York Botan G.

[35] Vovides AP (1991). Insect symbionts of some Mexican cycads in their natural habitat. Biotropica.

[36] Sánchez-Tinoco MY, Engleman EM, Vovides AP (2000). Reproduction chronology of Ceratozamia mexicana (Cycadales). Botan Sci.

[37] Dehgan B, Yuen C (1983). Seed morphology in relation to dispersal, evolution, and propagation of Cycas L. Bot Gaz.

[38] Grove TS, O’Connell AM, Malajczuk N (1980). Effects of fire on the growth, nutrient content and rate of nitrogen fixation of the cycad Macrozamia riedlei. Austral J Bot.

[39] Yáñez EL (2006). Las cycadas: biología y conservación en México.

[40] Stresser-Péan G (2000). San Antonio Nogalar: La Sierra de Tamaulipas y la frontera noreste de Mesoamérica.

[41] Whitelock LM. The cycads. Portland, Oregon: Timber P; 2002.

[42] Martin GJ (2010). Ethnobotany: a methods manual.

[43] Alexiades MN, Sheldon JW. Selected guidelines for ethnobotanical research: a field manual. New York: New York Botan G; 1996.

[44] Diego-Vargas T. Relaciones culturales entre las cícadas y el maíz en localidades nahuas y teenek de la Huasteca [Master’s thesis]. Pachuca: Univ Aut Estado Hidalgo; 2017.

[45] Vite A. Etnobotánica de cícadas en comunidades nahuas y mestizas de Tlanchinol, Hidalgo [Master’s thesis]. Pachuca: Univ Aut Estado Hidalgo; 2012.

[46] Vite A. Etnobotánica de cícadas en Hidalgo y algunos aspectos demográficos de Ceratozamia fuscoviridis D. moore [Undergraduate thesis]. Pachuca: Univ Aut Estado Hidalgo; 2010.

[47] Newsom L, Hofman C (2008). Caribbean paleoethnobotany: present status and new horizons (understanding the evolution of an indigenous ethnobotany). Crossing the borders: new methods and techniques in the study of archaeological materials from the Caribbean.

[48] Mayett Moreno Y, Castañeda ES, Barajas DM (2014). Comercialización de cicadas mexicanas (Zamiaceae) en Atlixco, Puebla: un estudio exploratorio. Rev Mex Ciencias Agr.

[49] Pulido MT (2011). Informe de actividades, IV feria del teosinte 2011.

[50] Bonta M (2010). Maize and cycads: in search of sacred ancestors. The Cycad Newsletter.

[51] Bonta M. Ethnobotany of Honduran cycads. In: Vovides AP, Stevenson DW, Osborne R, editors. Proceedings of the seventh international conference on cycad biology (Xalapa, Mexico, 2005), vol. 97. Mem New York Botan G; 2007. p. 120–42.

[52] Bonta M, Flores Pinot O, Graham D, Haynes J, Sandoval G (2006). Ethnobotany and conservation of tiusinte (Dioon mejiae Standl. & L. O. Williams, Zamiaceae) in northeastern Honduras. J Ethnobiol.

[53] Flores JS, Narave H, Vovides AP. 1992. Etnoflora yucatanense: fasciculo 5. Gymnospermae: taxonomia y etnobotanica. Merida: Universidad Autónoma de Yucatán; 1992.

[54] Vovides AP. Flora del Bajio y de regiones adyacentes: Fasciculo 71, Zamiaceae. Xalapa, Veracruz: Instituto de Ecología; 1999.

[55] Vite-Reyes A, Pulido MT, Flores JC. Estrategia estatal de conservación de cícadas (Zamiaceae): una propuesta para el Estado de Hidalgo, México. Rev Biol Trop. 2013;61:1119–31. 10.15517/rbt.v61i3.11908. Accessed 14 Oct 2018. 24027912

[56] Pulido MT, Flores-Vazquez JC, Vite A, Vargas-Zenteno M, Vargas Roldan S, Piedra-Reynoso K, Octavio-Aguilar P, Vovides A, Ramírez-Bautista A, Sánchez-González A, Sánchez-Rojas G, Cuevas-Cardona MC (2017). Hidalgo: cuarto lugar en riqueza de cícadas en México. Biodiversidad del Estado de Hidalgo.

[57] Code of Ethics. International Society of Ethnobiology. http://www.ethnobiology.net/what-we-do/core-programs/ise-ethics-program/code-of-ethics/. Accessed 3 Nov 2018.

[58] Bonta MA, Bamigboye SO (2018). Use of cycads as ritual and recreational narcotics. S Afr J Bot.

[59] Hanselka JK. Prehistoric plant procurement, food production, and land use in southwestern Tamaulipas, Mexico [dissertation 128]. St Louis: Washington U; 2011.

[60] Hanselka JK. Revisiting the archaeobotanical record of Romero’s cave in the Ocampo region of Tamaulipas, Mexico J Ethnobiol. 2017;37:37–59.

[61] Simms SR (2014). Prehispanic Maya foodways: archaeological and microbotanical evidence from Escalera al Cielo, Yucatan, Mexico [dissertation ].

[62] Smith BD (2005). Reassessing Coxcatlan cave and the early history of domesticated plants in Mesoamerica. PNAS.

[63] Sifuentes de Ortiz MS (1983). Importancia economica del chamal Dioon edule Lindl. (Cycadaceae) en el estado de Nuevo Leon, México.

[64] Sahagún B (1577). *Historia general de las cosas de nueva España* (General history of the things of New Spain).

[65] Gates W. The de la Cruz-Badiano Aztec herbal of 1552. Baltimore: The Maya Society; 1939.

[66] Hernandez F (1946). Historia de las plantas de la Nueva Espana, tomo III, libro VI, chap. XLI, Del teocintli o tepecintli, o sea espiga montes de maiz.

[67] Berlandier JL, Chovel R. Diario de viage de la comisión de límites. Mexico City: Tip. J. R. Navarro; 1849.

[68] Lópe de la Cámara Alta A. Descripción general de la colonia de Nuevo Santander. Mexico City: UNAM; 2006. p. 2006.

[69] Bonta M, Urquijo Torres PS, Barrera-Bassols N (2009). The dilemma of indigenous identity construction: the case of the newly-recognized Nahoa of Olancho, Honduras. Temas de geografía latinoamericana, Reunión CLAG-Morelia.

[70] Whiting M, Spatz M, Matsumoto H (1966). Research progress on cycads. Econ Bot.

[71] Small JK (1921). Seminole bread—the conti, a history of the genus Zamia in Florida. Journ NY Botan Gard.

[72] Tristan ME. Aprovechamiento alimentario de Dioon edule Lindl. (chamal) en comunidades de la region Xi’iuy del estado de San Luis Potosí [undergraduate thesis]. San Luis Potosí: Univ Aut SLP; 2012.

[73] Chemín BH. Recetario pame de San Luís Potosí y Querétaro. Mexico City: CONACULTA, Culturas Populares; 2000.

[74] Reko VA (1949). Magische gifte: rausch-und betäubungsmittel der neuen welt.

[75] Vovides AP, Iglesias CG (1994). An integrated conservation strategy for the cycad Dioon edule Lindl. Biodivers Conserv.

[76] González-Coloma A, Reina M, Sáenz C, Lacret R, Ruiz-Mesia L, Arán VJ, Sanz J, Martínez-Díaz RA (2012). Antileishmanial, antitrypanosomal, and cytotoxic screening of ethnopharmacologically selected Peruvian plants. Parasitol Res.

[77] Schultes E. Peyote (Lophophora williamsii) and plants confused with it. Botan Mus Leaflets, Harvard Univ. 1937;5:61–88.

[78] Gutiérrez CG. Los antiguos Pames, historia oral de un pueblo. Morelia: Morevallado Ed; 2015.

[79] Galaviz de Capdevielle ME (1971). Descripción y pacificación de la Sierra Gorda. Estudios de historia novohispana.

[80] Valdez AU (2009). La flor de espadaña en Terán, ofrenda de los hojeros a la Santa Cruz.

[81] Socton R. La topada de los espadañeros en Suchiapa HD. Youtube; 2014. https://youtu.be/SCJ_yEebJRU. Accessed 20 Apr 2018.

[82] Alcorn J (1984). Huastec Mayan ethnobotany.

[83] Alcorn JB, Edmondson B, Hernández Vidales C, Staller JE, Tykot RH, Benz BF (2006). Thipaak and the origins of maize in northern Mesoamerica. Histories of maize: Multidisciplinary approaches to the prehistory, linguistics, biogeography, domestication, and evolution of maize, Chap. 43.

[84] Bachelard G (1994). The poetics of space.

[85] Tuan YF (1990). Topophilia: A study of environmental perceptions, attitudes, and values.

[86] Chemin Bassler H, Chamal EL, Valle J, Utrilla B, Prieto D (2012). Alimento divino de los Pames-Xi’iui de San Luis Potosí y Querétaro. Los pueblos indígenas de la Huasteca y el semidesierto queretano (Atlas etnográfico).

[87] Kempton JH, Popenoe W (1937). Teosinte in Guatemala. Report of an expedition to Guatemala, El Salvador, and Chiapas, Mexico. Contrib Am Arch.

[88] Cortés H (1963). Quinta carta relación de Hernán Cortés al Emperador Carlos V, Tenuxtitan, 3 de septiembre de 1526. Cartas y documentos.

[89] Dickau R, Redwood SD, Cooke RG (2013). A 4,000-year-old shaman’s stone cache at casita de Piedra, western Panama. Archaeol Anthrop Sci.

[90] Dickau R, Ranere AJ, Cooke RG (2007). Starch grain evidence for the preceramic dispersals of maize and root crops into tropical dry and humid forests of Panama. PNAS.

[91] Reyes W, Suazo AM (2007). Caracterización biofísica socioeconómica y ambiental de Saguay y Río Grande zonas de concertación de teocinte (*Dioon mejiae*).

[92] Said Gutiérrez-Ortega J, Yamamoto T, Vovides AP (2018). Aridification as a driver of biodiversity: a case study for the cycad genus *Dioon* (Zamiaceae). Ann Bot.

[93] Carrasco M. Cycads, maize, and garfish: the representation of ethnoecological systems in Olmec iconography. Schedule and Abstracts of the 10th International Conference on cycad biology (CYCAD 2015). Coral Gables: Cycad 2015 organizing committee, Montgomery Botanical Center; 2015. p. 18.

[94] Mickleburgh HL, Pagán-Jiménez JR (2012). New insights into the consumption of maize and other food plants in the pre-Columbian Caribbean from starch grains trapped in human dental calculus. Journ Arch Sci.

[95] Pagán Jiménez JR, Hofman CL, van Duijvenbode A (2011). Early phytocultural processes in the pre-colonial Antilles. Communities in contact: essays in archaeology, ethnohistory, and ethnography of the Amerindian circum-Caribbean.

[96] Patiño VM (2005). 2005. La alimentación en Colombia y en los países vecinos (Vol. 1).

[97] Wilkes HG (1967). Teosinte: the closest relative of maize.

[98] Matsuoka Y, Vigouroux Y, Goodman MM, Sanchez J, Buckler E, Doebley J (2002). A single domestication for maize shown by multilocus microsatellite genotyping. PNAS.

[99] Mondragón-Pichardo J, Vibrans H (2005). Ethnobotany of the balsas teosinte (Zea mays ssp. parviglumis). Maydica.

[100] McVaugh R. Flora Novo-Galiciana 17. Gymnosperms and Pteridophytes. Ann Arbor: Univ Mich Herbarium; 1992.

[101] MacNeish R. Ojo de Agua Cave -- Tmc 174. Report and catalog of excavated material. Andover MA: Peabody Mus Arch, Phillips Academy; 1954.

[102] MacNeish R. Romero’s cave. Report. Andover MA: Peabody Mus Arch, Phillips Academy; 1954.

[103] Kelley D. Valenzuela’s Cave (Tmc 248). Report. Andover MA: Peabody Mus Arch, Phillips Academy; 1954.

