# Supplementary material for: Ethnobotany of Mexican and northern Central American cycads (Zamiaceae)
Source: J Ethnobiol Ethnomed. 2019 Jan 18;15:4. doi: 10.1186/s13002-018-0282-z (PMC6339304; doi:10.1186/s13002-018-0282-z)
Supplement: Supplementary file 1 — Table S1. Ethnobotany of MNCA Cycads by Local Term. Contains tabulated qualitative ethnographic data arranged in columns by category. Entries are alphabetized by Local Term. Table S2. Ethnobotany of MNCA Cycads by Species. Distillation and reorganization of Supplementary Table 1 indicating presence (x) or absence of various ethnobotanical uses, organized by species (alphabetized by Latin name) (DOCX 161 kb) [file 13002_2018_282_MOESM1_ESM.docx]

**Table S1: Ethnobotany of MNCA cycads by local term**

| LOCAL TERM^a^ | TRANSLATION^b^ | LANGUAGE^c^ | ETHNIC GROUP/S^d^ | SPECIES | LOCATIONS^e^ | ALIMENTARY USES | ECONOMIC USES | AGRO-ECOLOGICAL USES | RELIGIOUS USES^f^ | OTHER USES | NOTES |
| --- | --- | --- | --- | --- | --- | --- | --- | --- | --- | --- | --- |
| -Not recorded- 104,MB08] | N/A | N/A | Mixtec | *Dioon planifolium* | Mexico-Zaragoza,Sta Cruz Itundijia (Oaxaca) | -Seeds boiled, then roasted on coals (formerly) |  | -Cultivated in home gardens, not for commercial trade |  | -As toys (seeds) |  |
| -Not recorded- [53] | N/A | N/A | Nahua, Tepehua, Totonaco | *Ceratozamia,* ‘*Dioon,*’ ‘*Zamia*’ spp. | Mexico-Sierra Norte region (Puebla) |  |  | -Believed to be parents of maize | -Altars, graves, and churches |  | No *Dioon* or *Zamia* known from the region |
| -Not recorded- [105,106] | N/A | N/A | Chinanteco | *Ceratozamia whitelockiana* | Mexico-Río Valle Nacional drainage basin; El Rincón—Sierra Norte region (Oaxaca) | -Yes (formerly?) |  | -Intentionally protected in traditional landscape management systems? |  | -Decorative (unspecified)  -Medicinal (unspecified)  -As toys (unspecified) |  |
| -Not recorded- [107] | N/A | N/A | Mestizo | *Ceratozamia mirandae* | Mexico-Frailesca region (Chiapas) | (formerly) |  |  | -Yes (unspecified) |  |  |
| -Not recorded- [108] | N/A | N/A | Totonac | *Ceratozamia totonacorum* | Mexico-Santiago Ecatlán, Sierra Norte region (Puebla) |  |  |  | -‘manage[d]…for ritual purposes’ |  |  |
| -Not recorded- [109] | N/A | N/A | Mestizo | *Ceratozamia kuesteriana* | Mexico (Tamaulipas) |  |  |  |  | -Decorative (unspecified)  -Medicinal (unspecified) |  |
| -Not recorded- [110] | N/A | N/A | Q’eqchi’ | *Zamia* sp. | Belize-southern |  |  |  |  | -Medicinal (unspecified) |  |
| -Not recorded- [111] | N/A | N/A | Mestizo | *Zamia loddigesii* | Mexico-Sierra de Santa Marta, Los Tuxtlas region (Veracruz) |  |  |  |  | -Medicinal: mashed stem starch applied externally to accelerate childbirth; stem burned and used to smoke the umbilical cord before cutting; used to cure skin lesions (‘*cancer’*) |  |
| -Recorded as ‘Oaxacan Dioons’- [112,MB08] | N/A | N/A | Indigenous | Mostly *Dioon merolae* | Mexico (Oaxaca) | -Consumed (*D. merolae*) | ‘leaves…are sold in the markets of Tehuantepec’ (for Christmas) | -Cultivated in church gardens for religious use | -Christmas  -Holy Week (*Semana Santa*) |  |  |
| -Unknown to informants- [MB16] | N/A | N/A | Cora, Mestizo | *Zamia paucijuga* | Mexico-Jalcocotán (Nayarit) | -Narcotic |  |  |  | -Medicinal: for headaches and muscle pains | See also ‘peyote’ |
| *[x]+yazn-goag* (*yaz[n]goo-a, yaz[n]gua*) [MB08] | ‘spiny palm tree,’ ‘spiny leaf’ (l.i.) | Zapotec | Zapotec | *Dioon holmgrenii* | Mexico-San Isidro, Santiago Textitlán (Oaxaca) | -Tortillas, from seeds (formerly) (always mixed with maize; 30 cm diameter)  -Detox. methods: lime, sometimes saltwater |  |  | -Holy Week (*Semana Santa*) |  | See also ‘jangó’ |
| *abuelo del maíz* [113] | ‘grandfather ofmaize’ | Spanish | [Not specified] | *Zamia loddigesii* | Mexico |  |  | -Associated with maize |  |  |  |
| *ahaatik [a] eem* [82] [MB16]{q.v. *konlif*} | ‘maize lord’ ([114] *ajātic*, Christ the Lord; *ēm*, maize) | Teenek | Teenek | *Ceratozamia latifolia* | Mexico-Huasteca region  (San Luis Potosí) |  |  |  |  |  |  |
| *aján* [MB16] {q.v. *konlif*} | ‘maize cob’ [114] *ajan*, maize cob) | Teenek | Teenek | *Ceratozamia latifolia* | Mexico-Aquismón (San Luis Potosí) |  |  |  |  |  |  |
| *alma* [52] {q.v. *tiusinte*} | ‘soul’)  (ref. germ) | Spanish | Mestizo | *Dioon mejiae* | Honduras |  |  |  |  |  |  |
| *alma de cintli* [MB16] {q.v. *teocintle* and *tzompoyo*} | ‘soul of maize ear’ | Spanish+  Nahua | Nahua | *Ceratozamia fuscoviridis,*  *Zamia fischeri, Zamia loddigesii* | Mexico-Huautla (Hidalgo) |  |  |  |  |  |  |
| *alma del maíz* [44] {q.v. *teocintle* and *tzompoyo*} | ‘soul of maize’ | Spanish | Nahua | *Ceratozamia fuscoviridis,*  *Zamia loddigesii* | Mexico-Huautla (Hidalgo) |  |  |  |  |  |  |
| *amendú* (*amenduay, amenduai*) [8,115,116,117] | N/A (*ay*, leaf’; *men*, stomach pain; *tuj*, rain [118]) | Zoque | Mestizo | *Ceratozamia alvarezii, Ceratozamia miqueliana, Ceratozamia norstogii, Ceratozamia robusta, Ceratozamia santillani, Ceratozamia vovidesii, Ceratozamia zoquorum* | Mexico-Selva Zoque region, including El Ocote Biosphere Reserve (Chiapas) | -Seeds (formerly?) |  |  | -Candlemas (2 Feb., *Día de la Candleria*), *C. robusta* [8]  -In *some* altars (leaves) for offerings [8] |  | Likely a Hispanicized Zoque term; potential Zoque roots suggested |
| *amigo de Dhipak* [MB16] {q.v. *konlif*} | ‘friend of *Dhipak’* | Spanish+  Teenek | Teenek | *Ceratozamia latifolia* | Mexico-Aquismón (San Luis Potosí) |  |  |  |  |  |  |
| *amigo del maíz* [22] | ‘friend of maize’ | Spanish | Mestizo, Totonac, (Chontal?) | *Zamia loddigesii, Zamia vaz-quezii* | Mexico-Papantla (Veracruz) (Tabasco) |  |  | -Associated with maize |  | -Medicinal: Used to disinfect umbilical cord at childbirth (Tabasco) |  |
| *apaguaste* [52] {q.v. *tiusinte*} | (ref. toxic water in which raw seeds have been steeped) | Spanish | Mestizo | *Dioon mejiae* | Honduras |  |  |  |  |  |  |
| *ayudante de Dhipak* [MB16] {q.v. *konlif*} | ‘*Dhipak’s* helper’ | Spanish+  Teenek | Teenek | *Ceratozamia latifolia* | Mexico-Aquismón (San Luis Potosí) |  |  |  |  |  |  |
| *bijio’a* [MB09] | N/A | Xi’iuy | Xi’iuy | *Ceratozamia microstrobila* | Mexico-Tamasopo (San Luis Potosí) |  |  |  |  |  |  |
| *bo’jor* [MB16] {q.v. *konlif*} | ‘maize cob of *konlif’* (l.i.)  (ref. male cone) (*bojol*, ‘elote’) | Teenek | Teenek | *Ceratozamia latifolia* | Mexico-Aquismón (San Luis Potosí) |  |  |  |  |  |  |
| *bola* [MB16] (*bolita*) | ‘ball’  (ref. fem. cone) | Spanish | Mestizo | various species | Honduras |  |  |  |  |  |  |
| *bola pachona* [72] {q.v. *chamal*} | ‘fluffy ball’ (ref. male cone) | Spanish | Mestizo, Xi’iuy | *Dioon edule* | Mexico-Pamería region  (San Luis Potosí) |  |  |  |  |  |  |
| *brea* [8] {q.v. *tiusinte*} | ‘tar’ (ref. fem. cone mucilage) | Spanish | Mestizo | *Dioon mejiae* | Honduras |  |  |  |  |  |  |
| *cabeza* [MB16] | ‘head’ (ref. fem. cone) | Spanish | Mestizo | various species | widespread |  |  |  |  |  |  |
| *cahua* [111] {q.v. *chak waj* for unrelated term} {q.v *maíz de coxca*} | N/A | Zoque | Zoque | *Zamia*  *loddigesii* | Mexico (S. Veracruz) |  |  |  |  |  | Source records as of ‘Zoque-Popoluca’ origin; may not be reliable |
| *camote* [45,MB16] | ‘sweet potato’  (ref. fem. cone) | Spanish | Mestizo | various species | Mexico-widespread |  |  |  |  |  |  |
| *camotillo* [51,119,120,121,122,123, J Vannini, pers. comm.] | ‘little sweet potato’ | Spanish | Mestizo,  Indigenous (Chorti, Miskito, Pech, others?) | *Ceratozamia hondurensis*, *Zamia herrerae, Z. monticola, Z. onanreyesii, Z. oreillyi, Z. prasina, Z. sandovalii, Z. splendens, Z. standleyi, Z. tuerckheimii, Z. variegata* | Belize, Guatemala, Honduras-widespread, Mexico (Chiapas) | -as atole (slurry) for children (detox.: seeds soaked 8 days in water) (Trujillo, Honduras) |  | -As rodenticide  -Planted in gardens (Cobán, Alta Verapaz, Guatemala) [124]-*Z. tuerckheimii* |  | -As means of assassination (stem sun-dried, ground, administered in drink)  -Medicinal: dry ‘roots’ (stem) chewed, saliva ingested, provides cough relief [122]  -Medicinal: ‘roots’ (stem) chewed, saliva ingested, improve singing voice [122]  -Red dye for tobacco pipes and other wood (from ‘roots,’ stem, with lime) [122] | Possibly also in Oaxaca and on Gulf of Mexico coast, thus including *Z. loddigesii* and *Z. spartea*, as per some references |
| *carrete* [125] | ‘ox cart’ (ref. male cone) | Spanish | Mixe | *Ceratozamia mixeorum* | Mexico-Sierra Mixe region  (Oaxaca) |  |  |  |  | -As toys (male cone) |  |
| *cáscara* (*cascarita*) | ‘shell’ (ref. sclerotesta and/or sarcotesta) | Spanish | Mestizo | various | widespread |  |  |  |  |  |  |
| *chak waj* (*cahua, chac’hua, chac hua, chacal jua, chacuhua, chakua, chak wa, palmita, palmito*) [53,119,126,127,128] | ‘red tortilla’ (*chak*, red; *waaj*, maize tortilla, bread, tamale). Refers to a traditional food from maize, *Ipomoea*, etc. | Yucatec | Yucatec | *Zamia prasina* | Mexico (Campeche, Quintana Roo, Yucatan) | -Bread from stems and roots |  | -As rodenticide (Xocen, Yucatán [53])  -Cultivated in kitchen gardens [127] | -Used by shamans |  | Often as ‘*Z. loddigesii*’ |
| *chamal* [63,72,129,MB16] {q.v. *damew* for Xi’iuy uses reported by Chemin-Bassler} | ‘plant of *tierra fria*’ (ref. populations at higher altitude in the Sierra above the Huasteca lowlands) {q.v. *tzamaal*} | Nahua, fr. Teenek | Mestizo,  Nahua, Xi’iuy | *Ceratozamia fuscoviridis* [45] and other *Ceratozamia* spp*.*, *Dioon angustifolium, Dioon edule,Dioon purpusii, Dioon spinulosum* [53]*, Dioon tomasellii,* mostly in northeastern Mexico | Mexico (Guanajuato,  Hidalgo, Jalisco [formerly], Nuevo Leon, Oaxaca [*D. purpusii*, Tomellín Canyon [130]], Queretaro, San Luis Potosí, Tamaulipas, Veracruz, Yucatan [in gardens, Izamal, ref. Standley (1930) in [53]]) | (Currently, San Luis Potosí and Queretaro only, unless indicated otherwise. Formerly in Tamaulipas (though [109] suggests currently, roasted seeds], Hidalgo, and elsewhere)  -Atoles from roasted and ground sarcotestas [MB09]  -Atoles from seeds  -Detox. methods: ash, lime, ash + lime)  -Entire female cone, chopped into small pieces, in water with sugar: Los Pocitos, Santa Catarina, SLP [72](formerly)  -Gorditas from seeds and from sarcotestas  -Narcotic (root) [MB16]  -Sarcotesta eaten raw, in preserves, and roasted [63]  -Tamales from seeds  -Tamales from stems (formerly)  -Tortillas from sarcotestas [63] (Nuevo León) (sometimes mixed with maize or wheat flour); Pamería region, SLP (‘sweet or salty’) [72]  -Tortillas from seeds | -Sold by harvesters directly to users  -Sold in local markets (seeds, tamales from seeds)  -Presence in long-distance exchange networks  -Bartered for other products | -Associated with maize  -Co-managed with cattle  -Dioecy recognized  -Cultivated in home gardens  -Existence of planted populations  -Fed to domestic animals (sarcotestas: [63])  -Intentionally protected in traditional landscape management systems  -Intentionally managed for in traditional landscapes | -Used by shamans  -Candlemas (2 Feb.), *D. edule*, Pisaflores [46]  -Christmas Eve-nativity scenes: Tlahuiltepa, Hidalgo [45]  -Day of the Dead (Nov. 2); food also included in offerings at altars  -Holy Week (*Semana Santa*)  -St. James the Apostle Day (Jul. 25): Tepehuacán de Guerrero, Hidalgo [45]  -St. John’s Day (24 Jun.): *D. edule* in Pacula, Hidalgo [46]  -St. Sebastian’s day (Jan. 20): Tlahuiltepa, Hidalgo [45]  -St. Nicholas’s Day (Sept. 10): Xicoatlán, Hidalgo [45]  -Virgin of Guadalupe Day (Dec. 12): Xicoatlán, Hidalgo [45]; Pamería region, SLP [72]  -Funerals: Pamería region, SLP [72] | -Connected explicitly to sense of place  -Corner posts for houses [MB16] (Sierra Gorda region, Qro)  -Decorative: Numerous non-religious uses for leaves during public ceremonies such as Independence Day (Sept. 26), graduations, weddings  -Medicinal: Seeds, to cure neuralgia (Nuevo León) [129]  -Thatch for roofs (Tlahuiltepa, Hidalgo [45]) |  |
| *chamalar* {q.v. *chamal*} [MB09] | ‘*chamal* grove’ | Nahua+  Spanish | Mestizo | *Dioon edule* | Mexico-Tanchachín (San Luis Potosí) |  |  |  |  |  |  |
| *chamalero* {q.v. *chamal*} [72] | ‘*chamal* gatherer’ | Nahua+  Spanish | Mestizo | *Dioon angustifolium,*  *Dioon edule* | Mexico (San Luis Potosí, Tamaulipas) |  |  |  |  |  |  |
| *chamalillo* (*chamal chico*, *palmilla chamalillo* {q.v. *chamal*} [45,46,54,113] | ‘little *chamal’* | Nahua+  Spanish | Mestizo, Nahua, Teenek | various *Zamia* and *Ceratozamia* in northeastern Mexico, including *C. chamberlainii* [131], *C. fuscoviridis* [45], *C. hildae* [54], *Z. fischeri* [54]*, C. fischeri* [113] | Mexico (La Misión, Hidalgo [45], San Luis Potosí, Queretaro, Tamaulipas). Zimapán, Hidalgo: *C. sabatoi* as ‘chamal chico’ [46] | -Tortillas and atoles from C. *microstrobila* seeds; can be mixed with *D. angustifolium* seeds  -Detox. method: seeds sun-dried, boiled; |  |  |  |  |  |
| *chicalito* (*palma [de] chicalite*) [31,111,132] | ‘little *chical’* (Nahua term in Chinantec usage for dried, hollowed Cucurbit gourds used to carry water [133]), (ref. sarcotesta) ([111]=incorrect, ‘voz zapoteca’) | Nahua+Spanish | Chinantec, Mazatec?, Mestizo? | *Dioon spinulosum* | Mexico-Papaloapan valley  (Oaxaca, Veracruz) |  | -Seeds and leaves sold in markets | -Cultivated in gardens as ornamentals | -Cultivated in church gardens | -As toys (sclerotesta used to make ‘zumbadores,’ bullroarers) |  |
| *chicomesintli* [44] {q.v. *teocintle* and *tzompoyo*} | ‘seven-ear’ | Nahua | Nahua | *Ceratozamia fuscoviridis,*  *Zamia loddigesii* | Mexico-Huautla (Hidalgo) |  |  |  |  |  |  |
| *chicomexochi[t]l* [MB16] {q.v. *teocintle* and *tzompoyo*} | ‘seven-flower’ | Nahua | Nahua | *Ceratozamia fuscoviridis,*  *Zamia loddigesii* | Mexico- Huautla (Hidalgo) |  |  |  |  |  |  |
| *chukmal* [MB16] {q.v. *konlif*} | ‘small hairs on the cone’ (l.i). (ref. tomentum on female cone) | Teenek | Teenek | *Ceratozamia latifolia* | Mexico-Aquismón (San Luis Potosí) |  |  |  |  |  |  |
| *chum* [MB16] {q.v. *konlif*, *tzamaal*} | ‘point’ (ref. tip of fem. cone) ([114] *chūm*) | Teenek | Teenek | *Ceratozamia latifolia,*  *Dioon edule* | Mexico-Aquismón (San Luis Potosí) |  |  |  |  |  |  |
| *churute* (*churutón*) [52] {*q.v. tiusinte*} | ‘male cone’ | Spanish | Mestizo | *Dioon mejiae* | Honduras |  |  |  |  |  |  |
| *cintli* [45] {q.v. *teocintle* and *tzompoyo*} | ‘maize ear’ | Nahua | Nahua | *Zamia loddigesii* | Mexico-Tlazongo and Zapotitla, Huazalingo (Hidalgo) |  |  |  |  |  |  |
| *cintli cuautitla* [MB16] {q.v. *teocintle* and *tzompoyo*} | ‘forest maize ear’ | Nahua | Nahua | *Ceratozamia*  *fuscoviridis,*  *Zamia loddigesii* | Mexico-Huautla (Hidalgo) |  |  |  |  |  |  |
| *cintli i nana* [45] {q.v. *teocintle* and *tzompoyo*} | ‘mother of maize ear’ | Nahua | Nahua | *Zamia loddigesii* | Mexico-Huautla (Hidalgo) |  |  |  |  |  |  |
| *coatl* [44] | N/A (ref. medicine) | Nahua | Teenek | *Zamia fischeri* | Mexico-Aquismón (San Luis Potosí) |  |  |  |  | -Medicinal: for ‘malos aires’ |  |
| *cocalito* [119] | ‘little coconut palm’ | Spanish | Mestizo | *Zamia prasina* | Guatemala |  |  |  |  |  |  |
| *comida antígua* [MB08] | ‘old-time food’ | Spanish | Chontal de Oaxaca | *Dioon merolae* | Mexico-San Lucas, Yautepec (Oaxaca) |  |  | Associated with maize  -Pollination mechanisms recognized (pollen said to be carcinogenic) |  | -Belief in carcinogenic properties |  |
| *comida de gentíl* (*comida de los abuelos*) [MB08] | ‘old-timers’ food ,’ ‘grandparents’ food’ | Spanish | Chontal de Oaxaca | *Dioon merolae* | Mexico-Santa Maria Ecatepec; San Pedro Sosoltepec; San Lucas (Oaxaca) | -Sarcotestas: boiled; in sweetened preserves; raw |  | -Associated with maize |  | -Public signs on walls (leaves) for important secular events |  |
| *compañero del maíz* [MB16] (*compañero de la mazorca*){q.v. *konlif*} | ‘maize’s companion’ | Spanish+  Teenek | Teenek | *Ceratozamia*  *latifolia* | Mexico-Aquismón (San Luis Potosí) |  |  |  |  |  |  |
| *corn palm* [134] |  | English | Mopan Maya | *Zamia decumbens* | Belize-Maya Mts. region |  |  | -Associated with maize (name) |  |  |  |
| *corona de tiusinte* [52] {q.v*. tiusinte*} | ‘*tiusinte* leaf wreath’ | Spanish | Mestizo | *Dioon mejiae* | Honduras |  |  |  |  |  |  |
| *costilla de león* [31,119,124] | ‘puma’s rib’ (ref. leaf) | Spanish | Mestizo | *Ceratozamia robusta, C. ‘mexicana’, C. tenuis* | Guatemala; Mexico  (Veracruz) |  |  | -As pesticide  -Planted in gardens (Veracruz) (Huahuetenango, Guatemala) [81] |  |  |  |
| *coyolillo* [22] | ‘little coyol’ (ref. *Acrocomia mexicana* palm) | Spanish | Mazatec, Mestizo | *Dioon spinulosum* | Mexico (Oaxaca,  Veracruz) |  |  |  |  |  |  |
| *coyolito de cerro* [31] | ‘little hill coyol’ | Spanish | Mazatec, Mestizo | *Dioon spinulosum* | Mexico-Tuxtepec (Oaxaca) |  |  |  |  |  |  |
| *cuacintli* (*cuacintle*) [45] {q.v. *teocintle* and *tzompoyo*} | ‘forest maize ear’ | Nahua | Mestizo | *Ceratozamia*  *fuscoviridis* | Mexico-Quetzaltzongo, Tlanchinol (Hidalgo) |  |  |  |  | -Thatch from leaves (formerly) |  |
| *cumbito* [49] (*cumbillo*, *cumbo*) {q.v. *tiusinte*} | ‘little container’ (ref. sarcotesta) | Spanish | Mestizo | *Dioon mejiae* | Honduras |  |  |  |  |  |  |
| *cunelcintli* [MB16] {q.v. *teocintle* and *tzompoyo*} | ‘maize ear children’ (ref. seedlings) | Nahua | Nahua | *Zamia loddigesii* | Mexico-Huautla  (Hidalgo) |  |  |  |  |  |  |
| *dameu* (*dameaō, dameaw, nameu*) [135] | N/A | Xi’iuy | Xi’iuy | *Dioon edule* | Mexico-Sta Ma. Acapulco, Sta Catarina; La Palma, Tamasopo (San Luis Potosí) (Queretaro) | -Atoles  -Breads (with maize)  -Tamales and tortillas from seeds (sometimes mixed with palm heart pieces and maguey leaves [73,86] ) (tamales often filled with meat and other ingredients)  -Tamales and tortillas from sarcotestas (with maguey leaves and palm hearts) | -Sold by harvesters directly to users  -Sold in local markets  -Presence in long-distance exchange networks  -Bartered for other products | -Associated with maize  -Dioecy recognized  -Fed to domestic animals  -Intentionally protected within traditional landscape management systems | -Day of the Dead  -Holy Week  -Patron saints' days | -Connected explicitly to sense of place |  |
| *dhókob* [MB16] {*q.v. konlif*} | ‘leaf of *konlif’* | Teenek | Teenek | *Ceratozamia latifolia* | Mexico-Aquismón  (San Luis Potosí) |  |  |  |  |  |  |
| *ejatal em* [44] {q.v. *konlif*} | ‘the life of maize’ ([114] *ejattalāb*, soul, life) | Teenek | Teenek | *Ceratozamia*  *latifolia* | Mexico-Aquismón (San Luis Potosí) |  |  |  |  |  |  |
| *ekmito’tol* [MB16] {*q.v. konlif*} | ‘outer shell’ (ref. female cone scales) | Teenek | Teenek | *Ceratozamia*  *latifolia* | Mexico-Aquismón (San Luis Potosí) |  |  |  |  |  |  |
| *el rey [y carne] de las mazorcas* [MB16] {q.v. *konlif*} | ‘king [and flesh] of maize ears’ | Spanish | Teenek | *Ceratozamia*  *latifolia* | Mexico-Aquismón (San Luis Potosí) |  |  |  |  |  |  |
| *elote [de monte]* [44]{q.v. *teocintle* and *tzompoyo*} | ‘forest maize ear’ | Spanish | Nahua | *Ceratozamia*  *fuscoviridis,*  *Zamia loddigesii* | Mexico-Huautla (Hidalgo) |  |  |  |  |  |  |
| *elotillo* [44] {q.v. *teocintle* and *tzompoyo*} | ‘tiny maize ear’ (ref. male cone) | Nahua + Spanish | Nahua | *Ceratozamia*  *fuscoviridis,*  *Zamia loddigesii* | Mexico-Huautla (Hidalgo) |  |  |  |  |  |  |
| *elotito de konlib* [44] (*elote de konlib*){q.v. *konlif*} | ‘*konlib’s* tiny maize ear’ (ref. male cone) | Teenek+ Nahua+ Spanish | Teenek | *Ceratozamia*  *latifolia* | Mexico-Aquismón (San Luis Potosí) |  |  |  |  |  |  |
| *elotsintli* (*elolcintli*) [44]{q.v. *teocintle* and *tzompoyo*} | ‘maize ear’ | Nahua | Nahua | *Zamia loddigesii* | Mexico-Huautla (Hidalgo) |  |  |  |  |  |  |
| *eloyo* [MB16] /{q.v. *teocintle* and *tzompoyo*} | N/A (ref. to whole plant) | Nahua | Nahua | *Zamia loddigesii* | Mexico-Huautla (Hidalgo) |  |  |  |  |  |  |
| *enchamalamiento* (*enchamalado*) {q.v. *chamal*} [72] | ‘*chamal* poisoning’ (ref. nerve damage to livestock) | Spanish+  Nahua | Mestizo | *Dioon angustifolium,*  *Dioon edule* | Mexico-northeast |  |  |  |  |  |  |
| *enchamalar* [72] {q.v. *chamal*} | ‘to become poisoned by eating *chamal* leaves’ [livestock] | Spanish+  Nahua | Mestizo | *Dioon edule* | Mexico-Pamería region (San Luis Potosí) |  |  |  |  |  |  |
| *espadaña* (*flor de espadaña*) [8,80,136,MB03] | pertaining to ‘sword’ (ref. leaf) | Spanish | Chiapanec,  Mestizo, Zoque | *Ceratozamia vovidesii ,*  *Dioon merolae* | Mexico-Cintalapa, Jiquipilas, Suchiapa, Terán, Villaflores [8] (Chiapas) |  |  | -Intentionally protected within traditional landscape management systems  -Cultivated in home gardens | -Dia de la Santa Cruz (3 May)  -Involved in pilgrimages (‘Topada de la Flor de Espadaña’) (26 Apr-3 May)  -Patron saints’ days | -Connected explicitly to sense of place  -Decorative (Cintalapa) for various celebrations, including weddings |  |
| *espadañero* [8,80] (*hojero*) {q.v. *espadaña*} | ‘cycad gatherer’ | Spanish | Chiapanec,  Mestizo | *Dioon merolae* | Mexico-Jiquipilas, Suchiapa  (Chiapas) |  |  |  |  |  |  |
| *espíritu* [52] {q.v. *tiusinte*} | ‘spirit’ (ref. germ) | Spanish | Mestizo | *Dioon mejiae* | Honduras |  |  |  |  |  |  |
| *espíritu del monte* [MB16] {q.v. *konlif*} | ‘spirit of the forest’ | Spanish | Teenek | *Ceratozamia latifolia* | Mexico-Aquismón (San Luis Potosí) |  |  |  |  |  |  |
| *flor* | ‘flower’ (ref. male cone) | Spanish | widespread | *various* | widespread |  |  |  |  |  |  |
| *flor de Dhipak* [MB16] {q.v. *konlif*} | ‘*Dhipak’s* flower’ (ref. male cone) | Spanish+  Teenek | Teenek | *Ceratozamia latifolia* | Mexico-Aquismón  (San Luis Potosí) |  |  |  |  |  |  |
| *flor de konlif* [MB16] {q.v. *konlif*} | ‘*konlif’s* flower’ (ref. male cone) | Spanish+ Teenek | Teenek | *Ceratozamia latifolia* | Mexico-Aquismón  (San Luis Potosí) |  |  |  |  |  |  |
| *grano vano* [52] {*q.v. tiusinte*} | ‘useless grain’ (undeveloped seed) | Spanish | Nahoa de Honduras | *Dioon mejiae* | Honduras-Guata |  |  |  |  |  |  |
| *gusanito* [MB09] {q.v. *chamal*} | ‘little worm’ (ref. germ) | Spanish | Xi’iuy | *Dioon edule* | Mexico-Santa Catarina (San Luis Potosí) |  |  |  |  |  |  |
| *gusano de konlif* [MB16]{q.v. *konlif*} | ‘*konlif’s* caterpillar’ (ref. *Eumaeus sp.*) | Spanish+ Teenek | Teenek | *Ceratozamia*  *latifolia* | Mexico-Aquismón (San Luis Potosí) |  |  |  |  |  |  |
| *gusi* (*gusi+*) [135] {q.v. *dameu*} | ‘seeds’ | Xi’iuy | Xi’iuy | *Dioon edule* | Mexico-Santa Ma. Acapulco  (San Luis Potosí) |  |  |  |  |  |  |
| *ha-hé-uma* [MB08] | ‘tortilla’ or ‘la gordita’ (l.i.) (*hé*, tortilla [133]) | Chinantec | Chinantec | *Dioon spinulosum* | Mexico-Cerro Tepezcuintle, Tuxtepec (Oaxaca) | -Tortillas from seeds  -Gorditas  -Detox. method: boiled and ground | -Tortillas from seeds sold in local markets |  |  |  |  |
| *helecho* [MB16] {q.v. *palmito de cerro*) | ‘fern’ | Spanish | Mestizo | *Zamia paucijuga* | Mexico-El Teocinte, Tomatlán (Jalisco) |  |  |  |  |  |  |
| *helecho marino* [137] | ‘sea[side] fern’ | Spanish | Mestizo | *Zamia furfuracea* | Mexico (Veracruz) |  |  |  |  |  |  |
| *hermano de Dhipak* [44]{q.v. *konlif*} | ‘Thipaak’s brother’ | Spanish+  Teenek | Teenek | *Ceratozamia*  *latifolia* | Mexico-Aquismón (San Luis Potosí) |  |  |  |  |  |  |
| *hma-bu-ma* (*ma-bu-ma*) [MB08] | (*hma*, tree [133]) | Chinantec | Chinantec | *Dioon spinulosum* | Mexico-Cerro Tepezcuinte, Tuxtepec (Oaxaca) | -Sarcotesta (in Mazin Grande)  -Stem (female) |  |  | -All Souls/All Saints days (1-2 Nov, Todos Santos/Día de los Muertos), San Juan Bautista Valle Nacional (Oaxaca) | -Necklaces and toys (from sclerotestas) [9] (ref. Miguel Martínez Alfaro pers. com.) | Some sources incorrectly ref. as *‘Zamia loddigesii’* |
| *hma-bu-ma-moh (ma-bu-ma-mo)* [MB08] | (*ma-moh,* palm, l.i.) (*hma*, tree; *moh*, leaf, [133]) | Chinantec | Chinantec | *Zamia loddigesii* | Mexico-Cerro Tepezcuinte, Tuxtepec (Oaxaca) |  |  |  |  | --Ripe seeds used as coloring agent in schools |  |
| *hojero* [80] {q.v. *espadaña*) | ‘leaf collector’ (ref. pilgrims in Día de la Cruz pilgrimage) | Spanish | Chiapanec | *Dioon merolae* | Mexico-Terán (Chiapas) |  |  |  |  |  |  |
| *hueso de pescado* [22] | ‘fishbone’ (ref. leaf) | Spanish | Indigenous | *Dioon sp.* | Mexico (Oaxaca) |  |  |  |  |  |  |
| *ich lab* [MB16] {q.v. *tzamaal*} | ‘seed’ (ref. entire seed, including outer coverings) | Teenek | Teenek | *Dioon edule* | Mexico-Aquismón (San Luis Potosí) |  |  |  |  |  |  |
| *ichich* [MB16] {q.v. *tzamaal*} | ‘heart’ (‘corazón,’ ref. seed starch) ([114] *ichīch*, heart, seed) | Teenek | Teenek | *Dioon edule* | Mexico-Aquismón (San Luis Potosí) |  |  |  |  |  |  |
| *Iljye’e dameaw* (*LJE’E namew’*) [73] {q.v. *dameu*} | ‘*chamal* tamales’ | Xi’iuy | Xi’iuy | *Dioon edule* | Mexico-San Pedro, Sta Ma Acapulco, Sta Catarina  (San Luis Potosí, Queretaro) |  |  |  |  |  |  |
| *Ilye’e dameaw kon garbants* [73] {q.v. *dameu*} | ‘chamal tamales with garbanzos’ | Xi’iuy | Xi’iuy | *Dioon edule* | Mexico-Huizachal, La Palma, Tamasopo; La Barranca, Sta Ma Acapulco, Sta Catarina (San Luis Potosí) |  |  |  |  |  |  |
| *inik konlif* [MB16] {q.v. *konlif*} | ‘male *konlif’* | Teenek | Teenek | *Ceratozamia*  *latifolia* | Mexico-Aquismón (San Luis Potosí) |  |  |  |  |  |  |
| *inik tzamaal* [MB16] {q.v. tzamaal} | ‘male *tzamaal*’ | Teenek | Teenek | *Ceratozamia*  *latifolia* | Mexico-Aquismón (San Luis Potosí) |  |  |  |  |  |  |
| *itajka* [45] {q.v. *teocintle*} | ‘fruit’ (generic ref.) | Nahua | Nahua | cycad spp. | Mexico (Hidalgo) |  |  |  |  |  |  |
| *jangó* (*palma de jangó, palma de xangó*) [115] | N/A {q.v. *[x]+yazn-goag*} | Zapotec? | Unspecified | *Ceratozamia norstogii* | Mexico-San Fernando  (Chiapas) | -Seeds |  |  |  |  | Other *Ceratozamia* spp. may be involved |
| *jilote* {q.v. *chamal*, *tiusinte*, *tzompoyo* and *teocintle*} [72] | ‘green maize ear’ (ref. female cone) | Spanish | Mestizo | various | Honduras [8], Mexico-widespread [MB16a] |  |  |  |  |  |  |
| *jilotear* {q.v. *chamal*, *tiusinte*, *tzompoyo* and *teocintle*} | ‘to sprout a green maize ear’ (ref. female cone) | Spanish | Mestizo | various | Mexico-Aquismón (San Luis Potosí) [MB16] |  |  |  |  |  |  |
| *jiñote* [49] {q.v *tiusinte*} | ‘bad tamale’ | Spanish | Mestizo | *Dioon mejiae* | Honduras |  |  |  |  |  |  |
| *jojo* (*jobal, jojobal*) [MB16] {q.v. *tzamaal*} | N/A (ref. ‘cotton’= tomentum on female cone; tomentum-covered cone scale) ([114] *jojob*, maize husk) | Teenek | Teenek | *Ceratozamia latifolia , Dioon edule* | Mexico-Aquismón (San Luis Potosí) |  |  |  |  |  |  |
| *junimbola* (*jun ibola, juninbola*) [38,MB16] {q.v. konlif} | ‘base of *mono’* (l.i.){q.v.} | Teenek | Teenek | *Ceratozamia latifolia* | Mexico-Aquismón (San Luis Potosí) |  |  |  |  |  |  |
| *k’id* [MB16] {q.v. *konlif*} | ‘spine’ (on leaf) ([114] *q’uīth*, spine) | Teenek | Teenek | *Ceratozamia latifolia* | Mexico-Aquismón  (San Luis Potosí) |  |  |  |  |  |  |
| *kanaw namew* [86] (*kanau nameu*) {q.v. *dameu*} | ‘*chamal* head’ (ref. fem. cone) | Xi’iuy | Xi’iuy | *Dioon edule* | Mexico-Santa Ma. Acapulco  (San Luis Potosí), (Queretaro) |  |  |  |  |  |  |
| *konlif* (*condif, conlif, kombi, kombil, konbi, kondif, konfi, konlib*) [38,MB16] | ‘plant of sand’ [82] | Teenek | Teenek | *Ceratozamia*  *latifolia* (and other local *Ceratozamia*, including *C. hildae* and *C. microstrobila*) | Mexico-Huasteca region  (San Luis Potosí) | -Atole (formerly)  -‘Bolitas’=spherical tamales  -Sarcotestas, raw  -Tortillas from seeds  -Detox. methods:ash; lime; ash+lime; boiled) | -Leaves sold by harvesters directly to users | -Associated with maize  -Dioecy recognized  -Intentionally protected within traditional landscape management systems  -Pollination mechanisms recognized | -Undivulged shamanic uses  -Bishop’s visits (mostly formerly)  -Patron saints’ and patron Virgin Mary days (mostly formerly)  -Offerings in altars for maize ceremonies | -Medicinal: to cure ‘llagas’ (injuries) on the feet  -Medicinal: poultice of mashed starch directly on wound  -Combs (male cone) formerly)  -Connected explicitly to sense of place  -Influence on weather (brings rain, ‘pulls down’ moisture from the clouds)  -As toys (seeds) |  |
| *konlif dhum’* (*dhumalan konlif, konlif-dhum*) [MB16] {q.v. *konlif*} | ‘*konlif’s* caterpillar’ (ref. *Eumaeus* sp.) ([114] *thūm*, gusano) | Teenek | Teenek | *Ceratozamia latifolia* | Mexico-Aquismón (San Luis Potosí) |  |  |  |  |  |  |
| *kun* [TP16] | N/A | Totonac | Totonac | *Ceratozamia totonacorum* | Mexico-Sierra Norte region  (Puebla) |  |  |  |  |  |  |
| *kuojxiuit* [TP17] | ‘trunk with many leaves’ | Nahua | Nahua | *Ceratozamia totonacorum* | Mexico-Cuetzalán (Puebla) |  |  |  | -Yes (unspecified) |  |  |
| *la carne del maíz* [MB16] {q.v. *konlif*} | ‘the body of maize’ | Spanish | Teenek | *Ceratozamia latifolia* | Mexico-Aquismón (San Luis Potosí) |  |  |  |  |  |  |
| *la-fané-tejuá* [115] | ‘ancient food’ [138] | Chontal de Oaxaca | Chontal de Oaxaca | *Dioon merolae* | Mexico (Oaxaca) |  |  | -Associated with maize |  |  |  |
| *lan-zi-lé (lan-zi-li)* [115] | N/A | Chontal de Oaxaca | Chontal de Oaxaca | *Dioon merolae* | Mexico (Oaxaca) |  |  | -Associated with maize |  |  |  |
| *madre del maíz* [MB16] {q.v. *konlif*} | ‘mother of maize’ | Spanish | Teenek | *Ceratozamia latifolia* | Mexico-Aquismón (San Luis Potosí) |  |  |  |  |  |  |
| *maíz de coxca* [111] | ‘*coxca* maize’ (*coxca*, transl. unknown) ([118] *cotzac*, hill) | Spanish+  Zoque | Zoque | *Zamia loddigesii* | Mexico (S. Veracruz) | -Seeds (roasted) |  | -Associated with maize (name) |  |  | Source records as of ‘Zoque-Popoluca’ origin; may not be reliable |
| *maíz de Dios* {q.v. *tzompoyo* and *teocintle*}[MB15] | ‘God’s maize’ | Spanish | Mestizo, Nahua | *Ceratozamia fuscoviridis* | Mexico-Tlanchinol (Hidalgo) |  |  |  |  |  |  |
| *maíz del cerro* [44] | ‘hill maize’ | Spanish | Nahua | *Ceratozamia fuscoviridis* | Mexico-Huasteca region (Hidalgo) |  |  | -Associated with maize |  |  |  |
| *maíz del monte* [MB09] {q.v. *chamal*} | ‘forest maize’ | Spanish | Mestizo, Xi’iuy | *Dioon edule* | Mexico-Pamería (San Luis Potosí) |  |  |  |  |  |  |
| *maíz gordo* [MB09] {q.v. *chamal*} | ‘fat maize’ | Spanish | Mestizo, Xi’iuy | *Dioon edule* | Mexico-Pamería (San Luis Potosí) |  |  |  |  |  |  |
| *maíz viejo* (*palo maíz viejo*) [112,139,MB17,MA Pérez Farrera, pers. comm.] | ‘old maize’ | Spanish | Chontal de Oaxaca | *Dioon merolae* | Mexico-Huamelula (Oaxaca) | -Tortillas from sarcotestas (formerly) |  | -Associated with maize  -Fed to domestic animals |  |  |  |
| *mamá de las mazorcas* [MB16]{q.v. *konlif*} | ‘mother of the maize ears’ | Spanish | Teenek | *Ceratozamia latifolia* | Mexico-Aquismón (San Luis Potosí) |  |  |  |  |  |  |
| *mamá del elote* (*madre del elote*) [MB16] {q.v. *tzompoyo* and *teocintle*} | ‘mother of maize ear’ | Spanish | Nahua | *Ceratozamia fuscoviridis,*  *Zamia fischeri, Zamia loddigesii* | Mexico-Huautla (Hidalgo); Aquismón (San Luis Potosí) |  |  |  |  |  |  |
| *mamá del maíz* [MB16] {q.v. *tzompoyo* and *teocintle*} | ‘mother of maize’ | Spanish | Nahua | *Ceratozamia fuscoviridis,*  *Zamia fischeri,Zamia loddigesii* | Mexico-Huautla (Hidalgo) |  |  |  |  |  |  |
| *manu* [44] {q.v. *konlif*} | ‘strength/  power of maize’ l.i. | Teenek | Teenek | *Ceratozamia latifolia* | Mexico-Aquismón  (San Luis Potosí) |  |  |  |  |  |  |
| *mata ratón* [113,120] | ‘rat killer’ | Spanish | Indigenous, Mestizo | *Zamia loddigesii* [113]*, Zamia prasina* [120] | Belize, Mexico |  |  | -As rodenticide (presumed, from name) |  |  |  |
| *mazacopa* [108] | ‘ball head’ (ref. fem. cone) | Zoque | Zoque | *Ceratozamia chimalapensis* | Mexico-Chimalapas  (Chiapas) | -Sarcotestas  -Flour from seeds |  | -As rodenticide (ground seed)  -As insecticide (ground seed with honey) |  |  |  |
| *mazorca* (*mazorquita*) {[MB17] q.v. *palma sol*} | ‘maize ear’ | Spanish | widespread | *various spp.* | Honduras, Mexico |  |  |  |  |  |  |
| *miim* [MB16] (*mim*) {q.v. *konlif*} | ‘grandmother’ ([114] *mīm*, señora) | Teenek | Teenek | *Ceratozamia latifolia* | Mexico-Aquismón (San Luis Potosí) |  |  |  |  |  |  |
| *mim konlif* [MB16] {q.v. *konlif*} | ‘grandmother of *konlif’* | Teenek | Teenek | *Ceratozamia latifolia* | Mexico-Tancuime, Aquismón (San Luis Potosí) |  |  |  |  |  |  |
| *mono* [MB16] {q.v. *konlif*} | ‘ball-like’ (ref. subterranean stem) | Teenek | Teenek | *Ceratozamia latifolia* | Mexico-Tanute, Aquismón (San Luis Potosí) |  |  |  |  | -Medicinal: for *Tinea pedis* infections |  |
| *morrito* [139] | ‘little morro’ (ref. *Crescentia cujete*?) | Spanish | Mestizo | *Dioon merolae* | Mexico (Oaxaca) |  |  |  |  |  |  |
| *nèh-k'ambur* [140] | ‘tail of Great Curassow’ (ref. *Crax rubra*) | Lacandón | Lacandón | *Zamia* sp. | Mexico-Selva Lacandona region (Chiapas) |  |  |  |  |  | Source lists ‘*Dioon edule*’ |
| *ni wis* [MB16] | N/A ([114] *huits*, flor) (*niwiy*, pleasant odor) | Teenek | Teenek | *Zamia fischeri* | Mexico-Tanute, Aquismón (San Luis Potosí) | -Narcotic |  |  | -Entheogen |  |  |
| *nimalari* [8] {q.v. *espadaña*} | ‘feather-leaf’ | Chiapanec | Chiapanec | *Dioon merolae* | Mexico-Suchiapa and Jiquipilas (Chiapas) |  |  |  |  |  |  |
| *o’k* [MB16] {q.v. *tzamaal*} | ‘head’ (ref. fem. cone) ([114] *ōc’* cabeza) | Teenek | Teenek | *Dioon edule* | Mexico-Aquismón (San Luis Potosí) |  |  |  |  |  |  |
| *o’tlab* (*o’tol*) [MB16] {q.v. *tzamaal*} | ‘shell’ (ref. sarcotesta) ([114] *ot’lab*, skin, shell) | Teenek | Teenek | *Dioon edule* | Mexico-Aquismón (San Luis Potosí) |  |  |  |  |  |  |
| *olote de konlif* [MB16] (*olotito*) {q.v. *konlif*} | ‘*konlif’s* ear’ (ref. male cone) | Spanish+ Teenek | Teenek | *Ceratozamia latifolia* | Mexico-Aquismón (San Luis Potosí) |  |  |  |  |  |  |
| *padre del elote* [MB16] {q.v. *tzompoyo* and *teocintle*} | ‘father of maize ear’ | Spanish | Nahua | *Ceratozamia fuscoviridis,*  *Zamia loddigesii* | Mexico-Huautla (Hidalgo) |  |  |  |  |  |  |
| *palma* | ‘palm’ | Spanish | Mestizo and Indigenous | various | widespread |  |  |  |  |  |  |
| *palma bendita* [MB16] | ‘blessed palm’ (ref. Palm Sunday palm) | Spanish | Otomí-Ñuhu | *Ceratozamia fuscoviridis* | Mexico (Hidalgo) |  |  |  | -Holy Week (*Semana Santa*) |  |  |
| *palma bola* [22] | ‘drunk palm’ | Spanish | Mestizo | *Zamia furfuracea* | Mexico (Veracruz) |  |  |  |  |  |  |
| *palma de [Santa] Teresita* [31,111] | ‘Saint Therese’s palm’ | Spanish | Mestizo | *Dioon edule* | Mexico (Veracruz) |  |  |  | -Patron saints' days |  |  |
| *palma de chicle* [22] {q.v. *chicalito*} | ‘rubber palm’? {or q.v. translation for *chicalito*} | Spanish | Mestizo?, Chinanteco? | *Dioon spinulosum* | Mexico (Oaxaca, Veracruz) |  |  |  |  |  |  |
| *palma de coyote* [MB08] | ‘coyote palm’ | Spanish | Zapotec | *Dioon* sp. | Mexico-San Jerónimo Taviche, Ocotlán (Oaxaca) |  |  | -Intentionally protected within traditional landscape management systems | -Holy Week (*Semana Santa*) |  |  |
| *palma de Dolores* [*dolores?*] [141] | ‘palm [that causes] suffering’ or ‘palm of Our Lady of Sorrows’ | Spanish | Mestizo? | *Dioon spinulosum, Dioon edule* | Mexico (Oaxaca, Veracruz) |  |  |  | -Patron saints’ days |  |  |
| *palma de la Vírgen* (*palmilla, palmita*) [142,143,144] | ‘Virgin Mary’s palm’ | Spanish | Guarijio [142,143] Mestizo | *Dioon holmgrenii, Dioon sonorense, Dioon stevensonii,*  *Dioon tomasellii* | Mexico-Tecolotlán (Jalisco) [MB16], Pochutla area (Oaxaca) [B Schutzmann pers. comm.], Durango [144]  San Luis Potosí, Sinaloa [130], southern Sonora [144] and [143], Guerrero | -Narcotic | -‘in market’ [130] | -Cultivated in home gardens as ornamental | -Day of the Dead (Nov. 2, Día de los Muertos)  -Patron saints’ days  -Virgin Guadalupe’s Day (12 Dec.) | -For baskets?  -Medicinal: Seeds ground, paste applied to eye, treatment for soreness (Guirocoba, Sonora) [143] |  |
| *palma de macetas* [130] | ‘flowerpot palm’ | Spanish | Mestizo | *Dioon tomasellii* | Mexico (Durango) |  |  |  |  |  |  |
| *palma de oro* [115] | ‘gold palm’ | Spanish | Chontal de Oaxaca | *Dioon merolae* | Mexico-Chontal region  (Oaxaca) |  |  |  |  |  |  |
| *palma de panteón* [63] | ‘cemetery palm’ | Spanish | Mestizo | *Dioon edule/ Dioon*  *angustifolium* | Mexico-Northeastern region |  |  |  | -Suggested by name (unconfirmed) |  |  |
| *palma de ramos* [MB16] | ‘Palm Sunday palm’ | Spanish | Otomí-Ñuhu | *Ceratozamia fuscoviridis* | Mexico (Hidalgo) |  |  |  | -Holy Week (*Semana Santa*) |  |  |
| *palma de serrucho* [22] | ‘[hand] saw palm’ (ref. jagged leaf edges) | Spanish | Mestizo | *Dioon edule* | Mexico-N/A |  |  |  |  |  |  |
| *palma de[l] Diablo* [145] | ‘Devil's palm’ | Spanish | Mestizo | *Dioon tomasellii* | Mexico-Río Grande Santiago basin region (Jalisco) |  |  |  | -Suggested by name (unconfirmed) |  |  |
| *palma de[l] monte* {q.v. *teocintle*} [45] | ‘forest palm’ | Spanish | Mestizo | *Ceratozamia*  *fuscoviridis* | Mexico-Quetzaltzongo, Tlanchinol (Hidalgo) |  |  |  |  |  |  |
| *palma del cerro* (*palmita*) [MB16] | hill palm | Spanish | Mestizo | *Dioon tomasellii* | Mexico-Chacala, Cabo Corrientes (Jalisco) |  |  |  |  |  |  |
| *palma del Maam* (*palma de Nuestro Señor*) [MB16] [44] {q.v. *konlif*} | ‘*Maam's* palm’ (ref. thunder god) | Spanish-Teenek | Teenek | *Zamia fischeri* | Mexico-Tanute, Aquismón  (San Luis Potosí) |  |  |  |  |  |  |
| *palma espinosa* (*palma*) | ‘spiny palm’ | Spanish | Mestizo? | *Ceratozamia sp.* | Mexico (Chiapas) |  |  |  |  |  |  |
| *palma espinuda* [112,139] | ‘spiny palm’ | Spanish | Mestizo,  Indigenous? | *Dioon merolae* | Mexico-Sierra Madre del Sur (Oaxaca) |  |  |  |  |  |  |
| *palma fúnebre* [146] | ‘funeral palm’ | Spanish | Mestizo | various *Zamia* species | Mexico-N.A. |  |  |  | -Suggested by name (unconfirmed) |  |  |
| *palma imperial* [31,147] | ‘imperial palm’ | Spanish | Mestizo | *Ceratozamia*  *‘robusta’ / ’mexicana’ = C. subroseophylla* | Mexico-Los Tuxtlas  (Veracruz) |  |  | -As pesticide |  |  |  |
| *palma real* | ‘royal palm’ | Spanish | Mestizo,  Indigenous | *Ceratozamia fuscoviridis* [45], *Dioon caputoi, Dioon sp. ‘San Jerónimo’* [MB08], *Dioon purpusii, Dioon rzedowskii* | Mexico-Quetzaltzongo, Tlanchinol (Hidalgo [45]) (Oaxaca [MB08], Puebla [111]) |  |  |  |  |  |  |
| *palma sol* [MB17] (*palma del sol, palmera del sol* [41,139,148]) | ‘sun palm’ | Spanish | Mestizo, Chatino, Zapotec | *Dioon holmgrenii* | Mexico-San Gabriel Mixtepec  (Oaxaca) |  |  | -Intentionally protected within traditional landscape management systems  --Intentionally managed for in traditional landscapes  -Co-managed with cattle | -Patron saints’ days  -Day of the Virgin of Guadalupe (Dec. 12)  -Holy Week (*Semana Santa*) |  |  |
| *palma teosinte* [51] {q.v. *tiusinte*} | ‘teosinte palm’ | Spanish+  Nahua | Mestizo | *Dioon mejiae* | Honduras |  |  |  |  |  |  |
| *palmeritas* [121] | ‘little palms’ | Spanish | Mestizo? | *Ceratozamia* spp. |  |  |  |  |  |  |  |
| *palmiche* [31,149] | ‘little palm’ | Spanish | Mestizo | Various, including *Zamia paucijuga* [107] | Mexico-widespread, including Manantlan Biosphere Reserve (Colima, Jalisco) |  |  |  |  | -Medicinal: stem used in childbirth, Moloacán, Veracruz |  |
| *palmilla* [31,19,112,113] | ‘little palm’ | Spanish | Mestizo,  Indigenous | Various, including *Ceratozamia huastecorum* [113], *Dioon* sp. [112], *Dioon califanoi* [19], *Zamia splendens* [116,117] | Mexico-widespread. El Ocote Biosphere Reserve (Chiapas); Sierra Juárez region (Oaxaca) [112] |  |  |  | -Christmas  -Holy Week (*Semana Santa*) | -Weddings |  |
| *palmilla de espina* [MB08] {q.v. *[x]yazn-goag*} | ‘little spiny palm’ | Spanish | Zapotec | *Dioon holmgrenii* | Mexico-San Isidro, Santiago Textitlan (Oaxaca) |  |  |  |  |  |  |
| *palmilla de la Sierra* (*palmita de la Sierra*) [MB16] {q.v. *tzamaal*} | ‘little Sierra palm’ | Spanish | Teenek | *Dioon edule* | Mexico-Aquismón (San Luis Potosí) |  |  |  |  |  |  |
| *palmita* (*palmito, palmitón* [MB16]) [22,31,144,150] | ‘little palm’ | Spanish | Mestizo,  Indigenous | Various. *Ceratozamia miqueliana* [22], *Dioon tomasellii* [144], *Zamia inermis* [22], *Zamia loddigesii* [150] | Mexico-widespread. (Guerrero [144], Michoacán, Nayarit, Oaxaca, Jalisco |  |  |  |  | -Decorative: floral arrangements for celebrations (Tequestitlán, Jalisco) |  |
| *palmita de la Vírgen* [44] | ‘little palm of the Virgin Mary’ | Spanish | Teenek | *Dioon edule* | Mexico-Tancanhuitz de Santos (San Luis Potosí) |  |  |  | -Yes (unspecified) |  |  |
| *palmito de cerro* [MB16] | ‘little hill palm’ | Spanish | Mestizo | *Zamia paucijuga* | Mexico-El Teocinte, Tomatlán (Jalisco) |  |  | -Cultivated in gardens |  |  |  |
| papelillo [52] {q.v. tiusinte} | ‘little paper’ (ref. endotesta) | Spanish | Mestizo | *Dioon mejiae* | Honduras |  |  |  |  |  |  |
| *pata de gallo* [54] | ‘rooster's claw’ (ref. leaf shape) | Spanish | Mestizo | *Ceratozamia hildae* | Mexico (San Luis Potosí) |  |  |  |  |  |  |
| *peine* (*peinetillo*) [144,145] | ‘comb’ (‘little comb’) (ref. male cone) | Spanish | Mestizo/  Indigenous | *Dioon sonorense, Dioon tomasellii* | Mexico (Nayarit [MB16]),  (Sinaloa, central Sonora [22,144,145] | -Narcotic? |  |  |  | -As comb? (suggested by name) |  |
| *pesma espinosa* [151] | ‘spiny fern’ | Spanish | Mestizo/Indigenous | *Ceratozamia fuscoviridis,* possibly other *Ceratozamia* | Mexico (Veracruz) |  |  |  |  |  |  |
| *peyote* (*palma peyote*) [22,AV00,MB16] {q.v. *palma de la Vírgen*} | ‘white hairs’ (ref. directly to peyote cactus *Lophophora williamsii*, or to tomentum on fem. cone) | Nahua | Mestizo, Cora, Yaqui | *Dioon sonorense, Zamia paucijuga* | Mexico (Sonora [22,AV00]  (Nayarit [MB16]) | -Narcotic (stem, root-distilled as *sotol*) |  |  | -Entheogen? | -Medicinal: for joint pain (‘tullidos’), drink water derived from trunk starch; cone mucilage applied as poultice (*D. sonorense*) |  |
| *pileloltsi* (*pilololcintli*) [44] {q.v. *teocintle* and *tzompoyo*} | ‘small maize ears’ | Nahua | Nahua | *Ceratozamia*  *fuscoviridis,*  *Zamia loddigesii* | Mexico-Huautla (Hidalgo) |  |  |  |  |  |  |
| *piña* | ‘pineapple’ (ref. fem. cone) | Spanish | Mestizo | various | Mexico-widespread |  |  |  |  |  |  |
| *piña del monte* [31] | ‘forest pineapple’ | Spanish | Mestizo/  Indigenous | various *Zamia* and *Ceratozamia* | Mexico-widespread |  |  | -As pesticide (*Ceratozamia mexicana*) [31] |  |  |  |
| *planta del Diablo* [44] | ‘Devil’s plant’ | Spanish | Teenek | *Dioon edule* | Mexico-Aquismón (San Luis Potosí) |  |  |  |  | -Connected explicitly to sense of place |  |
| *planta dueña de la tierra* [MB16] | ‘plant that owns the land/earth’ | Spanish | Teenek | *Dioon edule* | Mexico-Aquismón (San Luis Potosí) |  |  |  |  | -Connected explicitly to sense of place |  |
| *plumilla* [41,139,148]{q.v. palma sol} | ‘little feather’ (ref. tomentum on young leaf) | Spanish | Mestizo,  Chatino, Zapotec | *Dioon holmgrenii* | Mexico-San Gabriel Mixtepec (Oaxaca) |  |  |  |  |  |  |
| *po’jodh* [MB16a] {q.v. *konlif*} | ‘pollen’ ([114] *pojoth*, polvo) | Teenek | Teenek | *Ceratozamia*  *latifolia* | Mexico-Aquismón (San Luis Potosí) |  |  |  |  |  |  |
| *po’jodh tzamaal inik* (*pojostlil tsamal inik*) [44,MB16] {q.v. tzamaal} | ‘male chamal pollen’ | Teenek | Teenek | *Dioon edule* | Mexico-Aquismón (San Luis Potosí) |  |  |  |  |  |  |
| *poua* [111] {q.v *maíz de coxca*} | N/A | Zoque | Zoque | *Zamia loddigesii* | Mexico (S Veracruz) |  |  |  |  |  | Source records as of ‘Zoque-Popoluca’ origin; may not be reliable |
| *s+Njul’ gus+I* [73] {q.v. *dameu*} | ‘shirt,’ ‘covering’ (ref. sarcotesta and/or sclerotesta) | Xi’iuy | Xi’iuy | *Dioon edule* | Mexico-Sierra Gorda region  (San Luis Potosí, Queretaro) |  |  |  |  |  |  |
| *San Anselmo* [53] | ‘Saint Anselm’ | Spanish | Mestizo,  Indigenous? | *Zamia sp. 'furfuracea' (*presumably *Z. prasina)* | Mexico-Buenavista Xbac  (Yucatan) |  |  |  | ? |  |  |
| *sintli de cuautitla* [44] {q.v. *tzompoyo* and *teocintle*} | ‘forest maize ear’ | Nahua+ Spanish | Nahua | *Zamia loddigesii* | Mexico-Huautla  (Hidalgo) |  |  |  |  |  |  |
| *sotol* [130,AV00] {q.v. *peyote*} | ref. either ‘*zotolin’* (palm), or ‘*sotol*’ (*Dasylirion* spp. often used in ceremonial decorations with cycads, and also alcoholic drinks made from various starch-bearing plants; Sonora usage is to alcoholic drink) | Nahua | Mestizo | *Dioon angustifolium*, *Dioon sonorense* | Mexico (Sonora; Tamaulipas) |  |  |  |  |  |  |
| *t’ichol* [MB16] {q.v. *tzamaal*} | ‘membrane’ (ref. endotesta) | Teenek | Teenek | *Ceratozamia latifolia* | Mexico-Huasteca region (San Luis Potosí) |  |  |  |  |  |  |
| *t’uul* [MB16] {q.v. *tzamaal*} | ‘the body’ (ref. sarcotesta) | Teenek | Teenek | *Dioon edule* | Mexico-Aquismón (San Luis Potosí) |  |  |  |  |  |  |
| *tamo* [49] {q.v. *tiusinte*} | (ref. smell of sun-dried seed starch) | Spanish | Mestizo | *Dioon mejiae* | Honduras |  |  |  |  |  |  |
| *tapacarbón* (*tapacapón, tapacarpón:* in error?) [115,146,151] | N/A | Spanish | Mestizo | *Ceratozamia matudae* | Mexico-Biosfera La Sepultura; Finca Prusia (Chiapas) |  |  |  |  |  |  |
| *tatatlcintli* [45] {q.v. *tzompoyo* and *teocintle*} | ‘father of maize ear’ | Nahua | Nahua | *Zamia fischeri,Zamia loddigesii* | Mexico-Tenango, Tlanchinol (Hidalgo) |  |  |  |  | -Thatch for roofs (leaves) (formerly) |  |
| *teocin[is]huatl* (*tiozin[is]huatl, tiosin[is]kuatl*) [44,45] {q.v. *teocintle*} | ‘sacred maize leaf’ | Nahua | Nahua | *Ceratozamia fuscoviridis* | Mexico-Sta. Ma. Tepecintla, Tlanchinol (Hidalgo) |  |  |  |  | - Thatch for roofs (leaves) (formerly) |  |
| *teocintle* (*[teu-, tio-, tiu-, tu-] + [-s-, -z-, -c-, -x-] + [-in-, -en-] + [-tle, -tli, -te, ti-]*) [44,45,46,52,152,153] {for Honduran usage, q.v. *tiusinte*} | ‘sacred maize ear’ | Nahua | Mestizo, Nahua | *Ceratozamia fuscoviridis,*  *Zamia fischeri,Zamia loddigesii* | Mexico (Hidalgo, San Luis Potosí) | -Atole (mixed with maize meal) (Tlanchinol, Hgo)  -Sarcotestas, raw  -Seeds, roasted (formerly, in Xochiatipan, Hidalgo [45])  -Tortillas from seeds (detox.: roasted; lime) (formerly) (meal sometimes mixed with maize meal) (possibly still eaten in Acahuasco, Tlanchinol, Hgo) |  | -As domestic animal feed (detox. seeds)  -Associated with maize  -Cultivated in home gardens  -Dioecy recognized  -Intentionally protected in traditional landscape management systems  -Leave for maize granary roofs  -Leaves for chicken coops and pigpens [46]  -Pesticide (*Z. fischeri*): Huitzotlaco, Atlapezco, seeds ground, mixed with maize meal, to kill mice and rats [46] | -Assumption (15 Aug. Día de la Ascensión)--roofs for food stalls in Sta Ma Tepetzintla, Tlanchinol and Molango, Hgo [45,46]  -Christmas Eve (roofs for houses in Nativity scenes) [44]  -Christmas, Molango, Hgo [46]  -Corpus Cristi, Molango, Hgo [46]  -Day of the Cross (3 May), Molango, Hgo [46]  -Day of the Dead (2 Nov.)  -El Chicomexochitl maize festival [38]: San Juan Huazalingo, Hgo= Michaelmas  -Holy Week (*Semana Santa*)  -Michaelmas maize festival (Día de San Miguel, 29 Sept.) [44]  -St. Bartholemy’s Day (24 Aug.), Molango, Hgo[46]  -St. Ignatius’ Day (31. Jul.), Molango, Hgo [46]  -St. Isidore’s Day (15 Mar), Molango, Hgo [46]  -St. John’s Day (24 Jun.)--*C. fuscoviridis* in Huazalingo, Hgo [45]  -St. Joseph’s Day (Día de San José, 19 Mar.) [45]  -St. Martin’s Day (11 Nov.), Molango, Hgo [46]  -Virgin Guadalupe Day (Dec. 12) [45,46] | -As toys (male cone): *Z. fischeri* [46]  -Decorative arches for important secular events such as weddings, graduations, Independence Day celebrations (leaves in shape of X), and the visits of dignitaries (all [45], Tlanchinol, Hidalgo)  -Medicinal: ‘látex’ gum from *Z. loddigesii* stems used to cure blisters caused by the *tazolmama* ‘carga basura’ insect; mashed seeds applied to skin help cure *xiyotl*, a rash (Pesmayo, Xochiatipan, Hidalgo [45])  -Medicinal (*Z. fischeri*): La Palma, Chapulhuacán, Hidalgo [46]; (*C. fuscoviridis*): Chichiltepetl, Tlanchinol, Hidalgo [46]  -Medicinal (*Z. fischeri*): seeds roasted, boiled, ground, and made into atole, flavored with orange leaves and cinnamon, given to the sick; toasted seeds ground, dry powder applied to smallpox blisters (formerly): (Huitzotlaco, Atlapezco [46])  -*Petates* (bedrolls) and *tortilleros* (tortilla holders) (Leaves)  -Thatch for roofs (for permanent constructions, mostly formerly; currently, for temporary shelters, such as resting places for farmers in maize fields) |  |
| *teosinte* [119] | ‘sacred maize ear’ | Nahua | Mestizo? | *Zamia ‘loddigesii’* (probably *Z. prasina*) | Guatemala-‘north coast, *fide* [Ulise]} Rojas’ |  |  | -Associated with maize (name) |  |  |  |
| *tepe[t]maízte* [154] | ‘hill maize’ | Nahua+  Spanish | Mestizo | *Ceratozamia morettii* | Mexico-Landero y Coss  (Veracruz) |  |  | -Associated with maize (name) |  |  |  |
| *tepexi* [TP17] | ‘hill seed,’ ‘hill maize ear,’ ‘hill leaves’ | Nahua | Nahua | *Ceratozamia totonacorum* | Mexico-Cuetzalán, Sierra Norte region (Puebla) |  |  | -Associated with maize (name) | -Yes (unspecified) |  |  |
| *tepezintle* (*teocintli* [66]*, tepecintle* [44,TP13,TP16]*, tepecintli* [66]*, tepetlcintle* [TP16] | ‘hill maize’ | Nahua | Nahua | *Ceratozamia fuscoviridis,*  *C. totonacorum* | Mexico-Huayacocotla (Veracruz); Sierra Norte region (Puebla);  Huazalingo [44] (Hidalgo) | -Roots, with maize, cooked (formerly) [66]  -Sarcotestas, raw (formerly) [66] |  | -Associated with maize | -Yes (unspecified) | -Medicinal: anti-diarrheal [66] (formerly) |  |
| *thimaloon poko* [MB16] {q.v. *konlif*} | ‘wild palm’ ([114] *thimalōn*, wild) | Teenek | Teenek | *Ceratozamia latifolia* | Mexico-Aquismón (San Luis Potosí) |  |  |  |  |  |  |
| *Thipaak* (*Dhipak*) [44,82] {q.v. *konlif*} | ‘*Thipaak’* (direct ref. deity) | Teenek | Teenek | *Ceratozamia latifolia* | Mexico-Huasteca region (San Luis Potosí) |  |  |  |  |  |  |
| *thokobil amam* (*thocobil amam, to kobil amam*) [44,MB16]{q.v. *konlif*} | ‘*Maam’s* palm’ (ref. thunder god) | Teenek | Teenek | *Zamia fischeri* | Mexico-Aquismón (San Luis Potosí) |  |  |  |  | -Influence on weather |  |
| *tiñúc* [51] | N/A (derived from *tiusinte*?) | Tolupan | Tolupan | *Dioon mejiae* | Honduras-Yoro (Yoro) | -Atole (formerly)  -Breads for seeds *(rosquillas*)  -Tamales and tortillas from seeds  -Detox. methods: ash, lime | -Take to local markets to sell to mestizos | -Associated with maize  -Intentionally protected within traditional landscape management systems | -Christmas Eve (Nativity scenes) | -Roofs for temporary shelters |  |
| *tío de la mazorca* [MB16] {q.v. *konlif*} | ‘maize’s uncle’ | Spanish | Teenek | *Ceratozamia latifolia* | Mexico-Aquismón (San Luis Potosí) |  |  |  |  |  |  |
| *tío del maíz* [44] {q.v. *teocintle* and *tzompoyo*} | ‘uncle of maize’ | Spanish | Nahua | *Ceratozamia fuscoviridis* | Mexico-Huazalingo (Hidalgo) |  |  |  |  |  |  |
| *tío del mono* [MB16] | ‘uncle of *mono*’ (ref. *C. latifolia*) | Spanish+ Teenek | Teenek | *Zamia fischeri* | Mexico-Aquismón  (San Luis Potosí) | -Narcotic |  | -Associated with maize | -Entheogen  -Incorporated into altars with offerings for maize ceremonies | -Medicinal (leaves, for ‘*granos’*) |  |
| *tiotamal* (*quiotamal*) [31,63] {q.v *chamal* for related uses in NEMX) [B. Schutzmann, pers. comm.] | ‘sacred tamale’ ([141] mistranslates ‘Tio Tamal’ as ‘Uncle Tamal’) | Nahua | Mestizo | *Dioon edule* | Mexico  (Veracruz) | -Seeds (formerly) (Monte Oscuro, Veracruz) |  | -Associated with maize? | -Cultivated in home gardens as ornamental | -As toys: sarcotestas as ‘*rezumbadores*’)  -Seed starch used for ironing clothes, Pinotepec, Emiliano Zapata, Veracruz ([63] ref. to J. Rees herbarium voucher note) |  |
| *tiusinta* (*teocinta, etc.*) [52] {q.v. *tiusinte*} | ‘female tiusinte plant’ | Nahua+  Spanish | Mestizo, Nahoa de Honduras | *Dioon mejiae* | Honduras |  |  |  |  |  |  |
| *tiusintal* (*tiusintalito, tiusintillal, teocintal, etc.*) [52]{q.v. *tiusinte*} | ‘*tiusinte* population’ | Nahua+  Spanish | Mestizo, Nahoa de Honduras | *Dioon mejiae* | Honduras |  |  |  |  |  |  |
| *tiusinte* (*palmera, palma teosinte, tiusintillo*) {for Mexican usage, q.v. *teocintle*} [51,52,49,91,MB03-16] (spelling variants as for *teocintle*; most common is *teocinte*) | ‘sacred maize ear’ | Nahua | Mestizo,  Nahoa de Honduras | *Dioon mejiae* | Honduras | -Tamales and tortillas from seeds  -Atoles (‘*bebidita de tiusinte*,’ ‘*horchata*,’ ‘*pinol*’)  -‘Mascaduras’ (‘*panes,*’ ‘*rosquillas*,’ ‘*rosquetes*,’ ‘*semitas*,’: bread products made with wheat, maize, and sometimes cheese, yeast, and eggs)  -Detox. methods: ash (preferred), lime | -Sold by harvesters directly to users (seeds)  -Sold in local markets (seeds, tamales from seeds, leaves)  -Presence in long-distance exchange networks (formerly)  -Bartered for other producs | -Associated with maize  -Co-managed with cattle  -Cultivated in home gardens  -Dioecy recognized  -Existence of planted populations  -Fed to chickens (detox. seeds), cattle (formerly)  -Intentionally managed for in traditional landscapes systems  -Intentionally protected in traditional landscape management  -Pollination mechanisms recognized | -Christmas Eve (Nativity scenes, church facades)  -Day of the Dead (2. Nov.)  -Holy Week (*Semana Santa*)  -Individual death rites  -Patron saints' days  -Planted in cemeteries | -As toys: whistles, pin-and-targets (‘*enchutes*’)  -Brooms (leaves) (Guata, Olancho)  -Connected explicitly to sense of place -Crafts (various cone parts, for figures of deer) (Guata, Olancho)  -Decorative (leaves for weddings, bishops’ visits)  -Decorative (plants often grown in public parks and gardens)  -Glue for sealing envelopes (female cone resin) (formerly)  -Medicinal: as decongestant, female cone mucilage and tomentum mixed with Baird’s tapir (*Tapirus bairdii*) lard (Esquipulas del Norte, Olancho, formerly); tomentum used as cottonsubstitute for wounds (Guata, Olancho)  -Medicinal: mucilage for poultice (Guata and Esquipulas del Norte, Olancho)  -Thatch for temporary shelters |  |
| *tiusintera* (*teocintera, etc.)* [52] {q.v. *tiusinte*} | ‘*tiusinte* food preparer’ (ref. woman) | Nahua+ Spanish | Mestizo, Nahoa de Honduras | *Dioon mejiae* | Honduras |  |  |  |  |  |  |
| *tiusintero* (*teocintero, etc.*) [52] {q.v. *tiusinte*} | ‘*tiusinte* gatherer’ (ref. man) | Nahua | Mestizo, Nahoa de Honduras | *Dioon mejiae* | Honduras |  |  |  |  |  |  |
| *toro* [MB16] {q.v. *konlif*} | ‘bull’ (ref. phallic shape of male cone) | Spanish | Teenek | *Ceratozamia*  *latifolia* | Mexico-Aquismón (San Luis Potosí) |  |  |  |  |  |  |
| *ts’een Thipaak* [82] {q.v. *konlif*} | ‘sierra *Dhipak*’ ([70] *ts’ēn*, mountains) | Teenek | Teenek | *Ceratozamia*  *latifolia* | Mexico-Huasteca region (San Luis Potosí) |  |  |  |  |  |  |
| *tsakam Thipaak* [82] {q.v. *konlif*} | ‘little Dhipak’ | Teenek | Teenek | *Ceratozamia*  *latifolia* | Mexico-Huasteca region (San Luis Potosí) |  |  |  |  |  |  |
| *tsakam way’* [82] {q.v. *konlif*} | ‘child maize ear’ ([70] *huay’*, maize ear) | Teenek | Teenek | *Ceratozamia*  *latifolia* | Mexico-Huasteca region (San Luis Potosí) |  |  |  |  |  |  |
| *tsalam Thipaak (tzalam-thipac)* [82] {q.v. *konlif*} | ‘*Dhipak’s* shade’ | Teenek | Teenek | *Ceratozamia*  *latifolia* | Mexico-Huasteca region (San Luis Potosí) |  |  |  |  |  |  |
| *tsubal* [44] {q.v. *konlif*} | ‘life of maize’ | Teenek | Teenek | *Ceratozamia*  *latifolia* | Mexico-Huasteca region (San Luis Potosí) |  |  |  |  |  |  |
| *tullidora* [111] | ‘crippler’ | Spanish | Mestizo | *Dioon edule* | Mexico-N/A |  |  |  |  |  |  |
| *turhaa* [MB16] | N/A | Cora | Cora | *Dioon tomasellii* | Mexico-Sierra Madre Occidental, Cora region (Nayarit) |  |  |  | -Christmas Eve  -Holy Week (*Semana Santa*) |  |  |
| *tush-kjù* [41,112,155,156] | ‘seedling-guardian’ [157] | Mazatec | Mazatec | *Dioon purpusii, Dioon rzedowskii* | Mexico—San Bartolomé Ayautla, Río Santo Domingo valley; Santa Catarina (Oaxaca) |  |  | -Associated with maize (name) |  |  |  |
| *tushku* [11] | N/A (*tus* and *kun*/*kum*, both types of palms; or mistransliteration of *txök-k'i'ix* {q.v.}) | ‘Tzeltal,’ probably Lacandon | ‘Tzeltal,’ probably Lacandon | *Ceratozamia ‘robusta’* or *Zamia sp. (Z. lacandona?)* | Mexico-Selva Lacandona region (Chiapas) | -Seeds and sarcotestas  -Detox. method: boiled or steamed in *tequixquitl* (‘*tierra salitrosa con sales de calcio, potasio, sodio*’) |  |  |  |  | Source is confused and problematic |
| *txök-k'i'ix* [140] | ‘red spine’ | Lacandon | Lacandon | *Zamia sp.* (*lacandona*?) wrongly identified as *‘skinneri’* | Mexico (Chiapas) |  |  |  |  |  |  |
| *tzamaal* (*tsamal, tsamalib, tsamay, tzamal, tzama[a]lib, tzamay*) [MB16,158]{for Mestizo and Nahua uses, q.v. *chamal*} (*tzamalib* cited as ‘true Teenek name’) | ‘plant of cold land’ (ref. populations at higher altitude in the mountains above the Huasteca lowlands) ([114] *tsamāy*, the cold) | Teenek | Teenek | *Ceratozamia latifolia,*  *Dioon edule* | Mexico-Huasteca  (San Luis Potosí); La Cercada, Valle Verde (Queretaro) [158] | -Detox. methods: boiling only; lime; ash  -Fried meal, in strips, sometimes sweetened  -Sarcotestas, raw  -Stem starch steamed  -Tamales (spherical ‘*bolas’* or rectangular ‘*cuadrado’*; sometimes mixed with maize meal)  -Tortillas from sarcotesta (raw)  -Tortillas from seeds  (all formerly in San Luis  Potosí; reported as current in Queretaro [158]) | -Sold by harvesters directly to users (seeds and tamales)  -Sold in local markets  -Presence in long-distance exchange networks  (all formerly) | -Associated with maize  -Grown in home gardens for use in religious ceremonies | -Used by shamans (primarily Nahua) for curses and counter-curses  -All Saints’ Day (2 Nov. *Todos Santos*)  -Ash Wednesday (beginning of Lent, *Cuaresma*)  -Christmas Eve  -Virgin of Guadalupe’s Day (12 Dec.) |  |  |
| *tzi’tzí:ks* [159] | N/A (*tzintziqui t’uhuán*, ‘fern,’ [160], *t’uhuán*, leaf) | Totonac | Totonac | 'Cycad’-unidentified *(Ceratozamia totonacorum*?) | Mexico-Necaxa valley, Sierra Norte region (Puebla) |  |  |  |  | -‘light, strong wood used for house posts’ |  |
| *tzikare’e* [MB16] {q.v. *turhaa*} | ‘spiny plant’ [161] | Cora | Cora | *Dioon tomasellii* | Mexico-Sierra Madre Occidental region (Nayarit) |  |  |  |  |  |  |
| *tzilacapame* [45] | (ref. *Eumaeus* caterpillar) | Nahua | Nahua | *Zamia loddigesii* | Mexico-Xochiatipan (Hidalgo) |  |  |  |  |  |  |
| *tzompoyo* (*[tz-, ts-, ch-] + [-um-, -om-] + [-poyo, -pollo, puyo, pullo]*) [45] [MB16] [44] (*guachumpoyó, cuachumpoyó* ‘cerca de Huichapan Hgo’) [115] {q.v. *teocintle* for additional uses} |  | Nahua | Nahua | *Ceratozamia fuscoviridis,*  *Zamia fischeri,Zamia loddigesii* | Mexico (Hidalgo) | -Tortillas from stems (detox. method: lime) (formerly)  -Narcotic |  | -Associated with maize  -Dioecy recognized  -Intentionally protected within traditional landscape management systems  -Pollination mechanisms recognized |  |  |  |
| *tzukix* (*tsuquix*) 62,163] (formerly?) | N/A (*txök-k'i'ix*, ‘red spine’ [140] Lacandón Maya {q.v }) | N/A (Popoluca?) | N/A (Popoluca?) | *Zamia ‘spartea’ (=Zamia*  *loddigesii)* | Mexico-Acayucán (Veracruz) |  |  |  |  | -Medicinal: snakebite remedy (formerly?) |  |
| *ucuileme* [MB16] {q.v. *tzompoyo* and *teocintle*} | ‘caterpillar’ (ref. *Eumaeus* spp.) | Nahua | Nahua | *Zamia loddigesii* | Mexico (Hidalgo) |  |  |  |  |  |  |
| *uj-yazngoa* (*uy yasngoa, uj ya goo*) [MB08] {q.v. *[x]yazn-goag*} | ‘seed of spiny tree with camote,’ ‘palm seed’ (l.i.) | Zapotec | Zapotec | *Dioon holmgrenii* | Mexico-San Isidro, Santiago Textitlán (Oaxaca) |  |  |  |  |  |  |
| *ushu[m] konlif* (*uxum conlif*) [44] {q.v. *konlif*} | ‘female konlif’ (ref. fem. cone) | Teenek | Teenek | *Ceratozamia latifolia* | Mexico-Aquismón (San Luis Potosí) |  |  |  |  |  |  |
| *ushum tzamaal* [MB16] {q.v. *tzamaal*} | ‘female tzamaal’ | Teenek | Teenek | *Ceratozamia latifolia* | Mexico-  Aquismón  (San Luis Potosí) |  |  |  |  |  |  |
| *uvecito* [MB08] {q.v. *[x]yazn-goag*} | ‘little grape’ (ref seed) | Spanish | Zapotec | *Dioon holmgrenii* | Mexico-Santiago Textitlán  (Oaxaca) |  |  |  |  |  |  |
| *vano* [MB16] [52] (q.v. *grano vano*) | ‘in vain’ (ref. seed that doesn’t grow/ma-ture) | Spanish | Mestizo, Teenek | *Dioon edule, Dioon mejiae* | Honduras; Mexico-Aquismón  (San Luis Potosí) |  |  |  |  |  |  |
| *vestimento* [52] {*q.v. tiusinte*} | ‘outfit’ or ‘dress’ (ref. leaf flush) | Spanish | Mestizo | *Dioon mejiae* | Honduras-Gualaco |  |  |  |  |  |  |
| *xachitza* (*xihci, dashitzi*) [MB16] | ‘diminishes the rain’ (*xa, xats’i*) | Otomí-Ñuhu | Otomí-Ñuhu | *Ceratozamia fuscoviridis* | Mexico(Hidalgo) |  |  |  |  | -Influence on the weather |  |
| *xiuiyo* [45] {q.v. *teocintle*} | ‘leaf’ | Nahua | Nahua | cycad spp. | Mexico (Hidalgo) |  |  |  |  |  |  |
| *xochitl mozoyawa shiwiti* [44] {q.v. *teocintle*} | ‘flowering plant’ | Nahua | Nahua | *Ceratozamia fuscoviridis,*  *Zamia loddigesii* | Mexico (Hidalgo) |  |  |  |  |  |  |
| *ya’a goo xhil* (*ya’a xhil goo*) [MB08,164,165] | Indicates morphological relationships to *Dioscorea* and *Manihot*, and grouped within palms [165] | Zapotec | Zapotec | *Ceratozamia sp. ‘mexicana’* [164], *‘Ceratozamia aff. longifolia* Miq*.’* [165] | San Isidro del Camino, Candelario Loxicha [MB08]; San Agustín Loxicha [164]: Sierra Sur region (Oaxaca) |  |  |  | -Yes (unspecified) [164] | -Decorative: leaves for arch at town entrance, 15 May festival [MB08]  -Insectide (slurry from seeds, mixed with jelly to attract and poison flies) |  |
| *ya-tuj-cho* (*Yahtuchó* [132], *ya-tuj-cho-chu:*female*; ya-tuj-cho-shi-i:* male) [MB08] [132] {q.v. *tush-kjú*} | ‘tree-seedling-guardian’ [157] | Mazatec | Mazatec | *Dioon spinulosum* | Mexico-Cerro Tepezcuintle, Tuxtepec (Oaxaca) | (formerly) |  | -Associated with maize  -Cultivated in home gardens  -Dioecy recognized | -Yes (unspecified religious festivals) | -As toys (sclerotestas as whistles)  -Crafts (unspecified) |  |
| *yerba sagrada* [8] {q.v. *espadaña*) | ‘sacred plant’ | Spanish | Chiapanec | *Dioon merolae?* | Mexico-Suchiapa (Chiapas) |  |  |  | (formerly)-likely reference to this species |  |  |
| *yojrá unicej* [51] | ‘poisonous manioc’ | Pech | Pech | *Zamia standleyi* | Honduras-San Esteban  (Olancho) | -Stem (detox.: washed, cooked, then fried in strips) (formerly) |  | -Pollination mechanisms recognized (‘*polvillo tóxico*’) |  |  |  |
| *yuca de ratón* [51] {q.v. *mata ratón*} | ‘rat manioc’ (ref. use as pesticide) | Spanish | Mestizo,  Tolupan | *Zamia sandovalii, Zamia standleyi,* | Honduras-Yoro (Yoro) |  |  | -As rodenticide (mashed stem starch) |  | -Starch production (stem grated and baked in oven): for clothes  -Medicinal: cooked starch with lemon, to treatment bloody diarrhea in children |  |
| *zaa korona yazngoa* [MB08] {q.v. *[x]yazn-goag*} | ‘crown of spiny tree with *camote*’ (l.i.) (ref. leaf crowns fashioned for Holy Week) | Zapotec | Zapotec | *Dioon holmgrenii* | Mexico-San Isidro, Santiago Textitlan (Oaxaca) |  |  |  |  |  |  |

**Table S1 key**

^a^Parentheses: alternate names. Words in brackets: alternate spellings. Capital letters and diacritical marks as in original texts and transliterations by informants. ‘+’ and ‘-‘ used to indicate compound spellings (for example, for the many variants of *teocintle*). Transcriptions from oral accounts follow orthography given by interviewees, where provided, and/or orthography in accordance with dictionaries and other sources consulted. Numbers in brackets: references in bibliography; letter-number combinations indicate unpublished fieldnotes (author, date – e.g., MB08 refers to Mark Bonta’s 2008 notes). Where no references are cited, term is widespread throughout the region. ^b^Where available: Written down for the authors by local informants (l.i.); in published sources; dictionary translations proferred by authors. Ref.=refers to (where not obvious from name). If ‘ref.’ not listed, then refers to whole plant. “?” indicate potential translations of terms. ^c^Language/s or language group/s utilizing term. ‘+’=compounds of two languages. ^d^Ethnic group/s utilizing term/s. ^e^Format: Country-Town, Municipality or Region (State or Province). ^f^Unless otherwise indicated, religious uses involve leaves.

**Table S2: Ethnobotany of MNCA cycads by species**

|  |  |  |  | **MAJOR USES and BELIEFS** | | | | | | | | | | | | | | | | | | | | **MINOR USES and BELIEFS**^b^ | | |  | | |  | | |  | | | | | | | | | | | | | | | | | | | | | | | | | |  |
| --- | --- | --- | --- | --- | --- | --- | --- | --- | --- | --- | --- | --- | --- | --- | --- | --- | --- | --- | --- | --- | --- | --- | --- | --- | --- | --- | --- | --- | --- | --- | --- | --- | --- | --- | --- | --- | --- | --- | --- | --- | --- | --- | --- | --- | --- | --- | --- | --- | --- | --- | --- | --- | --- | --- | --- | --- | --- | --- | --- |
| **SPECIES**^a^ | **RANGE** | **IUCN^c^** | **NAME**^d^ | **Alimen-tary** | | **Maize associ-ation** | | | **Econo-mic** | | | **Agro-ecological** | | | | **Religi-ous** | | **Medi-cinal** | | | **Deco-rative Non-religious** | | | **As toys** | | | **Sense**  **of place** | | | **Weather** | | | **Assas-sination** | | | **Cons-truction** | | | **Narco-tic** | | | **Enthe-ogen** | | | **Dye/Co-loring**  **Agent** | | | | **Combs** | | **Wea-ving** | | | **Laun-dry Starch** | | | **Enve-lope Glue** | |  |
| **Ceratozamia alvarezii** Pérez-Farr., Vovides & Iglesias | Mexico (Chiapas) | EN | x | x? | | | |  | | |  | | |  | | |  | | |  | | |  | | |  | |  | | | |  | | |  | | |  | |  | | |  | | | |  | | |  | | |  | | |  | |  | |
| **Ceratozamia brevifrons** Miq. | Mexico (Veracruz) | VU |  |  | | | |  | | |  | | |  | | |  | | |  | | |  | | |  | |  | | | |  | | |  | | |  | |  | | |  | | | |  | | |  | | |  | | |  | |  | |
| **Ceratozamia chamberlainii** Mart.-Domínguez, Nic.-Mor. & D.W.Stev. | Mexico (Hidalgo, Querétaro, San Luis Potosí) | NE | x | x? | | | | x | | |  | | |  | | |  | | |  | | |  | | |  | |  | | | |  | | |  | | |  | |  | | |  | | | |  | | |  | | |  | | |  | |  | |
| **Ceratozamia chimalapensis** Pérez-Farr. & Vovides | Mexico (Oaxaca) | CR | x | x | | | |  | | |  | | | x | | |  | | |  | | |  | | |  | |  | | | |  | | |  | | |  | |  | | |  | | | |  | | |  | | |  | | |  | |  | |
| **Ceratozamia decumbens** Vovides, Avendaño, Pérez-Farr. & Gonz.-Astorga | Mexico (Veracruz) | CR |  |  | | | |  | | |  | | |  | | |  | | |  | | |  | | |  | |  | | | |  | | |  | | |  | |  | | |  | | | |  | | |  | | |  | | |  | |  | |
| **Ceratozamia delucana** Vázq.Torres, A.Moretti & Carvajal-Hern. | Mexico (Veracruz) | n.a. |  |  | | | |  | | |  | | |  | | |  | | |  | | |  | | |  | |  | | | |  | | |  | | |  | |  | | |  | | | |  | | |  | | |  | | |  | |  | |
| **Ceratozamia euryphyllidia** Vázq.Torres, Sabato & D.W.Stev. | Mexico (Oaxaca, Veracruz) | CR |  |  | | | |  | | |  | | |  | | |  | | |  | | |  | | |  | |  | | | |  | | |  | | |  | |  | | |  | | | |  | | |  | | |  | | |  | |  | |
| **Ceratozamia fuscoviridis** W.Bull | Mexico (Hidalgo, Veracruz) | CR | x | x | | | | x | | |  | | | x | | | x | | | x | | | x | | |  | |  | | | | x | | |  | | | x | |  | | |  | | | |  | | |  | | | x | | |  | |  | |
| **Ceratozamia hildae** G.P.Landry & M.C.Wilson | Mexico (Querétaro, San Luis Potosí) | EN | x |  | | | | x | | |  | | |  | | |  | | |  | | |  | | |  | |  | | | |  | | |  | | |  | |  | | |  | | | |  | | |  | | |  | | |  | |  | |
| **Ceratozamia hondurensis** J.L.Haynes, Whitelock, Schutzman & R.S.Adams | Honduras (Atlántida) | CR | x |  | | | |  | | |  | | | x | | |  | | |  | | |  | | |  | |  | | | |  | | | x | | |  | |  | | |  | | | |  | | |  | | |  | | |  | |  | |
| **Ceratozamia huastecorum** Avendaño, Vovides & Cast.-Campos | Mexico (Veracruz) | CR | x |  | | | |  | | |  | | |  | | |  | | |  | | |  | | |  | |  | | | |  | | |  | | |  | |  | | |  | | | |  | | |  | | |  | | |  | |  | |
| **Ceratozamia kuesteriana** Regel | Mexico (Tamaulipas) | CR |  |  | | | |  | | |  | | |  | | |  | | | x | | | x | | |  | |  | | | |  | | |  | | |  | |  | | |  | | | |  | | |  | | |  | | |  | |  | |
| **Ceratozamia latifolia** Miq. | Mexico (Hidalgo, Querétaro, San Luis Potosí, Veracruz) | EN | x | x | | | | x | | | x | | | x | | | x | | | x | | |  | | | x | | x | | | | x | | |  | | |  | |  | | |  | | | |  | | | x (f) | | |  | | |  | |  | |
| **Ceratozamia matudae** Lundell | Guatemala (Huehuetenango, San Marcos), Mexico (Chiapas) | EN |  |  | | | |  | | |  | | |  | | |  | | |  | | |  | | |  | |  | | | |  | | |  | | |  | |  | | |  | | | |  | | |  | | |  | | |  | |  | |
| **Ceratozamia mexicana** Brongn. | Mexico (Puebla, Veracruz) | VU | x | x (f) | | | | x | | |  | | | x | | |  | | | x | | |  | | |  | |  | | | |  | | |  | | |  | |  | | |  | | | |  | | |  | | |  | | |  | |  | |
| **Ceratozamia microstrobila** Vovides & J.D.Rees | Mexico (San Luis Potosí) | VU | x | x | | | | x | | |  | | |  | | |  | | |  | | |  | | |  | |  | | | |  | | |  | | |  | |  | | |  | | | |  | | |  | | |  | | |  | |  | |
| **Ceratozamia miqueliana** H.Wendl. | Mexico (Chiapas, Tabasco, Veracruz) | CR | x | x? | | | |  | | |  | | |  | | |  | | |  | | |  | | |  | |  | | | |  | | |  | | |  | |  | | |  | | | |  | | |  | | |  | | |  | |  | |
| **Ceratozamia mirandae** Vovides, Pérez-Farr. & Iglesias | Mexico (Chiapas) | EN |  |  | | | |  | | |  | | |  | | | x (f) | | |  | | |  | | |  | |  | | | |  | | |  | | |  | |  | | |  | | | |  | | |  | | |  | | |  | |  | |
| **Ceratozamia mixeorum** Chemnick, T.J.Greg. & Salas-Mor. | Mexico (Oaxaca) | EN | x |  | | | |  | | |  | | |  | | |  | | |  | | |  | | | x | |  | | | |  | | |  | | |  | |  | | |  | | | |  | | |  | | |  | | |  | |  | |
| **Ceratozamia morettii** Vázq.Torres & Vovides | Mexico (Veracruz) | EN | x |  | | | | x | | |  | | |  | | |  | | |  | | |  | | |  | |  | | | |  | | |  | | |  | |  | | |  | | | |  | | |  | | |  | | |  | |  | |
| **Ceratozamia norstogii** D.W.Stev. | Mexico (Chiapas, Oaxaca) | EN | x | x (f?) | | | |  | | |  | | |  | | |  | | |  | | |  | | |  | |  | | | |  | | |  | | |  | |  | | |  | | | |  | | |  | | |  | | |  | |  | |
| **Ceratozamia robusta** Miq. | Belize (Cayo, Stann Creek, Toledo), Guatemala (Alta Verapaz, Petén, Quiché, Huehuetenango, Izabal), Mexico (Chiapas, Oaxaca, Veracruz) | EN | x | x (f?) | | | |  | | |  | | | x | | | x | | | x | | |  | | |  | |  | | | |  | | |  | | |  | |  | | |  | | | |  | | |  | | |  | | |  | |  | |
| **Ceratozamia sabatoi** Vovides, Vázq.Torres, Schutzman & Iglesias | Mexico (Hidalgo, Querétaro) | EN | x | x (f?) | | | | x | | |  | | |  | | |  | | |  | | |  | | |  | |  | | | |  | | |  | | |  | |  | | |  | | | |  | | |  | | |  | | |  | |  | |
| **Ceratozamia santillanii** Pérez-Farr. & Vovides | Mexico (Chiapas) | CR | x | x? | | | |  | | |  | | |  | | |  | | |  | | |  | | |  | |  | | | |  | | |  | | |  | |  | | |  | | | |  | | |  | | |  | | |  | |  | |
| **Ceratozamia subroseophylla** Mart.-Domínguez & Nic.-Mor. | Mexico (Veracruz) | NE | x |  | | | |  | | |  | | | x | | |  | | |  | | |  | | |  | |  | | | |  | | |  | | |  | |  | | |  | | | |  | | |  | | |  | | |  | |  | |
| **Ceratozamia tenuis** (Dyer) D.W.Stev. & Vovides | Mexico (Veracruz) | n.a. | x |  | | | |  | | |  | | |  | | |  | | |  | | |  | | |  | |  | | | |  | | |  | | |  | |  | | |  | | | |  | | |  | | |  | | |  | |  | |
| **Ceratozamia totonacorum** Mart.-Domínguez & Nic.-Mor. | Mexico (Puebla) | NE | x |  | | | | x | | |  | | |  | | | x | | |  | | |  | | |  | |  | | | |  | | |  | | |  | |  | | |  | | | |  | | |  | | |  | | |  | |  | |
| **Ceratozamia vovidesii** Pérez-Farr. & Iglesias | Mexico (Chiapas) | VU | x | x (f?) | | | |  | | |  | | |  | | |  | | |  | | |  | | |  | |  | | | |  | | |  | | |  | |  | | |  | | | |  | | |  | | |  | | |  | |  | |
| **Ceratozamia whitelockiana** Chemnick & T.J.Greg. | Mexico (Oaxaca) | EN |  | x (f?) | | | |  | | |  | | | x? | | |  | | | x | | | x | | | x | |  | | | |  | | |  | | |  | |  | | |  | | | |  | | |  | | |  | | |  | |  | |
| **Ceratozamia zaragozae** Medellín | Mexico (San Luis Potosí) | CR |  |  | | | |  | | |  | | |  | | |  | | |  | | |  | | |  | |  | | | |  | | |  | | |  | |  | | |  | | | |  | | |  | | |  | | |  | |  | |
| **Ceratozamia zoquorum** Pérez-Farr., Vovides & Iglesias | Mexico (Chiapas) | CR | x | x? | | | |  | | |  | | |  | | |  | | |  | | |  | | |  | |  | | | |  | | |  | | |  | |  | | |  | | | |  | | |  | | |  | | |  | |  | |
| **Dioon angustifolium** Miq. | Mexico (Nuevo León, Tamaulipas) | VU | x | x | | | | x | | | x | | | x | | | x | | | x | | | x | | |  | |  | | | |  | | |  | | |  | | x | | |  | | | |  | | |  | | |  | | |  | |  | |
| **Dioon argenteum** T.J.Greg., Chemnick, Salas-Mor. & Vovides | Mexico (Oaxaca) | VU |  |  | | | |  | | |  | | |  | | |  | | |  | | |  | | |  | |  | | | |  | | |  | | |  | |  | | |  | | | |  | | |  | | |  | | |  | |  | |
| **Dioon califanoi** De Luca & Sabato | Mexico (Oaxaca, Puebla) | EN | x | x (f) | | | |  | | |  | | |  | | |  | | |  | | |  | | |  | |  | | | |  | | |  | | |  | |  | | |  | | | |  | | |  | | |  | | |  | |  | |
| **Dioon caputoi** De Luca, Sabato & Vázq.Torres | Mexico (Oaxaca, Puebla) | EN | x |  | | | |  | | |  | | |  | | |  | | |  | | |  | | |  | |  | | | |  | | |  | | |  | |  | | |  | | | |  | | |  | | |  | | |  | |  | |
| **Dioon edule** Lindl. | Mexico (Hidalgo, Querétaro, San Luis Potosí, Tamaulipas, Veracruz) | NT | x | x | | | | x | | | x | | | x | | | x | | |  | | | x | | | x | | x | | | |  | | |  | | | x | | x | | |  | | | |  | | |  | | |  | | | x (f) | |  | |
| **Dioon holmgrenii** De Luca, Sabato & Vázq.Torres | Mexico (Oaxaca) | EN | x | x (f) | | | |  | | |  | | | x | | | x | | |  | | |  | | |  | |  | | | |  | | |  | | |  | |  | | |  | | | |  | | |  | | |  | | |  | |  | |
| **Dioon mejiae** Standl. & L.O.Williams | Honduras (Colón, Olancho, Yoro) | LC | x | x | | | | x | | | x | | | x | | | x | | | x | | | x | | | x | | x | | | |  | | |  | | | x | |  | | |  | | | |  | | |  | | |  | | |  | | x | |
| **Dioon merolae** De Luca, Sabato & Vázq.Torres | Mexico (Chiapas, Oaxaca) | VU | x | x | | | | x | | | x | | | x | | | x | | |  | | | x | | |  | | x | | | |  | | |  | | |  | |  | | |  | | | |  | | |  | | |  | | |  | |  | |
| **Dioon planifolium** Salas-Mor., Chemnick & T.J.Greg. | Mexico (Oaxaca) | VU |  | x (f) | | | |  | | |  | | | x | | |  | | |  | | |  | | | x | |  | | | |  | | |  | | |  | |  | | |  | | | |  | | |  | | |  | | |  | |  | |
| **Dioon purpusii** Rose | Mexico (Oaxaca) | VU | x |  | | | | x | | |  | | |  | | |  | | |  | | |  | | |  | |  | | | |  | | |  | | |  | |  | | |  | | | |  | | |  | | |  | | |  | |  | |
| **Dioon rzedowskii** De Luca, Moretti, Sabato & Vázq.Torres | Mexico (Oaxaca) | EN | x |  | | | | x | | |  | | |  | | |  | | |  | | |  | | |  | |  | | | |  | | |  | | |  | |  | | |  | | | |  | | |  | | |  | | |  | |  | |
| **Dioon sonorense** (De Luca, Sabato & Vázq.Torres) Chemnick, T.J.Greg. & Salas-Mor. | Mexico (Sinaloa, Sonora) | EN | x |  | | | |  | | | x | | |  | | | x | | | x | | |  | | |  | |  | | | |  | | |  | | |  | | x | | | ? | | | |  | | | ? | | | ? | | |  | |  | |
| **Dioon spinulosum** Dyer ex Eichler | Mexico (Oaxaca, Veracruz) | EN | x | x | | | | x | | | x | | |  | | | x | | |  | | | x | | | x | |  | | | |  | | |  | | |  | |  | | |  | | | |  | | |  | | |  | | |  | |  | |
| **Dioon stevensonii** Nic.-Mor. & Vovides | Mexico (Guerrero, Michoacán) | CR | x |  | | | |  | | |  | | | x | | | x | | |  | | |  | | |  | |  | | | |  | | |  | | |  | |  | | |  | | | |  | | |  | | |  | | |  | |  | |
| **Dioon tomasellii** De Luca, Sabato & Vázq.Torres | Mexico (Durango, Jalisco, Nayarit) | VU | x | ? (f) | | | |  | | |  | | | x | | | x | | |  | | |  | | |  | |  | | | |  | | |  | | |  | |  | | |  | | | |  | | |  | | |  | | |  | |  | |
| **Zamia cremnophila** Vovides, Schutzman & Dehgan | Mexico (Tabasco) | EN |  |  | | | |  | | |  | | |  | | |  | | |  | | |  | | |  | |  | | | |  | | |  | | |  | |  | | |  | | | |  | | |  | | |  | | |  | |  | |
| **Zamia fischeri** Miq. | Mexico (Hidalgo, Querétaro, San Luis Potosí, Tamaulipas) | EN | x |  | | | | x | | |  | | | x | | | x | | | x | | |  | | | x | |  | | | | x | | |  | | | x | | x | | | x | | | |  | | |  | | |  | | |  | |  | |
| **Zamia furfuracea** L.f. | Mexico (Veracruz) | EN | x |  | | | |  | | |  | | |  | | |  | | |  | | |  | | |  | |  | | | |  | | |  | | |  | |  | | |  | | | |  | | |  | | |  | | |  | |  | |
| **Zamia grijalvensis** Pérez-Farr., Vovides & Mart.-Camilo | Mexico (Chiapas) | CR |  |  | | | |  | | |  | | |  | | |  | | |  | | |  | | |  | |  | | | |  | | |  | | |  | |  | | |  | | | |  | | |  | | |  | | |  | |  | |
| **Zamia herrerae** S.Calderón & Standl. | El Salvador (Sonsonate), Guatemala (Quetzaltenango Retalhuleu, Santa Rosa, Suchitepéquez), Mexico (Chiapas) | VU | x |  | | | |  | | |  | | | x | | |  | | |  | | |  | | |  | |  | | | |  | | | x | | |  | |  | | |  | | | |  | | |  | | |  | | |  | |  | |
| **Zamia inermis** Vovides, J.D.Rees & Vázq.Torres | Mexico (Veracruz) | CR | x |  | | | |  | | |  | | |  | | |  | | |  | | |  | | |  | |  | | | |  | | |  | | |  | |  | | |  | | | |  | | |  | | |  | | |  | |  | |
| **Zamia katzeriana** (Regel) E.Rettig | Mexico (Chiapas) | CR |  |  | | | |  | | |  | | |  | | |  | | |  | | |  | | |  | |  | | | |  | | |  | | |  | |  | | |  | | | |  | | |  | | |  | | |  | |  | |
| **Zamia lacandona** Schutzman & Vovides | Mexico (Chiapas) | EN |  |  | | | |  | | |  | | |  | | |  | | |  | | |  | | |  | |  | | | |  | | |  | | |  | |  | | |  | | | |  | | |  | | |  | | |  | |  | |
| **Zamia loddigesii** Miq. | Mexico (Chiapas, Hidalgo, Oaxaca, Tabasco, Tamaulipas, Veracruz) | NT | x | x | | | | x | | |  | | | x | | | x | | | x | | |  | | |  | |  | | | |  | | | x | | | x | | x | | | ? | | | | x | | |  | | |  | | |  | |  | |
| **Zamia meermanii** Calonje | Belize (Belize, Cayo, Toledo) | EN |  |  | | | |  | | |  | | |  | | |  | | |  | | |  | | |  | |  | | | |  | | |  | | |  | |  | | |  | | | |  | | |  | | |  | | |  | |  | |
| **Zamia monticola** Chamb. | Guatemala (Alta Verapaz) | CR | x |  | | | |  | | |  | | |  | | |  | | |  | | |  | | |  | |  | | | |  | | | x | | |  | |  | | |  | | | |  | | |  | | |  | | |  | |  | |
| **Zamia onan-reyesii** C.Nelson & Sandoval | Honduras (Cortés) | CR | x |  | | | |  | | |  | | | x | | |  | | |  | | |  | | |  | |  | | | |  | | | x | | |  | |  | | |  | | | |  | | |  | | |  | | |  | |  | |
| **Zamia oreillyi** C.Nelson | Honduras (Atlántida) | VU | x |  | | | |  | | |  | | | x | | |  | | |  | | |  | | |  | |  | | | |  | | | x | | |  | |  | | |  | | | |  | | |  | | |  | | |  | |  | |
| **Zamia paucijuga** Wieland | Mexico (Colima, Guerrero, Jalisco, Michoacán, Nayarit, Oaxaca) | NT | x |  | | | |  | | |  | | | x | | |  | | | x | | |  | | |  | |  | | | |  | | |  | | |  | | x | | | ? | | | |  | | |  | | |  | | |  | |  | |
| **Zamia prasina** W.Bull | Belize (Belize, Cayo, Orange Walk, Stann Creek, Toledo), Guatemala (Petén), Mexico (Campeche, Chiapas, Tabasco, Quintana Roo, Yucatán) | NT | x |  | | | |  | | |  | | | x | | |  | | |  | | |  | | |  | |  | | | |  | | | x | | |  | |  | | |  | | | |  | | |  | | |  | | |  | |  | |
| **Zamia purpurea** Vovides, J.D.Rees & Vázq.Torres | Mexico (Oaxaca, Veracruz) | CR |  |  | | | |  | | |  | | |  | | |  | | |  | | |  | | |  | |  | | | |  | | |  | | |  | |  | | |  | | | |  | | |  | | |  | | |  | |  | |
| **Zamia sandovalii** C.Nelson | Honduras (Atlántida) | NT | x |  | | | |  | | |  | | | x | | |  | | |  | | |  | | |  | |  | | | |  | | | x | | |  | |  | | |  | | | |  | | |  | | |  | | |  | |  | |
| **Zamia soconuscensis** Schutzman, Vovides & Dehgan | Mexico (Chiapas) | VU |  |  | | | |  | | |  | | |  | | |  | | |  | | |  | | |  | |  | | | |  | | |  | | |  | |  | | |  | | | |  | | |  | | |  | | |  | |  | |
| **Zamia spartea** A.DC. | Mexico (Oaxaca) | CR |  |  | | | |  | | |  | | |  | | |  | | |  | | |  | | |  | |  | | | |  | | |  | | |  | |  | | |  | | | |  | | |  | | |  | | |  | |  | |
| **Zamia splendens** Schutzman | Mexico (Chiapas, Tabasco, Veracruz) | EN | x |  | | | |  | | |  | | |  | | |  | | |  | | |  | | |  | |  | | | |  | | | ? | | |  | |  | | |  | | | |  | | |  | | |  | | |  | |  | |
| **Zamia standleyi** Schutzman | Guatemala (Izabal), Honduras (Atlántida, Colón, Cortés, Olancho, Santa Barbara, Yoro) | VU | x | x (f) | | | |  | | |  | | | x | | |  | | |  | | |  | | |  | |  | | | |  | | | x | | |  | |  | | |  | | | |  | | |  | | |  | | |  | |  | |
| **Zamia tuerckheimii** Donn.Sm. | Guatemala (Alta Verapaz) | NT | x |  | | | |  | | |  | | | x | | |  | | |  | | |  | | |  | |  | | | |  | | | x | | |  | |  | | |  | | | |  | | |  | | |  | | |  | |  | |
| **Zamia variegata** Warsz. | Belize (Toledo), Guatemala (Alta Verapaz, Izabal), Mexico (Chiapas) | EN | x |  | | | |  | | |  | | | x | | |  | | | x? | | |  | | |  | |  | | | |  | | | x | | |  | |  | | |  | | | | x | | |  | | |  | | |  | |  | |
| **Zamia vazquezii** D.W.Stev., Sabato & De Luca | Mexico (Veracruz) | CR | x |  | | | | x | | |  | | |  | | |  | | |  | | |  | | |  | |  | | | |  | | |  | | |  | |  | | |  | | | |  | | |  | | |  | | |  | |  | |
| **Zamia decumbens** Calonje, Meerman, M.P.Griff. & Hoese Images | Belize (Cayo, Stann Creek, Toledo) | CR | x |  | | | | x | | |  | | |  | | |  | | |  | | |  | | |  | |  | | | |  | | |  | | |  | |  | | |  | | | |  | | |  | | |  | | |  | |  | |
|  |  |  |  | |  | |  | | |  | | |  | |  | | | |  | | |  | | |  | | | |  | |  | | |  | | |  | | | |  | | |  | |  | |  | | | |  | | |  |  |  |  |  |
|  |  |  |  | |  | |  | | |  | | |  | |  | | | |  | | |  | | |  | | | |  | |  | | |  | | |  | | | |  | | |  | |  | |  | | | |  | | |  |  |  |  |  |

**Table S2 key**

^a^Taxonomy and IUCN categories follow [2]. ^b^Minor Uses and Beliefs: from ‘Other Uses’ in Supplementary Table 1. ^c^EN=Endangered; CR=Critically Endangered; VU=Vulnerable; NT=Near Threatened; LC=Least Concern; NE=Not Evaluated.

^d^x: use or belief recorded.(f): formerly. ?: suspected but unconfirmed.

**Additional References**

[104] Salas-Morales SH, Chemnick J, Gregory TJ. A new cycad species in the genus Dioon (Zamiaceae) from the Mixteca region of Oaxaca, Mexico. Cactus and Succulent Journal. 2016;88:35-42.

[105] Chemnick J, Gregory TJ. A new species of Ceratozamia (Zamiaceae) from Oaxaca, Mexico with comments on distribution, habitat, and relationships. Phytologia. 1995;79:51-7.

[106] Aguilar S. Etnobotánica cuantitativa en una región de bosque de niebla de Sierra Norte, Oaxaca. Mexico City: Instituto Politécnico Nacional; 2007.

[107] Perez-Farrera MA. Cycadales spp. in Chiapas, Mexico (Ceratozamia mirandae) NDF Workshop Case Studies WG 3 – Succulents and Cycads. http://www.cites.org/sites/default/files/ndf_material/WG3-CS2.pdf. Accessed 20 Jan 2018.

[108] Martínez-Domínguez L, Nicolalde-Morejón F, Stevenson DW, Vergara-Silva F. A new species of Ceratozamia (Zamiaceae) from the Sierra Norte of Puebla, Mexico. Brittonia. 2017;1-9.

[109] Hernández Sandoval L, González Romo CE, González Medrano F. Plantas útiles de Tamaulipas. Anales del Instituto de Biología. Serie Botánica. 1991;62.

[110] Amiguet VT, Arnason JT, Maquin P, Cal V, Sánchez-Vindas P, Alvarez LP. A regression analysis of Q’eqchi’ Maya medicinal plants from southern Belize. Econ. Bot. 2006;60:24-38.

[111] Vázquez Torres SM. 1990. Algunos datos etnobotánicos sobre las Zamiaceae (Cycadales) de México. Mem New York Botan G. 1990;57:144-147.

[112] Chemnick J, Gregory TJ, Salas Morales S. 1997. A revision of Dioon tomasellii (Zamiaceae) from western Mexico, a range extension of D. merolae, and clarification of D. purpusii. Phytologia. 1997;83:1-6.

[113] Cameras Velasco JC. Plan integral, bajo la modalidad de Unidad de Manejo Ambiental de la Facultad de Ciencias Biológicas y Agropecuarias, Tuxpan, Veracruz. Tuxpan: Univ Veracruzana; 2015.

[114] Larsen R. Vocabulario huasteco del estado de San Luis Potosi. 2nd ed. Mexico City: ILV; 1997.

[115] Martínez M. 1979. Catálogo de nombres vulgares y científicos de plantas mexicanas. Mexico City: Fondo de Cultura Económica; 1979.

[116] SEMARNAT. Programa de manejo de la Reserva de la Biosfera Selva El Ocote. Ocozocoautla de Espinosa, Chiapas; 2001.

[117] Pérez-Farrera MA, Espinosa Jiménez JA, López A, Gómez Domínguez H, Gordillo Ruíz MC. Flora y vegetación de la selva zoque de Chiapas. In: SEMAHN. Ecoregión zoque, retos y oportunidades ante el cambio climático. Tuxtla Gutierrez: Secretaria de Medio Ambiente e Historia Natural. p. 52-76.

[118] Harrison RH, García M, Harrison CR, Harrison M, Cástulo García H. Diccionario zoque de Copainalá. Coyoacán: ILV; 1981.

[119] Standley PC, Steyermark JA. Flora of Guatemala. Cycadaceae. Fieldiana Bot. 1958;24:11-20.

[120] Goodwin ZA, Lopez GN, Stuart N, Bridgewater SG, Haston EM, Cameron ID, Michelakis D, Ratter JA, Furley PA, Kay E, Whitefoord C. A checklist of the vascular plants of the lowland savannas of Belize, Central America. Phytotaxa. 2013;101:1-19.

[121] Consejo Nacional de Areas Protegidas-CONAP. Lista de especies amenazadas de Guatemala-LEA-y listado de especies de flora y fauna silvestres CITES de Guatemala. 2nd ed. Guatemala City: CONAP; 2009.

[122] Wisdom C. The Chorti indians of Guatemala. Chicago: U Chicago P; 1940.

# [123] Conzemius E. Ethnographical survey of the Miskito and Sumu indians of Honduras and Nicaragua. Bur Am Ethnol Bull. 1932;106:1-191.

[124] Véliz M, Barrios A, Dávila C. La familia Zamiaceae en Guatemala. Revista Científica. 2014; 4.

[125] Chemnick J, Gregory TJ, Salas Morales S. Ceratozamia mixeorum (Zamiaceae), a new species from Oaxaca, Mexico with comments on distribution, habitat, and species relationships. Phytologia. 1997;83:47-52.

[126] Anderson EN, Cauich CJ, Dzib A, Flores GS, Isabele G, Medina TF, Sanchez SO, Valdez CP. 2005. Las plantas de los Mayas: etnobotánica en Quintana Roo. Mexico City: CONABIO-ECOSUR; 2005.

[127] Méndez RM. El huerto familiar del sureste de México. Villahermosa: Secretaría de Recursos Naturales y Protección Ambiental el Estado de Tabasco; 2012.

[128] SEMARNAT. Manifestación de impacto ambiental, modalidad regional Los Arboles Tulum. 2005. http://sinat.semarnat.gob.mx/dgiraDocs/documentos/qroo/estudios/2005/23QR2005T0046.pdf. Accessed 20 Jan 2018.

[129] Martínez M. Las plantas medicinales de México. 3rd edition. Mexico: Ed Botanicas; 1944.

[130] Standley PC. Trees and shrubs of Mexico (Cycadaceae). Contributions from the United States National Herbarium. 1920;23:47-50.

[131] Martinez-Dominguez L, Nicolalde-Morejon F, Stevenson DW. Qualitative and quantitative morphological evidence for recognition of a new species within Ceratozamia (Zamiaceae) from Mexico. Phytotaxa. 2017;317:17-28.

[132] Castañeda ES. Parámetros demográficos de Dioon spinulosum Dyer & Eichler (Zamiaceae), en San Miguel Soyaltepec, Oaxaca. Xalapa, Veracruz: Instituto de Ecología; 2009.

[133] Merrifield WR, Andreson AE. Diccionario Chinanteco de la diáspora del pueblo antiguo de San Pedro Tlatepuzco, Oaxaca. Coyoacán: ILV; 1999.

[134] Calonje M, Meerman J, Griffith P, Hoese G. A new species of Zamia (Zamiaceae) from the Maya Mountains of Belize. J Bot Res Inst Texas. 2009;31-41.

[135] Chemin Bassler H. Los pames septentrionales de San Luís Potosí. Mexico City: Instituto Nacional Indigenista; 1984.

[136] Lázaro-Zermeño JM, González-Espinosa M, Mendoza A, Martínez-Ramos M. Historia natural de Dioon merolae (Zamiaceae) en Chiapas, México. Bot Sci. 2012;90:73-87.

[137] Avedaño Reyes S, Flores Gudiño JS. 1999. Registro de plantas tóxicas para ganado en el estado de Veracruz, México. Vet Mex. 1999;30.

[138] Belmar F. Estudio de el Chontal. Oaxaca; 1900.

[139] Recursos Naturales y Pesca SEMARNAP. Catálogo de especies vulnerables al aprovechamiento forestal en bosques templados del estado de Oaxaca. Mexico: PROCYMAF; 2000.

[140] Perez P. 2003. Lexique lacandon (maya) francais/espagnol. Toulouse: Ministere de la Culture et Centre National de la Recherche Scientifique MAP, Unite Mixte de Recherche; 2003.

[141] Chamberlain CJ. The ovule and female gametophyte of Dioon. Bot Gaz. 1906;42:321-58.

[142] Yetman D. The guarijios of the Sierra Madre: hidden people of northwestern Mexico. Albuquerque: U New Mexico P; 2002.

[143] Gentry HS. Rio Mayo plants. Carnegie I Wash. 1942;527.

[144] De Luca P, Sabato S, Torres MV. Dion tomasellii (Zamiaceae), a new species with two varieties from western Mexico. Brittonia. 1984;36:223-7.

[145] Gregory TJ, Chemnick J, Salas Morales SH, Walters T. The results of the MBC, SERBO, LOT, and INECOL 2004 cycad expedition to western Mexico. Field report; 2007. Coral Gables, Florida: Montgomery Botanical Center.

[146] Schoenhals LC. A Spanish-English glossary of Mexican flora and fauna. Mexico: Instituto Lingüístico de Verano; 1988.

[147] Vovides AP, Resslar PM, Pérez-López R. The cycads of Veracruz, Mexico. Norfolk, VA: El Eco de Virginia Bilingual Publ; 1992.

[148] De Luca P, Sabato S, Torres MV. Dioon holmgrenii (Zamiaceae), a new species from Mexico. Brittonia. 1981;33(4):552-5.

[149] SEMARNAT-CONANP. Sierra de Manantlán. https://simec.conanp.gob.mx/ficha_pdf.php?anp=59&reg=. Accessed 20 Jan 2018.

[150] Miranda F. Observaciones botanicas en la region de Tuxtepec, Oaxaca, con notas sobre plantas útiles. An Inst Biol Mex. 1948;19:105-36.

[151] Gamboa López A, García CR. Diccionario enciclopédico de Chiapas. Tuxtla Gutierrez: Consejo Estatal para la Cultura y las Artes de Chiapas; 2000.

[152] Pulido MT, Vargas-Zenteno M, Vite A, Vovides AP. 2015. Range extension of the endangered Mexican cycad *Ceratozamia fuscoviridis* Moore (teosintle): implications for conservation. Trop Conserv Sci. 2015;8:778-95.

[153] Vite A, Pulido MT, Flores JC. 2010. Aspectos etnobotánicos de las cícadas en algunas zonas de Hidalgo, México*.* In: Moreno A, Pulido MT, Mariaca R, Valadéz Azúa R, Mejía Correa P, Gutiérrez Santillán V, editors. Sistemas biocognitivos tradicionales: paradigmas en la conservación biológica y el fortalecimiento cultural. Asoc Etnobiol Mex, Global Diversity Foundation, Univ Aut Estado Hidalgo, Colegio Frontera Sur, Soc Latinoam Etnobiol. p. 481-86.

[154] Vázquez Torres M, Vovides AP. 1998. A new species of Ceratozamia (Zamiaceae) from Veracruz, Mexico. Novon. 1998;8:87-90.

[155] Medina Lemos R, Dávila P. Flora del Valle de Tehuacán-Cuicatlán. Fascículo 12. Gymnospermae Lindl. Mexico City: Instituto de Biología, UNAM; 1997.

[156] De Luca P, Moretti A, Sabato S, Torres MV. Dioon rzedowskii (Zamiaceae), a new species from Mexico. Brittonia. 1980;32:225-9.

[157] Capen CJ. Diccionario mazateco de Chiquihuitlan Oaxaca. Tucson: SIL; 1996.

[158] Escobar Ledesma A. Chical gastronomía queretana. Mexico City: CONACULTA; 2005.

[159] Upper Necaxa Totonaco project. Ethnobotany. http://www.artsrn.ualberta.ca/totonaco2/?page_id=1782. Accessed 20 Jan 2018.

[160] Aschmann HP. Diccionario totonaco de Papantla, Veracruz. Mexico City: ILV; 1973.

[161] McMahon A, de McMahon MA. Cora y español. Mexico City: ILV; 1959.

[162] de Candolle A. Prodromus systematis naturalis regni vegetabilis, part 16. Paris: Victor Masson; 1864-868.

[163] Ramírez J, Alcocer GV. Sinonimia vulgar y científica de las plantas mexicanas. Mexico City: Oficina Tipográfica de la Secretaría del Fomento; 1902.

[164] Chávez González AD, Rendóm Aguilar B, Luna José ADL. Ecología de Ceratozamia aff. Robusta en la Sierra Madre del Sur de Oaxaca. In: Vovides AP, Stevenson DW, Osborne R, editors. Proceedings of the seventh international conference on cycad biology (Xalapa, Mexico, 2005). Mem New York Botan G. 2007;97:74-86.

[165] Jose AD, Aguilar BR. Traditional knowledge among Zapotecs of Sierra Madre Del Sur, Oaxaca. Does it represent a base for plant resources management and conservation? J Ethnobiol Ethnomed. 2012;8:24. https://doi.org/10.1186/1746-4269-8-24
