# Supplementary material for: Ethnobotany of Mexican and northern Central American cycads (Zamiaceae)
Source: J Ethnobiol Ethnomed. 2019 Jan 18;15:4. doi: 10.1186/s13002-018-0282-z (PMC6339304; doi:10.1186/s13002-018-0282-z)
Supplement: Supplementary file 2 — Ethnographic Methods and Questions for Cycad Research. Contains two documents. ‘Ethnobotanical research on cycads in Mexico: Methods and suggested topics’ (Notes for field assistants and collaborators prepared by Mark Bonta, 2008–2014) and ‘Cycad Ethnobotany Questionnaire’ (Aurelia Vite Reyes [45]). (DOCX 47 kb) [file 13002_2018_282_MOESM2_ESM.docx]

**Ethnographic Methods and Questions for Cycad Research**

**Ethnobotanical research on cycads in Mexico: Methods and suggested topics**Notes for field assistants and collaborators prepared by Mark Bonta, 2008-2014; original in Spanish.

A mixture of open ethnographic interviews and questions prepared in questionnaire form is recommended. Short visits should be combined with participant observation, where feasible, and with the necessary permissions in hand. For example, Holy Week is a good time to witness or participate in ceremonies involving cycads. However, the rest of the year, through interviews, one can reconstruct the memory of ceremonies among key informants.

What follows is not necessarily appropriate for all situations, and it is not necessary to strictly follow the order of the list. In many cases, the schematic can be used to organize data after researchers’ stays in the field. In the selection and study of informants and interviews, we place particular emphasis on gender and age – of the researcher, the subject (the informant/ collaborator), other people present, and the cultural context. These variables can have substantial influence on the structure of the interview and the knowledge evinced/what is narrated. It is very important to try to establish specific historical contexts for data comparison to other places. For example, there is often a correlation of dates related to the scarcity of maize, and subsequent consumption of cycads, across a region, and thus associated with a historical phenomenon such as war or drought.

In ethnographic research, it is very important to protect the identity of the subject: besides the process of asking permission and in some cases the signing of documents, in most cases it is not necessary to retain the names of interviewees. At the same time, it is important to respect all cultural rules and try not to create any offensive situations. However, within the system of mutual respect, one must try to confirm information given by interviewees with other informants - and at the end of the research visit, use judgment to try to determine the validity of certain answers.

In one case, we found that respondents who did not have to consume cycads in times of scarcity, half a century ago, said they knew nothing of the matter and that the plant is not consumed; in the same community, however, were others who had experienced food shortages in their families, and they could describe exactly how cycads had been prepared. This demonstrates the need to interview members of different local social classes, and to utilize open interviews and questionnaires, so that respondents can also feel comfortable talking about the broader issues and the social history of the community in the context of which cycads are or have been utilized.

Obviously there are cases of untruths or simply forgetful informants, so often lengthy discussions help clarify many questions - particularly when dealing with local names and their meanings. Collection of local plant casually by collectors and botanists has caused much confusion, as these outsiders often teach people the “correct names” for cycads. You need to focus carefully on pronunciation (recording the name is optimal) but also on the meaning of the name, even if it is in Spanish. While the meaning may seem obvious, often it has more than one interpretation at the local level.

Finally, you should be very cautious in the detection of “traditional” ethnobotanical knowledge resulting from recent contacts – this is a type of “contamination” of traditional knowledge and is significant in itself- not *a priori* “good” or “bad”- but nevertheless, it can cause much confusion later. We must contemplate the origin of human knowledge - not everything is “tradition” but rather may come from the imagination of the individual person, according to their own experiences (idiosyncratic) and can also be derived from diffusion and acculturation, even if passed off as local knowledge. For cycads, for example, it is obvious that when an informant speaks of “la cica” without prompting, they have spoken with someone from outside or heard something that does not come from traditional local context. One not infrequently encounters informants who feel that outside knowledge is more valuable and “correct” than the traditions of their own communities, and even that local, traditional knowledge is shameful, a hallmark of backwardness. We have found this particularly to be the case among non-Catholic informants in communities (*e.g.,* Teenek) where Protestant evangelical religions have made recent headway – older knowledges and traditions are actively avoided or even ridiculed.

The following is a master list of data types relevant to relationships between people, cycads, maize, and the landscape. It is based on prior experience of uses that cycads tend to have, beliefs about cycads that people tend to hold, and so forth, in Mexican communities. Questions should be extracted and fashioned from this guide based on level of permission granted by informant and type of informant.

It is expected in all cases that type and level of permission granted will be defined and codified, as per IRB specifications in approved protocol, prior to any data-gathering. All other conditions of data availability, availability of interpreted results, level of availability to other researchers or to general public, necessary anonymization of data, and length of time that identifying data are retained, also apply. The default method of recording data is via writing in field notebooks and/or typing electronically.

Preliminary Data

Informants are approached with the aid of visual cues, typically printed or digital photos of local cycads, and asked if they are familiar with the plants. If they answer in the affirmative, an explanation of the research follows the IRB-approved protocol for garnering consent (typically, in Indigenous communities, researchers are accompanied by an official or representative of an official, and/or interviews may take place in a group meeting setting). Depending on the conditions imposed by the community and/or the potential informant, the following data are gathered for indexing purposes:

*Geolocation, Name of location, Age of informant, Gender of informant, Profession/s of informant, Researcher’s brief qualitative assessment of informant vis-à-vis potential to contribute data to the project.*

These are protected data and are used for a determination by the researcher, in a discussion with the informant and other locally-involved parties (the informant’s family, community authorities, etc.) (in consultation with project collaborators, if deemed necessary) of whether it seems worthwhile to enlist the potential informant in the study.

If consent is garnered and the potential informant has been provided relevant details about the study protocols, the interview process proceeds in a selective manner, via questions derived from the data categories below—targeted to the type of informant.

Taxonomy & Vocabulary: One of the most important sets of clues to the relationships between cycads and maize are the retained words related to cycads, even in cases where the meanings are unknown/forgotten. These are typically data gathered from all informants.

- *Local Name/s of cycads and cycad parts in Spanish, Nahua, or other languages.* Informants are asked to name male and female cones and cone parts, leaves, trunks, and other components.
- *Ethnic Group of informant (self-identified; government-identified).* The difference is important: informants may consider themselves to be mestizo but may be aware of the fact that “they say” that they are members of a certain ethnicity. Fluid identity categories are important to capture.
- *Generic Location / Specific Place.* Local toponyms that refer to cycads.
- *Spanish translation of non-Spanish words for cycad names and cycad toponyms* (offered by informant; offered by translator; suggested by other sources)

Ethnomedicinal Uses: Cycad parts are sometimes used for medicinal purposes – the sticky resin on the cones may serve as the component of a poultice, for example.

- *Detailed description of the medical use (protecting any secret information):* part used; ailment/s treated, role/s of the informant, beliefs regarding the efficacy of the treatment. Alcorn (1984) is a useful comparative source.

Decorative Uses: Cycad parts, typically leaves, are utilized widely in Mesoamerica for decoration during religious and civil events. Careful documentation of these events may sometimes be possibly as the events are occurring (an ideal case) but also afterward, as decorations are typically left up (for example, in shrines and on church facades) for weeks and even months after the event.

- *Event/s in which cycad parts are utilized* (separate descriptions of each event)
- *Process of gathering cycad parts.* This may range from informal gathering of leaves, to highly formal and ritualized events. Description of this process, and participation in it, if possible, is among the most valuable information in this project. Where it occurs/who does it/what takes place/what feelings people associate with the gathering process/what parts of plant are used.
- *Associated plants*. Careful documentation of plants that are typically combined with cycads. This requires knowledge of local names, and gathering of botanical samples, at minimum, of unfamiliar species.
- *Elaboration of the decoration/s*. Who does it; how is it done; why is it done; where is it done; when is it done.

Ornamental Uses (for Live Plants): These uses are important to document for communities, as they affect how researchers can collaborate with communities as communities decide to adopt or modify conservation measures. Traffic in Mexican cycads can be a valuable source of income for a community, and/or be devastating to cycad populations. The data do not, per se, have much value for determining relationships between cycads and maize beyond determining that certain ornamental uses (e.g., recent plantings in public parks) may have no deep symbolic value, but without asking, could be assumed to have this value (“over-interpretation” of the place of cycads)

- *Local ornamental uses*. Cycads are often planted in public parks or in yards, simply for their showiness.
- *Commercial Nurseries*. Researchers often visit nurseries to determine what cycads might be present locally.
- *Internal Trade*. Determine whether any trade in cycads or cycad parts exists, whether for profit or for other reasons.
- *External Trade*. Determine whether any cycads or cycad parts are exported (typically to the US) or sold to cities outside the local region.
- *Economy - quantitative details*. Determine, in a general sense, what the impact of any trade in cycads or cycads parts has on the community of the informant.

Alimentary Uses: In some study areas, cycads are rarely, if ever, still consumed, so oral historical documentation is paramount. Where the practice is current, careful documentation of the entire process of harvest and preparation, particularly through participant-observation, can reveal relationships with maize that are not readily apparent through interviews.

- *Associated plants*: leaves used for tamale wraps; ash for detoxification, etc.
- *Part/s consumed*: sarcotesta; endosperm; leaf; trunk; underground stem
- *Current uses*: document harvest – storage – preparation
- *Relative importance in diet*: what times of year/what types of occasions eaten, accompanied by what, how much harvested/consumed in the course of a year
- *Knowledge of and experiences with toxicity*
- *Taste and texture*

Domestic Miscellaneous Uses: A wide variety of minor uses; typical documentation includes part used, what it is/was used for, what is the process of elaboration

-Construction materials (eg roofs), Gums, Starches, Coloring agents/dye, Uses for domestic animals (e.g. food), Pesticides, Toys, Other miscellaneous uses

General Culture
-General state of perceptions
-General state of use (more now, less now, why less, what is replacing it, etc.)

Political Associations
-Tenure of land on which it grows
-Harvesting rights
-Punishment for infractions (illegal extraction, etc.)
-Local perceptions

-Rules governing cycads

Economy
-Value of traditional harvests
-Details of trade in cycads
-Economic history
-Importance in the local economy

Ethnoecology
-Overall 'management' characterization of population
-Control of fire
-Associations with cattle
-Protected, destroyed, ignored?
-Associations with agriculture
-Elite management (for example in private gardens)
-Agrosilvopastoral characterization of associated landscape
-Knowledges of predators
-Knowledges of pollinization, reproductive cycle
-Knowledge of cycad longevity
-Relationship to palms
-Relationship to maize
-Relationship to tubers: such as *Maranta* and *Ipomoea*
-Knowledge of dispersal agents
-General description of plant

Beliefs
-Anything not utilitarian or materialistic
-Relationship with cosmogony
-Incorporation in myths
-Sayings
-Folklore
-Symbolism

Geography of Cultivated Cycads
-In public spaces? Describe, document: churches, along roads, in city parks, etc. When? Why?
-In graveyards?
-In private spaces
- Populations planted along trade routes (*caminos reales*), in suburbs, etc.

Geography of Wild Cycads
-Local maps of populations: Elaborate in collaboration with informants
-Maps showing spatial relationships of populations to users
-Toponyms (names of places)

Historical and Archaeological Sources
-Oral interviews (local)
-Iconography: churches, pottery, codices, maps
-Local maps (eg AGNM)
-Geographical Relations (Relaciones Geográficas)
-Spanish royal chroniclers
-Local accounts by friars and others
-In travelers' accounts
-Archaeological excavations in cycad areas: field reports
-Municipal monographs

**Cycad Ethnobotany Questionnaire**

Extracted (p. 101-4) from Vite Reyes A. Etnobotánica de cícadas en comunidades nahuas y mestizas de Tlanchinol, Hidalgo [Master’s thesis]. Pachuca: Univ Aut Estado Hidalgo; 2012.

**ANEXO B**. Guía de entrevista

Lugar: Fecha

Nombre:_ Ocupación:

Grupo cultural al que pertenece:_

**Entrevista general**

1. ¿Conoce la planta?

2. ¿Con qué nombre la conoce?

3. ¿Por qué se le llama así / por qué da ese nombre?

4. ¿Qué otros nombres tiene?

5. ¿Cuántos tipos de esta planta conoce?

6. ¿Conoce otras plantas de este mismo tipo / otras plantas relacionadas con este teocinte?

7. ¿Qué tienen de parecido?

8. ¿Cómo las diferencia? Aspectos biológicos

9. ¿En relación a la planta del maíz, esta clase de plantas crecen rápido o crecen lento?

10. ¿Cuánto tiempo tarda en crecer una planta de un metro aproximadamente?

11. ¿Cuánto tiempo puede tardar una planta, desde que es chiquita hasta que tenga su mazorquita (piña)?

12. ¿En qué meses se produce la mazorquita (piña)?

13. ¿Cuántas veces al año se da la mazorquita (piña)?

14. ¿Tienen algún nombre las partes de la planta (hojas, tallo, estróbilo)?

15. ¿Han visto si las semillas u otras partes de la planta sean comidas por los animales?

16. ¿Qué animales ha visto sobre la planta?

17. ¿En qué tipo de suelos se encuentran?

18. ¿En qué tipo de montes se encuentran?

Las prácticas y frecuencia de uso

1. ¿Utilizan la planta?

2. ¿Para qué las utilizan?/ Por qué razón hacen el arco?, Qué significa?

3. ¿Antes tenía otros usos?

4. ¿En qué fechas y en qué tipo de celebraciones se usan?

5. ¿Cuántas veces al año las utilizan?

6. ¿Qué partes de la planta usan?

7. ¿Todas las plantas son útiles?

8. ¿Desde cuándo las usan?

9. ¿Las usan desde que usted era niño?

Elaboración de arcos decorativos

10. ¿Cuál es el proceso de uso?

11. ¿Qué tipo de palos utilizan para hacer la base del arco?

12. ¿Qué es lo que utilizan para amarrar la base?

13. ¿Por qué usan las hojas de esa planta y no otra?

14. ¿Conoce a alguien más que utilice la planta para algo?

15. ¿Con qué otras plantas usan el teocinte?

**ENTREVISTAS SÓLO PARA LOS QUE HAN PARCIPADO EN LA ELABORACIÓN DE ARCOS**

1. ¿Cuál es el proceso de uso/ armado del arco?

2. ¿Qué tipo de palos utilizan para hacer la base del arco?

3. ¿Qué es lo que utilizan para amarrar la base?

4. ¿Por qué usan las hojas de esa planta y no otra?

5. ¿Conoce a alguien más que utilice la planta para algo?

6. ¿Con qué otras plantas usan el teosinte?

7. ¿Estas plantas las compran o de donde las traen?

8. ¿Cómo las ponen (posición) con las hojas?

**ENTREVISTAS SÓLO PARA LOS COLECTORES DE HOJAS**

1. ¿Hasta dónde van por las hojas?

2. ¿Cómo se llama el lugar?

3. ¿A qué distancia se encuentra de su comunidad?

4. ¿Sabe de gente de otro lado que vengan a cortar hojas aquí?

5. ¿Sabe de dónde vienen?

6. ¿Estas personas tienen que pedir algún permiso para poderse llevarse las hojas?

7. ¿Pagan alguna cantidad a la comunidad ó tal vez lo cambian por otra cosa?

8. ¿Antes se hacía algo de esto?

9. ¿Cómo cortan las hojas?

10. ¿Cuántas hojas cortan por planta?

11. ¿Cómo deben de ser las hojas (características) para que las corten?

12. ¿Cuántas veces al año se realizan las cosechas de hojas?

13. ¿Cuántas personas van por las hojas

**Formas de manejo**

1. ¿Hace algo para propagar la planta (semillas o por medio de la remoción de toda la planta)?

2. ¿La dejan en algún sitio (milpas, potreros, jardines)?

3. ¿Debe cuidarse de alguna forma?

**Percepción de las cícadas**

Empezar a hablar sobre las plantas en general y de ahí pasar a las cícadas

1. ¿Le han echado mal de ojo a alguna de sus plantas?

2. ¿Usted sabe si por envidia los cultivos se han secado?

3. ¿Las plantas se enferman?

4. ¿Le alegran el día?

5. ¿Usted les habla a estas plantas?

6. ¿Qué es lo que distingue a las plantas?/La considera una planta fría o una planta caliente?

7. ¿El olor que despide tiene algún efecto?, /manifiesta algo?

8. ¿Las plantas tienen alma?

9. ¿Sabe de alguna historia o cuento sobre el teocintle?
